# Clinical application of genomic profiling to find druggable targets for adolescent and young adult (AYA) cancer patients with metastasis

**DOI:** 10.1186/s12885-016-2209-1

**Published:** 2016-02-29

**Authors:** Soojin Cha, Jeongeun Lee, Jong-Yeon Shin, Ji-Yeon Kim, Sung Hoon Sim, Bhumsuk Keam, Tae Min Kim, Dong-Wan Kim, Dae Seog Heo, Se-Hoon Lee, Jong-Il Kim

**Affiliations:** Cancer Research Institute, Seoul National University College of Medicine, Seoul, Republic of Korea; Interdisciplinary Program for Bioengineering of Graduate School, Seoul National University, Seoul, Republic of Korea; Genomic Medicine Institute, Seoul National University, Seoul, Republic of Korea; Department of Internal Medicine, Seoul National University Hospital, Seoul, Republic of Korea; Department of Biomedical Sciences, Seoul National University Graduate School, Seoul, Republic of Korea; Department of Biochemistry and Molecular Biology, Seoul National University College of Medicine, Seoul, Republic of Korea; Division of Hematology/Oncology, Department of Medicine, Samsung Medical Center, Sungkyunkwan University School of Medicine, Seoul, South Korea; Department of Health Sciences and Technology, SAIHST, Sungkyunkwan University, Seoul, South Korea

**Keywords:** Adolescent and young adult (AYA) cancer, Next-generation sequencing (NGS), Whole exome sequencing, Precision medicine, Genomics

## Abstract

**Background:**

Although adolescent and young adult (AYA) cancers are characterized by biological features and clinical outcomes distinct from those of other age groups, the molecular profile of AYA cancers has not been well defined. In this study, we analyzed cancer genomes from rare types of metastatic AYA cancers to identify driving and/or druggable genetic alterations.

**Methods:**

Prospectively collected AYA tumor samples from seven different patients were analyzed using three different genomics platforms (whole-exome sequencing, whole-transcriptome sequencing or OncoScan™). Using well-known bioinformatics tools (bwa, Picard, GATK, MuTect, and Somatic Indel Detector) and our annotation approach with open access databases (DAVID and DGIdb), we processed sequencing data and identified driving genetic alterations and their druggability.

**Results:**

The mutation frequencies of AYA cancers were lower than those of other adult cancers (median = 0.56), except for a germ cell tumor with hypermutation. We identified patient-specific genetic alterations in candidate driving genes: *RASA2* and *NF1* (prostate cancer), *TP53* and *CDKN2C* (olfactory neuroblastoma), *FAT1, NOTCH1*, and *SMAD4* (head and neck cancer), *KRAS* (urachal carcinoma), *EML4-ALK* (lung cancer), and *MDM2* and *PTEN* (liposarcoma). We then suggested potential drugs for each patient according to his or her altered genes and related pathways. By comparing candidate driving genes between AYA cancers and those from all age groups for the same type of cancer, we identified different driving genes in prostate cancer and a germ cell tumor in AYAs compared with all age groups, whereas three common alterations (*TP53*, *FAT1*, and *NOTCH1*) in head and neck cancer were identified in both groups.

**Conclusion:**

We identified the patient-specific genetic alterations and druggability of seven rare types of AYA cancers using three genomics platforms. Additionally, genetic alterations in cancers from AYA and those from all age groups varied by cancer type.

**Electronic supplementary material:**

The online version of this article (doi:10.1186/s12885-016-2209-1) contains supplementary material, which is available to authorized users.

## Background

Cancer is one of the leading causes of death worldwide. Abnormal genetic alterations followed by the uncontrolled growth of somatic cells initiate cancer. Although most genetic alterations are passenger mutations that do not contribute to tumorigenesis, an individual cell can proliferate and become a tumor if it acquires a sufficient set of driving mutations. Therefore, finding cancer-driving mutations and targeting the encoded abnormal proteins and related pathways via cancer therapeutics are important strategies to delay cancer progression and prevent metastasis [1].

Previous studies, led by The Cancer Genome Atlas (TCGA) and International Cancer Genome Consortium (ICGC), have identified cancer-driving mutations via large-scale analyses [2]. Although large-scale analyses unveiled frequently altered driving mutations in many cancer types, such as *BRAF* (V600E) in melanoma and colorectal cancer, finding less frequently altered mutations is a challenge using large-scale analyses, especially in uncommon cancer types [2–4].

Adolescent and young adult (AYA) cancer is a rare type of malignant disease that arises in patients aged 15 to 39 years and is characterized by biological features, therapeutic outcomes, and survival rates that are distinct from those observed in other age groups. Although determining the genomic profiles of AYA cancer is important to investigate the causes of these distinct characteristics, large-scale genomic studies or molecular data for AYA cancer are not available due to the rarity of the disease and the difficulty of collecting tumor samples [5, 6].

In this study, we analyzed seven different AYA cancers from patients with metastatic tumors using three different genomics platforms (whole-exome sequencing, whole-transcriptome sequencing, and OncoScan™). We identified single nucleotide variations (SNVs) and insertion and deletions (indels) by using whole-exome sequencing (WES) and detected fusions by using whole transcriptome sequencing (WTS). For copy number variations (CNVs), we used OncoScan™ that is the genomics platform for analysis of copy number variations which had high performance with samples from FFPE, especially [7]. We processed the WES data with well-known bioinformatics tools (bwa, Picard, GATK, MuTect, and Somatic Indel Detector), as other studies described and processed WTS data with fusion detection tools [8–10]. We then identified candidate genes and suggested potential drugs that are specific to the genetic alterations of each patient. We also compared candidate genes for AYA cancers with the same types of cancers from all age groups using published data.

## Methods

### Ethics and consent statement

This study was approved by the Institutional Review Board (IRB) of Seoul National University Hospital (1206-086-414). We obtained written informed consent from the patients who participated to this study. All participants in this study gave us written informed consent for publication of their details. Written informed consent for publication of their clinical details and/or clinical images was obtained from the patients. A copy of the consent form is available for review by the Editor of this journal.

### Study design and sample information

Samples from seven different tumors, prostate cancer, olfactory neuroblastoma, head and neck squamous cell carcinoma (HNSCC), urachal carcinoma, germ cell tumor, lung cancer, and liposarcoma, were prospectively obtained in three different forms (fresh-frozen tissue, formalin-fixed paraffin-embedded (FFPE), and pleurisy). The samples were analyzed using three different genomics platforms (whole-exome sequencing (WES), whole transcriptome sequencing (WTS), and OncoScan™) as the tumor sources permitted. We first intended to analyze samples from ten patients, but three samples were excluded because the amount of provided tumor sample was insufficient (AYA03) or sufficient DNA/RNA for a genome-scale analysis was not obtained (AYA05, and 08). For sample AYA04 (HNSCC), the HPV infection status was identified by IHC staining (data not shown).

### Whole exome sequencing (WES)

A minimum of 3 μg of genomic DNA was randomly fragmented by Covaris, and the sizes of the library fragments were mainly distributed between 250 and 300 bp. adapters were then ligated to both ends of the fragments. Extracted DNA was amplified by ligation-mediated PCR (LM-PCR) and then purified and hybridized to the SureSelect XT Human All Exon v4 + UTR 71 Mb (Agilent Technologies, Santa Clara, CA, USA) for enrichment according to the manufacturer’s recommended protocol. After loading each captured library on the Hiseq2000 platform (Illumina, San Diego, CA, USA), we performed high-throughput sequencing for each captured library. Raw image files were processed by Illumina CASAVA v1.8.2 for base-calling with default parameters, and the sequences from each individual were generated as 101-bp pair-end reads.

### Processing WES data to analyze SNVs and indels

WES data were processed using a series of steps. We aligned the sequenced files (Fastq file) to the reference genome (human reference genome g1k v37) using the Burrows-Wheeler Aligner (BWA v0.7.5a) [11] and then sorted the output and removed PCR duplicates using PICARD v1.95 [12]. Using the typical GATK workflow (The Genome Analysis Toolkit v2.6-5), we processed the data for local indel realignment and base quality recalibration [13]. For variant calling, we used MuTect v1.1.6 for single nucleotide variants (SNVs) and Somatic Indel Detector (from GATK v2.2-8) for indels [14]. Whereas we called the SNVs with the default setting value, we altered the tumor indel fraction from 0.3 to 0.05 (T_INDEL_*F* <0.05) for indel calling after considering false-negatives. The called variants interpreted as somatic mutations were tagged with “KEEP” or “SOMATIC” with MuTect and Somatic Indel Detector, respectively, and used for further study. To avoid false-positive indel variants, we filtered out variants with tumor alterative reads less than 6. All somatic variants were annotated by ANNOVAR [15]. The variants that passed through the steps were called ‘processed WES data’ (Additional file 1: Figure S1).

### Analysis of copy number variations (CNVs) by OncoScan™

We used the 330-k OncoScan™ FFPE platform (Affymetrix, Santa Clara, CA, USA) to identify candidate CNVs (amplification/deletion and loss-of-heterozygosity (LOH)). AYA02 was excluded because the amount of DNA was insufficient for OncoScan™. A minimum of 80 ng of DNA from each sample was used for the OncoScan™ platform. The Nexus Express (Affymetrix) software was used to analyze the data and find CNVs. We filtered out CNVs with a CN ≤ 2.5, which were considered insignificant amplification, and analyzed chromosome-level CNVs and focal level CNVs.

### Analysis of CNVs by VarScan2 for AYA02

To analyze CNVs in AYA02, we used VarScan2 as an alternative method to OncoScan™. After processing data up to the ‘Realignment/Recalibration’ step in WES processing (Additional file 1: Figure S1), we processed data based on the manufacturer’s recommendations. We generated mpileup data from recalibrated BAM files of both tumor and normal using SAMtools and used the ‘Copy caller’ module of VarScan2 to generate the relative copy number change (C), which was determined as follows: *C = log*_*2*_*((D*_*T*_*/D*_*N*_)*(I_*N*_*/I*_*T*_*)),* where ‘D’ stands for the average depth, ‘I’ for the number of uniquely mapped bases, ‘N’ for normal, and ‘T’ for tumor [16, 17]. After filtering out mapping quality values <15, we adjusted the relative copy number using the re-centering option in ‘Copy caller’ and segmented copy number regions based on the circular binary segmentation algorithm. After merging, the results were represented by IGV (Integrative Genomics Viewer) [18]. Because the VarScan2 results covered only exon regions, not all genome regions, we analyzed only chromosome-level CNVs that were similar to those obtained with OncoScan™ and did not analyze focal-level CNVs. To create graphs of relative copy number changes, we used data from re-centered relative copy number changes displayed on a log_2_ scale.

### Analysis of mutation frequency and mutation spectrum

The mutation frequency was analyzed by counting the number of variants annotated by ANNOVAR from WES data as nonsynonymous SNVs, synonymous SNVs, nonsense mutations, stop-loss mutations, splicing mutations, frameshift insertions/deletions (indels), in-frame indels, and noncoding RNA in exonic regions. These mutations had previously been described in published data from 12 major cancer studies [19]. To analyze the mutation spectrum, we used SNVs processed with MuTect in all sequenced regions not limited to coding regions.

### Pathway-drug analysis

After assigning the levels to the variants by pattern-based heuristic annotation, we investigated the biologic pathways of variants using DAVID or the literature [20]. To analyze the druggability of the variants, we concentrated mainly on level-1 (strong) variants using DGIdb [21].

### Fusion analysis

We analyzed WTS data from four samples (AYA01, 02, 09, and 10) to identify cancer driving fusions using three different fusion tools, FusionMap, deFuse and ChimeraScan [22–24]. From the results, we selected candidate cancer-driving fusions using a fusion gene list archived in COSMIC (download date: 2015-03-03).

## Results

### AYA cancer samples, platforms and generation of data from WES

Samples analyzed in this study were collected from AYA patients (median age = 32) who had metastatic tumors. Seven different tumor samples were obtained in three different forms (fresh-frozen tissues, FFPE and pleurisy) and analyzed using three different genomics platforms (WES, WTS and OncoScan™) (Table 1).

**Table 1:** Sample information of AYAs cancer patients

No. AYA^a^	Age	Sex	Tumor type	Tissue type	Previous treatment^b^	Platform		
						WES	WTS	OncoScan
#01	30	M	Prostate cancer	Fresh-frozen	Docetaxel + Pd	v	v	v
#02	30	M	Olfactory neuroblastoma	Fresh-frozen	ICE	v	v	-
					Op			
					PORT			
#04	33	M	Head and neck squamous cell carcinoma	Fresh-frozen	Op	v	-	v
					CCRT			
					DP			
					Cetuximab			
					FP			
					Op			
#06	32	M	Urachalcarcinoma	FFPE	Op	v	-	v
#07	21	M	Germ cell tumor	Fresh-frozen	Op	v	-	v
					BEP			
					IE			
#09	34	M	Lung cancer	Pleurisy	-	-	v	-
#10	33	M	Liposarcoma	Fresh-frozen	Op	v	v	v

^a^#03: exclusion, because of no tumor sample provided; #05, 08: exclusion, because of insufficient sample to sequencing

^b^ *Pd* prednisolone, *ICE* ifosfamide + carboplatin + etoposide, *Op* operation, *PORT* postoperative radiotherapy, *CCRT* concurrent chemoradiotherapy, *DP* docetaxel + cisplatin, *FP* 5-fluorouracil + cisplatin, *BEP* bleomycin + etoposide + cisplatin, *IE* ifosfamide + etoposide

We processed WES data using well-known bioinformatics tools described in Supporting Fig. 1 following previously published studies [8, 10]. From the WES data of six tumors and matched normal blood, we generated total of 95 gigabases (Gb, range 12.5–20.0/sample) and 106 Gb (range 12.7–27.0/sample) of mapped sequences, respectively. The mean target coverage was 135X (range, 101X-190X) and 150X (range, 115X-205X) for tumor samples and normal blood, respectively. The coverage of mean target bases exceeded 30X for 89.8 and 92.5 % of the tumor and normal blood samples, respectively (Additional file 2: Table S1).

**Fig. 1:**
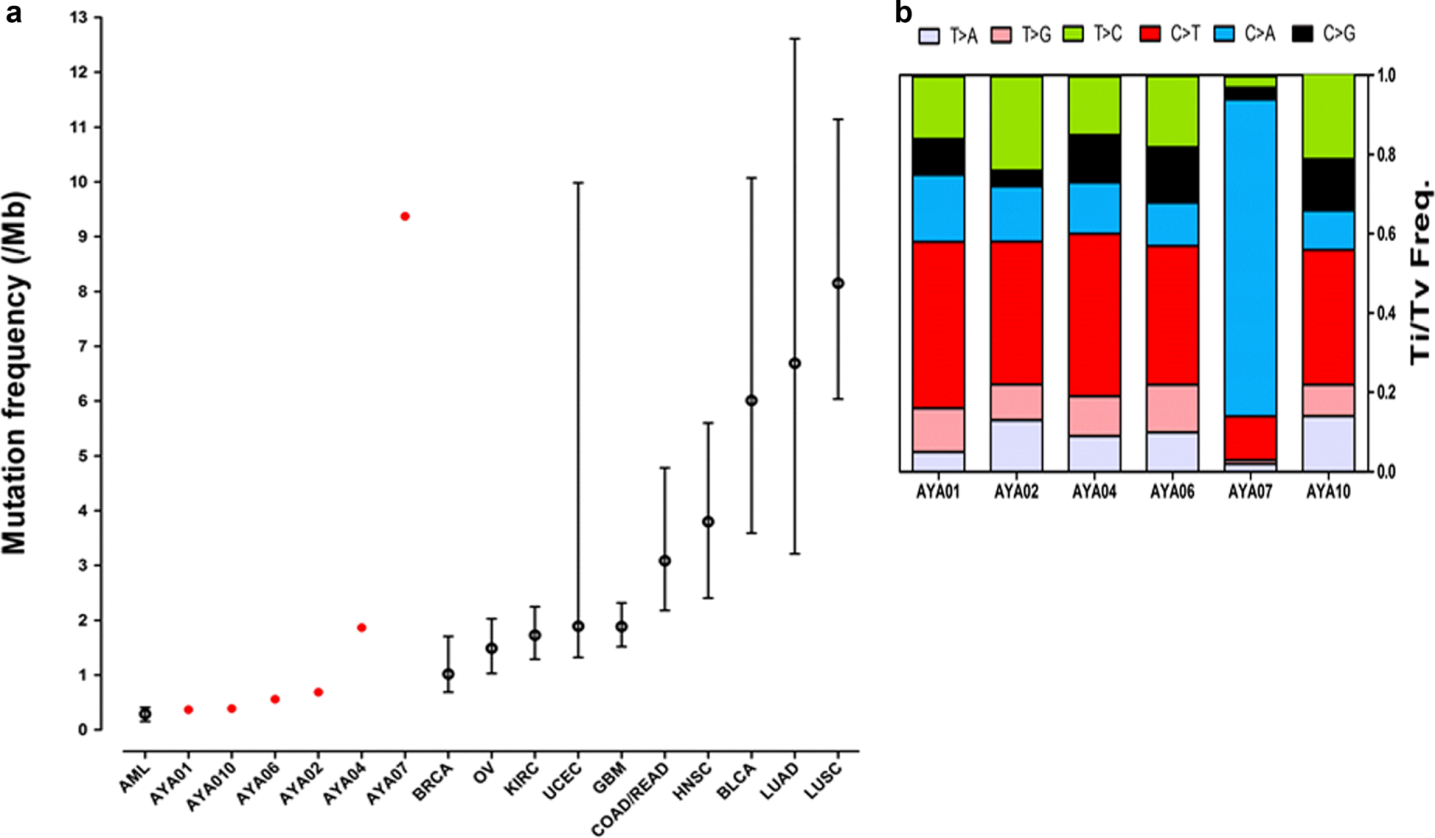
Mutation frequency and mutation spectrum of AYA cancers. **a** Somatic mutation frequencies of pediatric, AYA and adult cancers are shown. Mutation frequencies of AYA cancers were assessed using somatic mutations annotated as nonsynonymous SNVs, synonymous SNVs, nonsense mutations, stop-loss mutations, splicing mutations, frameshift indels, in-frame indels, and noncoding RNA. Mutation frequencies for other cancers were derived from published data from the same regions [19]. **b** The mutation spectrum of AYA cancers (transition and transversion frequency) was assessed using SNVs processed with MuTect in whole-exome regions

### Mutation frequency and mutation spectrum

We analyzed the mutation frequency and mutation spectrum of six samples from processed WES data (Fig. 1). Except for AYA07, which contained a hypermutation, the coding regions (exon and splicing regions) of a total of 276 somatic mutations (median, 40; range, 26–133) were detected (Additional file 3: Table S2). Compared with the mutation frequency of 12 major cancer types obtained from published data, the somatic mutation frequency of most samples was low (median, 0.56/Mb; range, 0.37–1.87), except for AYA07 (Fig. 1a) [19]. This finding is consistent with data showing that somatic mutations are accumulated with age [1]. Although the mutation frequency of AYA07 was high (9.37/Mb), the overall mutation frequency of AYA cancers is low: a recent large-scale study of testicular germ cell tumors (TGCTs), the same tumor type as that of AYA07, demonstrated a low mutation frequency (mean 0.5/Mb) [25]. Additionally, three AYA cancers with higher mutation frequencies (AYA02, 04, and 07) harbored mutations in *TP53* or in several DNA repair genes compared with other samples (mean, 3.98/Mb vs. 0.44/Mb, respectively), as shown in large studies [19].

Except for AYA07, the mutation spectrum of AYA cancers showed prominent C > T transitions, as has been demonstrated in many solid cancers (Fig. 1b) [26]. Interestingly, AYA07 showed a high proportion of C > A transversions (79.7 %). This result may be related to an over-representation of C > A transversions in the TCGT study [25] or multiple cycles of chemotherapy for the AYA07 patient, because increased C > A transversions were observed in all samples obtained from eight relapsed AML patients after chemotherapy [27].

### Individual AYA cancer analysis: patient-specific genetic alterations

After processing the WES data, we annotated variants using ANNOVAR as described in Additional file 1: Figure S1. All annotated variants are described in Additional file 4: Table S3. We then selected driving genetic alterations using our pattern-based annotation, because it is limited to analyzing rare types of cancers using the same statistical methods to select driving genetic alterations used in large-scale studies (Additional file 3: Figure S2) [4]. By applying our approach to TCGA AML data, we could detect all candidate genes that were previously defined using statistical methods (Additional file 3: Figure S3 and Additional file 5: Table S4) [28].

**Fig. 2:**
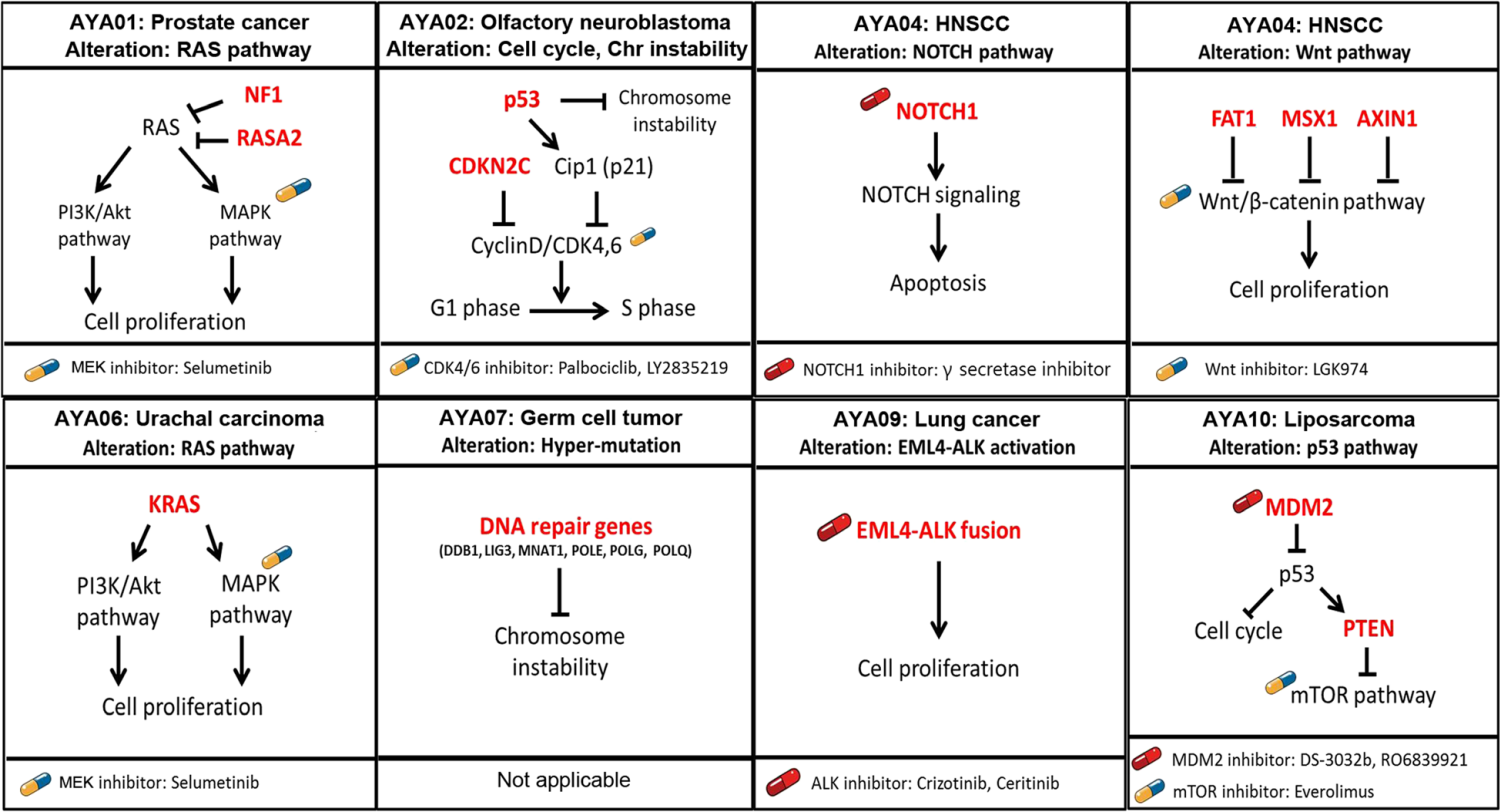
Candidate driving genetic alterations and their druggability in AYA cancers. An analysis of WES/WTS and OncoScan™ with our heuristic annotation identified level-1 candidate genetic alterations. By analyzing DAVID and DGIdb, the representative pathway of AYA cancers and druggability were also identified. The druggability is indicated by illustrations of pills; red indicates a direct inhibitor of a candidate target gene, and blue/yellow indicates an inhibitor of a pathway that includes the candidate alterations. AYA07 was excluded from the candidate gene search due to the hypermutation. All candidate genetic alterations are described in Additional file 4: Table S3

**Fig. 3:**
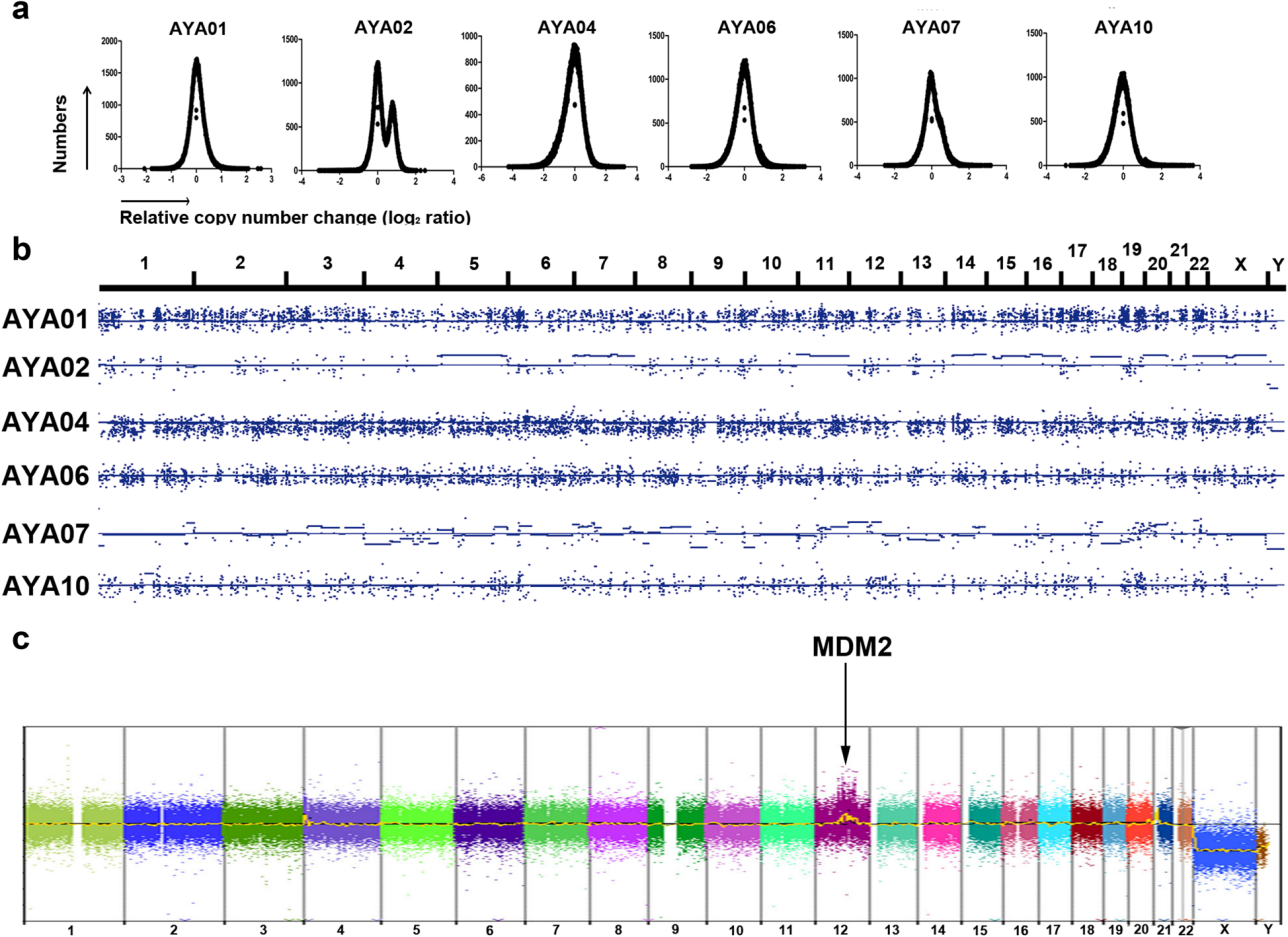
Analysis of CNVs in AYA cancers. **a** Distributions of relative copy number change (*C*) in AYA cancers, shown on a log_2_ scale. **b** Chromosome-level alterations are shown and were processed by VarScan2. Similar patterns were detected by OncoScan™ (Additional file 1: Figure S3). **c** OncoScan™ identified a focal amplification of *MDM2* in AYA10

Except for the hypermutations in AYA07, we focused on level-1 variants to identify driving genetic alterations of AYA cancers that are specific to each patient and may be druggable (Fig. 2 and Additional file 6: Table S5). CNVs were analyzed to identify candidate driving CNVs (OncoScan™) and chromosome-level CNVs (OncoScan™ or VarScan2) (Fig. 3 and Additional file 1: Figure S4).

#### AYA01, prostate cancer: aberrant activation of the RAS pathway

AYA01 showed concurrent loss-of-function in genes of the RasGAP family (*NF1* and *RASA2*). A frameshift deletion and LOH were detected in *NF1*, and a frameshift insertion and splicing mutation were detected in *RASA2* that were validated by sequencing (Additional file 1: Figure S5). Interestingly, concurrent mutations in RasGAPs have been identified in several types of cancers according to the cBio portal (Additional file 1: Figure S6 and Additional file 7: Table S6). Furthermore, a recent study demonstrated the synergistic oncogenic effects of non-canonical Ras mutations in the context of loss-of-function in RasGAP [29]. Because RasGAPs contribute to tumorigenesis, we suggested an MEK inhibitor (as a single agent or in combination) for the treatment of AYA01 [30, 31].

#### AYA02, olfactory neuroblastoma: chromosome instability and loss-of-function of CDKN2C

AYA02 harbored a chromosome-level alteration with a *TP53* missense mutation that contributed to chromosome instability [32]. Interestingly, AYA02 showed a double peak of a relative copy number change and arm-level alterations, which differed from other tumors (Fig. 3a and b). A loss-of-function in *CDKN2C* was identified with high-allelic frequency (0.667). Given the tumor suppressor function of *CDKN2C* in breast cancer, a loss-of-function of *CDKN2C* may have driven tumor formation in AYA02; therefore, we selected CDK4/6 inhibitors as a potential drug (palbociclib and LY2835219) [33].

#### AYA04, HNSCC: alteration of the Wnt and NOTCH pathways

AYA04 harbored *TP53* mutations with alterations in *FAT1*, *NOTCH1* and *SMAD4* that have been recurrently discovered by several large-scale studies of head and neck cancer [34–36]. Specifically, AYA04 harbored concurrent mutations in Wnt pathway genes, such as *FAT1*, *MSX1* and *AXIN1*, which were reported in a recent large-scale study of HNSCC with HPV (−) [34]. We suggested potential drugs (LGK974 and γ secretase inhibitor) for AYA04 based on the importance of the Wnt and NOTCH pathways.

#### AYA06, urachal carcinoma: alteration in noted KRAS mutation

Because AYA06 showed only one level-1 variant in *KRAS* (G13D) with no candidate CNVs, we selected an MEK inhibitor (selumetinib) as a potential drug. However, a missense mutation in *USP6* (R133K, Lv2 OG) was detected in AYA06 and AYA04. USP6 is known to be able to initiate tumorigenesis either in cell lines or in mice via the activation of the NF-κB pathway, although the function of the R133K variant remains elusive [37].

#### AYA07, germ cell tumor: alteration in DNA repair genes and genome instability

AYA07 was excluded from the identification of candidate driving genetic alterations, because AYA07 showed a high mutation frequency with many CNVs may be caused by missense mutations in six DNA repair genes (*DDB1*, *LIG3*, *MNAT1*, *POLE*, *POLG* and *POLQ*) (Fig. 1 and Additional file 4: Table S3) [38]. The most frequently mutated gene, *KIT*, which was found in a large-scale study of TGCT, was not detected in AYA07 [25].

#### AYA09, lung cancer: well-known fusion, EML4-ALK

AYA09 was analyzed by WTS only because an IHC result for ALK was positive (data not shown). Because the *EML4-ALK* detected in our fusion processing is well known in lung cancer, crizotinib and ceritinib were recommended as potential drugs for AYA09 (Additional file 1: Figure S6 and Additional file 8: Table S7). The patient was treated with crizotinib and showed clinically significant tumor shrinkage, as expected.

#### AYA10, liposarcoma: amplification of MDM2 with PTEN deletion

Although level-1 SNV/indel alterations were not detected, CNVs in *MDM2* and *PTEN,* which play a role in the p53 pathway, were identified in AYA10 (Fig. 3c). Target-specific drugs for *MDM2* amplification, DS-3032b and RO6839921, plus an mTOR inhibitor, everolimus, were recommended.

### Comparison of candidate driving genes from AYA cancers and cancers in other age groups

To investigate differences in the genomic profiles between AYA cancers and cancers found in other age groups, we compared candidate driving genes between AYAs and all age groups for the same cancer based on published data from analyses similar to ours (Table 2). The mutation pattern of AYA01 (prostate cancer) differed from that shown in a large-scale study analysis. Whereas AYA01 harbored alterations in the Ras pathway (*NF1* and *RASA2*), prostate cancer from other age groups showed recurrent mutations in *SPOP*, *TP53*, and *PTEN* [39]. However, several commonly altered genes in HNSCC, such as *TP53*, *FAT1*, and *NOTCH1*, were identified in both AYA04 and in large-scale studies, whereas other mutations differed [34].

**Table 2:** Comparison of candidate driving genes of same cancer type from AYAs with those from all age group

	Prostate cancer		HNSCC	
	AYA01	Barbieri, et al.	AYA04	TCGA
Sequencing platform	WES (*n* = 1)	WES (*n* = 112)	WES (*n* = 1)	WES (*n* = 279)
Exome capture kit	Agilent SureSelect	Agilent SureSelect	Agilent SureSelect	Agilent SureSelect
Sequencing instrument	Illumina HiSeq	Illumina HiSeq	Illumina HiSeq	Illumina HiSeq
Depth (mean)	138X	118X	198X	95X
Age (median)	30	63	33	61
Bioinformatic pipeline				
Alignment	bwa (hg19)			
Deduplication	Picard			
Realignment	GATK			
Recalibration	GATK			
Variant calling				
SNVs	MuTect			
Indels	Somatic Indel Detector/Indelocator			
Selection of candidate SNV/Indels	Heuristic annotation	MutSig	Heuristic annotation	MutSig
CNVs detection	AffymetrixOncoScan	Affymetrix SNP 6.0	AffymetrixOncoScan	Affymetrix SNP 6.0
Selection of candidate CNVs	Heuristic annotation	GISTIC	Heuristic annotation	GISTIC
Candidate driving genes				
SNVs/Indels	NF1	SPOP	TP53	CDKN2A
	RASA2	TP53	FAT1	FAT1
	ATAD5	PTEN	MSX1	TP53
		FOXA1	USP6	CASP8
		CDKN1B	ANK2	AJUBA
		ZNF595	CHD5	PIK3CA
		THSD7B	FOXL2	NOTCH1
		MED12	ITGB4	KMT2D
		NIPA2	LECT1	NSD1
		PIK3CA		HLA-A
		C14orf9		TGFBR2
		SCN11A		
CNVs^a^	NF1	NA^b^	NOTCH1	FADD
	SUZ12		SMAD4	CDKN2A
			SETD2	CSMD1
			PTCH1	SOX2
			AXIN1	LRP1B
			BAP1	EGFR
			CDH1	FAT1

^a^Emphasized results from published paper (q < 0.0001)

^b^There was not available emphasized result of CNVs

## Discussion

### Genomic profiling of AYA cancers

In this study, we described the genomic profiles of seven different rare types of AYA cancers using three different genomics platforms (WES, WTS and OncoScan™). After processing genomics data, we identified potential druggable targets for each cancer and selected existing anti-cancer drugs to treat individual patients (Fig. 2 and Additional file 6: Table S5).

We identified candidate driving genetic alterations specific to each patient using logical manual curation (pattern-based heuristic annotation) as alternative to statistical method (Additional file 9: Supplementary materials and methods and Additional file 10: Table S8). It is needed to alternative method to select candidate genes in rare type of cancers, like AYA cancers, since low number of samples is limited to the selection of candidate driving genes using statistical method as shown in large-scale studies [4].

Because the features of AYA cancers are distinct from those of other age groups, including the incidences and clinical outcomes, studying the genomic profiles of AYA cancers is important to identify the unique features of AYA cancers [5, 6]. When comparing candidate genes between AYAs and all age groups for the same cancer type, we identified different candidate genes in prostate cancer (AYA01) and a germ cell tumor (AYA07), although several common candidate genes (*TP53*, *FAT1*,and *NOTCH1*) were found in HNSCC (AYA04) in both AYAs and all age groups (Table 2). These results showed that AYA-specific genetic alterations may be different from those in other age groups; thus, further study is needed to define the significance of the differences in the genetic alterations between AYAs and other age groups.

### Clinical implication

In this study, we analyzed individual cancer genomes and suggested potential drugs for each patient based on his or her genetic alterations. Characterizing the genomes of patients and genomics-driven knowledge enabled personalized medicine and advanced cancer genomics for clinical implications [40]. Moreover, to establish the clinical validity of genetic tests, especially for NGS data, the FDA discussed ‘post-marketing pursuit’ to define the clinical implications of variants generated from NGS, which have remained unknown [41]. Therefore, we expect many prospective genomic studies, such as our study, to link the patient to therapy as well as diagnosis, prognosis, and monitoring [42].

## Conclusion

We analyzed seven different metastatic AYA cancers’ genome, and potential targets were identified. Genetic alterations in cancers from AYA and those from all age groups were varied by their cancer type.

## Additional files

Additional file 1: **Figure S1.** WES pipeline for our study. **Figure S2.** Pattern-based heuristic annotation to identify driving genetic alterations. **Figure S3.** Pattern-based heuristic annotation for large-scale samples. **Figure S4.** Chromosome-level CNVs of AYA cancers from OncoScan™ and VarScan2. **Figure S5.** Sequencing validation of RASA2 and NF1 in AYA01 sample. **Figure S6.** Concurrency of RasGAPs in large-scale studies. **Figure S7.** EML4-ALK validation in AYA09 cells. (DOCX 50738 kb)

Additional file 2: Table S1. Sequencing information for WES data. (PDF 95 kb)

Additional file 3: Table S2. Mutation frequency of WES data for AYA cancers. (PDF 50 kb)

Additional file 4: Table S3. Processed WES data. (PDF 635 kb)

Additional file 5: Table S4. Patient-specific genetic alterations of TCGA AML study selected by our pattern-based annotation. (PDF 156 kb)

Additional file 6: Table S5. Candidate driving genetic alterations of AYA cancers. (PDF 150 kb)

Additional file 7: Table S6. RasGAPs in large-scale studies. (PDF 234 kb)

Additional file 8: Table S7. EML4-ALK fusion in AYA09. (PDF 128 kb)

Additional file 9: Supplementary materials and methods. (DOC 21 kb)

Additional file 10: Table S8. CVE list. (PDF 285 kb)

## Abbreviation

AYA: adolescent and young adult
CNV: copy number variation
FFPE: formalin-fixed paraffin-embedded
GATK: the genome analysis toolkit
HNSCC: head and neck squamous cell carcinoma
ICGC: international cancer genome consortium
IGV: integrative genomics viewer
LM-PCR: ligation-mediated PCR
NGS: next generation sequencing
SNV: single nucleotide variant
TCGA: the cancer genome atlas
TGCT: testicular germ cell tumor
WES: whole exome sequencing
WTS: whole transcriptome sequencing
