# Supplementary material for: Clinical application of genomic profiling to find druggable targets for adolescent and young adult (AYA) cancer patients with metastasis
Source: BMC Cancer. 2016 Feb 29;16:170. doi: 10.1186/s12885-016-2209-1 (PMC4772349; doi:10.1186/s12885-016-2209-1)
Supplement: Supplementary file 1 — Figure S1. WES pipeline for our study. Figure S2. Pattern-based heuristic annotation to identify driving genetic alterations. Figure S3. Pattern-based heuristic annotation for large-scale samples. Figure S4. Chromosome-level CNVs of AYA cancers from OncoScan™ and VarScan2. Figure S5. Sequencing validation of RASA2 and NF1 in AYA01 sample. Figure S6. Concurrency of RasGAPs in large-scale studies. Figure S7. EML4-ALK validation in AYA09 cells. (DOCX 50738 kb) [file 12885_2016_2209_MOESM1_ESM.docx]

**Supplementary Figures**


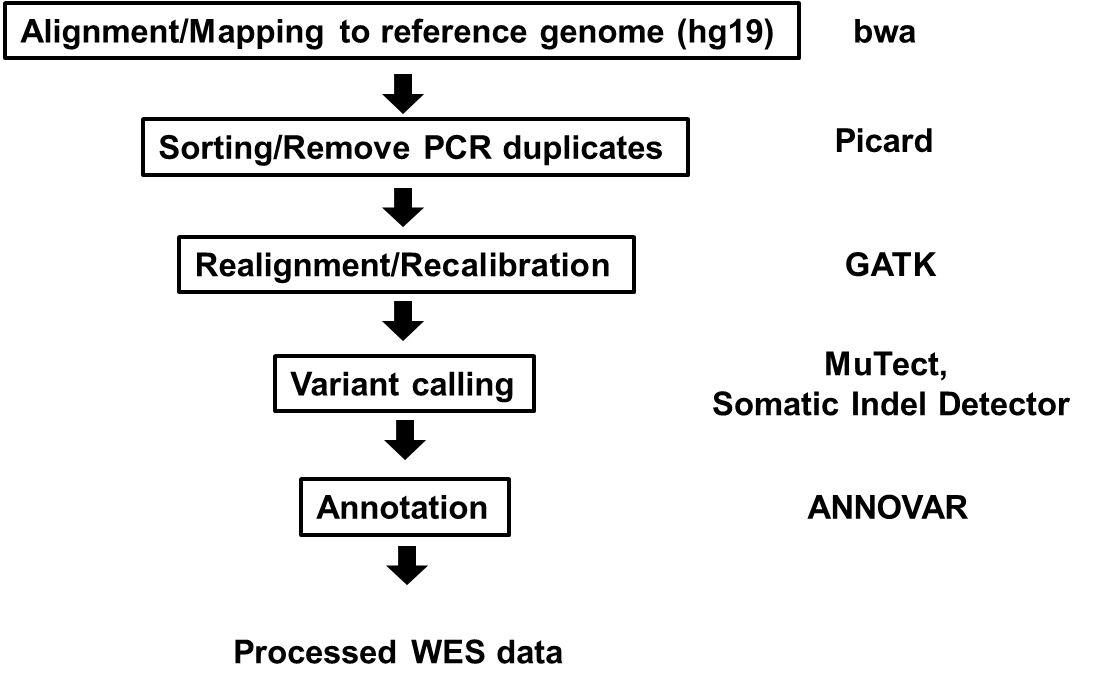


Figure S1. WES pipeline for our study. Whole exome sequencing data were processed by this pipeline comprised of well-known bioinformatics tools.


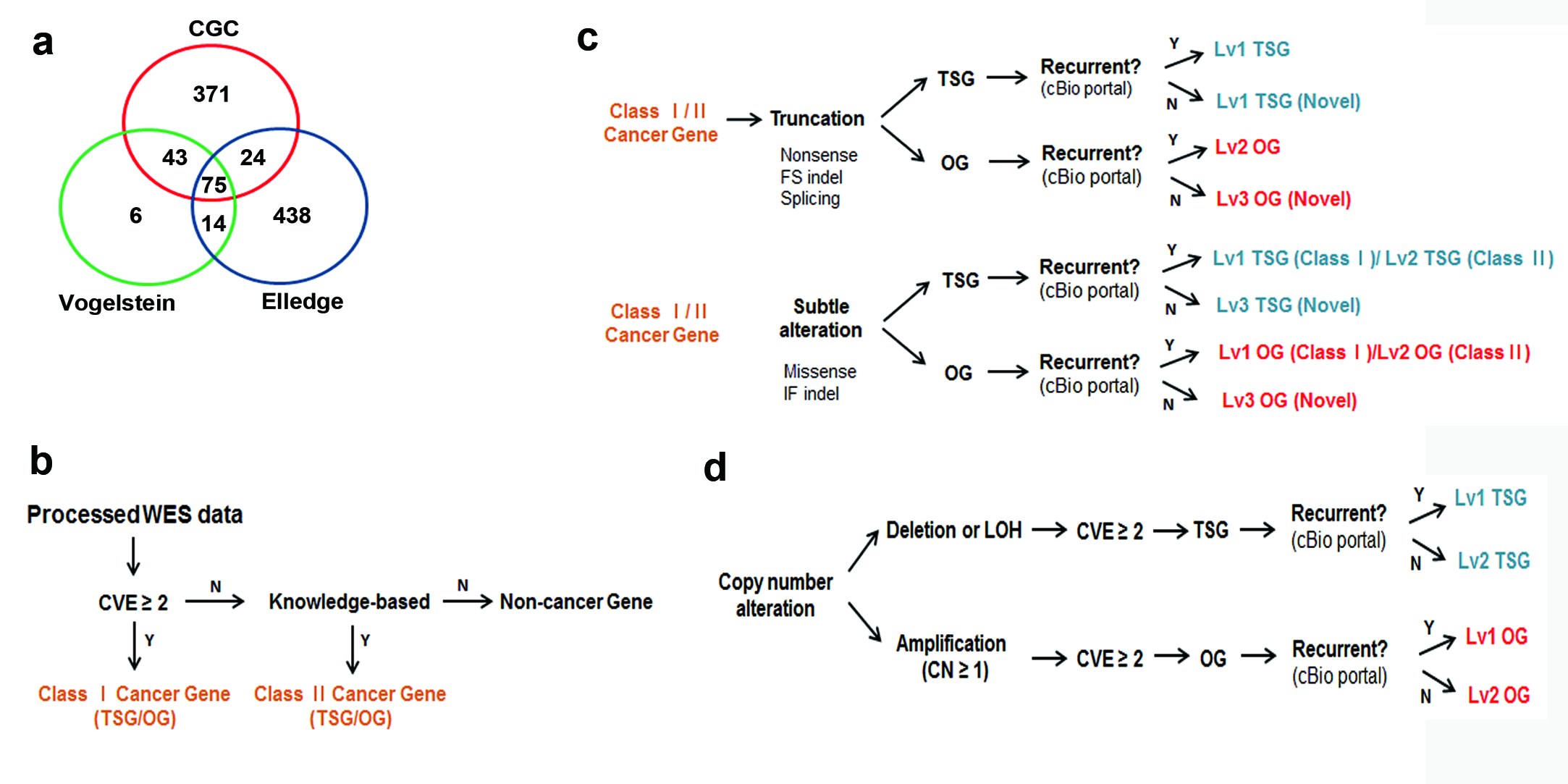


**Figure S2. Pattern-based heuristic annotation to identify driving genetic alterations. (a)** Three of well-known cancer gene lists were represented, ‘CVE’ list (C for cancer gene census, V for cancer gene list from Vogelstein group, and E for cancer gene list from Elledge group [1-3]. Details were described in Supplementary materials and methods. **(b)** Detection of cancer gene from our sequencing results was shown. Class I cancer gene indicated well-known cancer gene, and Class II cancer gene indicated less-known cancer gene. **(c)** Class I/II cancer genes were questioned by how much they contribute to tumorigenesis considering their mutation type (truncation, subtle alteration), functional characteristics (TSG/OG) and recurrence of the mutations. Finally, they were assigned to levels. **(d)** Copy number alteration was analyzed by heuristic way similar to WES data considered mutation type, CVE score, functional characteristics and recurrence of the alterations. Recurrent copy number alterations were determined by comparing the results of copy number variation from various cancer types as described in ref. [4]


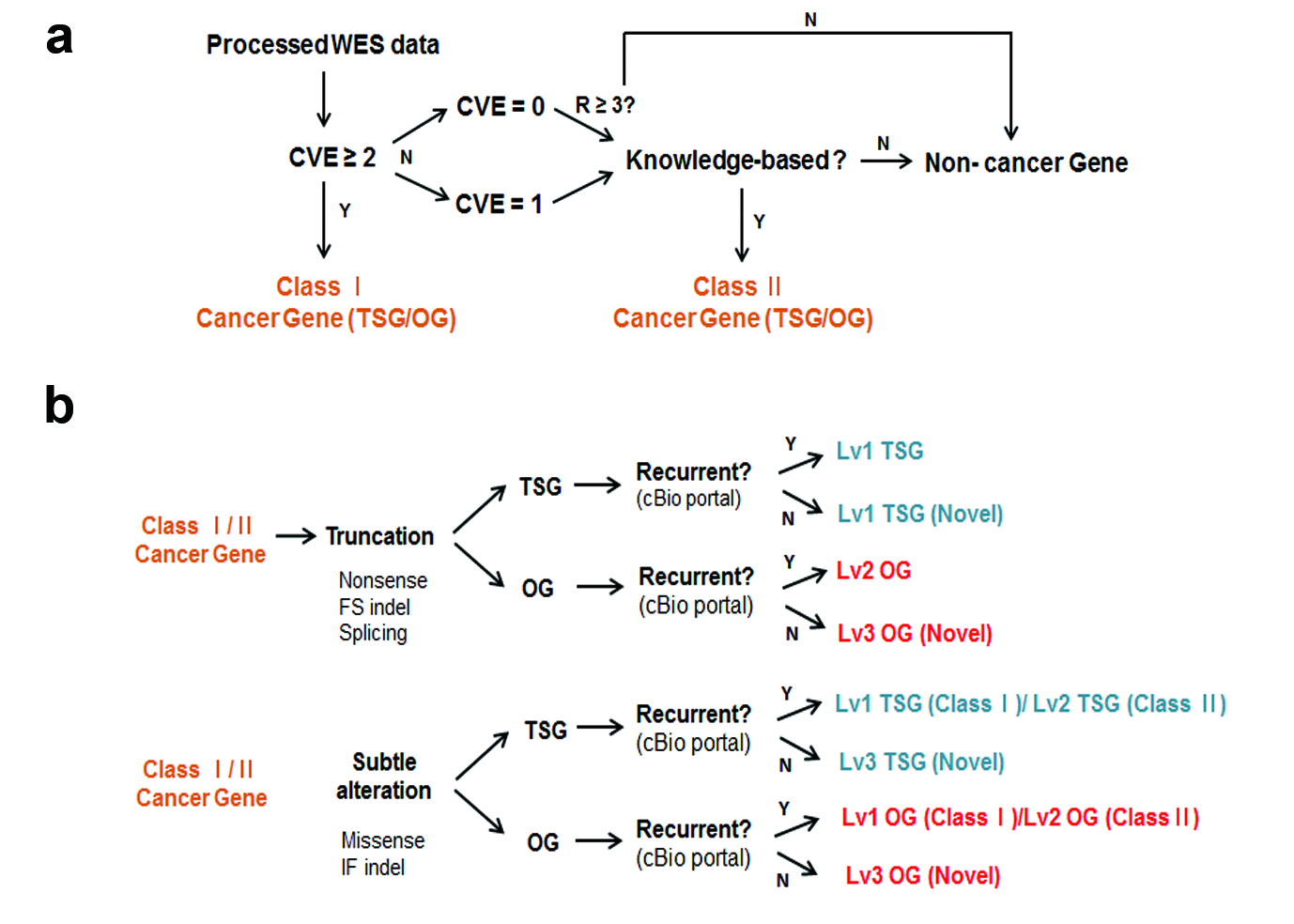


Figure S3. Pattern-based heuristic annotation for large-scale samples. It was similar with the scheme of pattern-based heuristic annotation for single sample (Figure S2), except for the consideration of recurrent variant between samples (R) represented in (a).


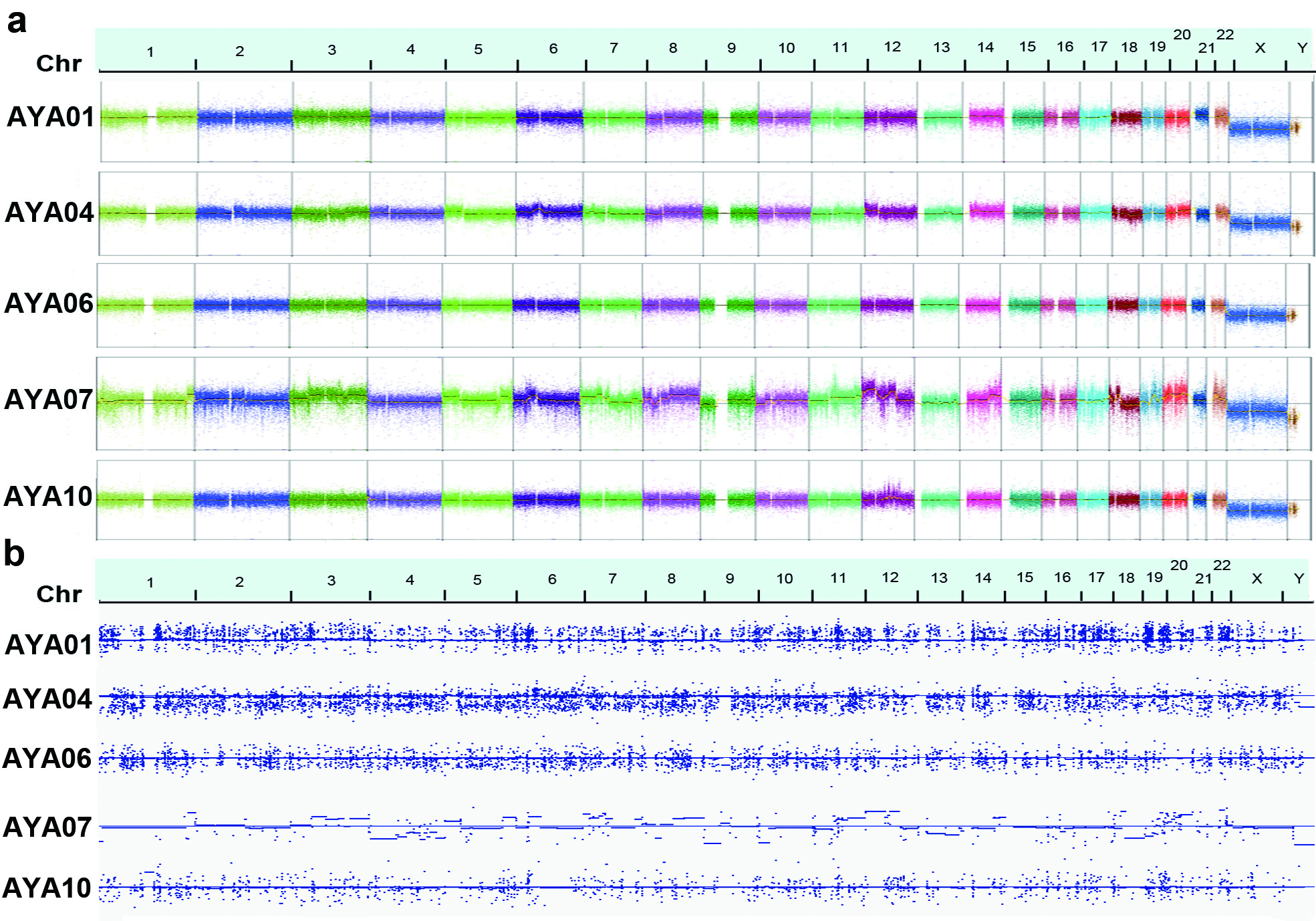


**Figure S4. Chromosome-level CNVs of AYA cancers from OncoScan^TM^ and VarScan2.** Since whole exome sequencing generated data from only exome regions (~2% of whole genome), processed data from VarScan2 were limited to detect candidate focal CNVs. However, this figure validated the feasibility of VarScan2 to analysis of chromosome-level CNVs due to similar patterns were shown between data from OncoScan^TM^ platform **(a)** and VarScan2 **(b)**.


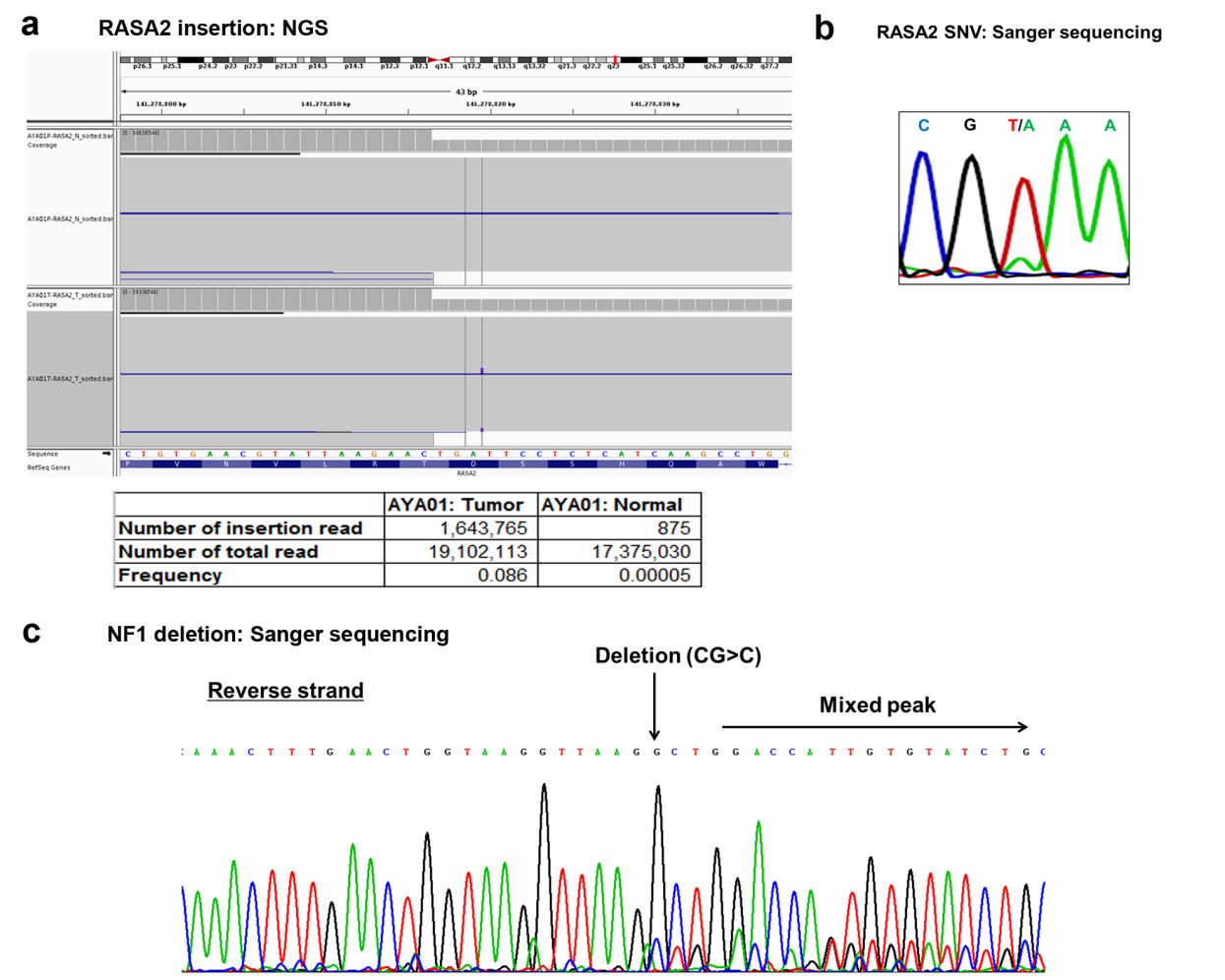


**Figure S5. Sequencing validation of RASA2 and NF1 in AYA01 sample.** Representative potential candidate drivers of AYA01 were validated by NGS or Sanger sequencing. (**a**) Insertion of RASA2 was validated by NGS (Illumina Hiseq2000) with very deep depth (>10,000,000X) since allelic frequency of the mutation was under 0.1. (**b**) Splicing mutation of RASA2 was validated by Sanger sequencing. (**c**) Deletion of NF1 was validated by Sanger sequencing.


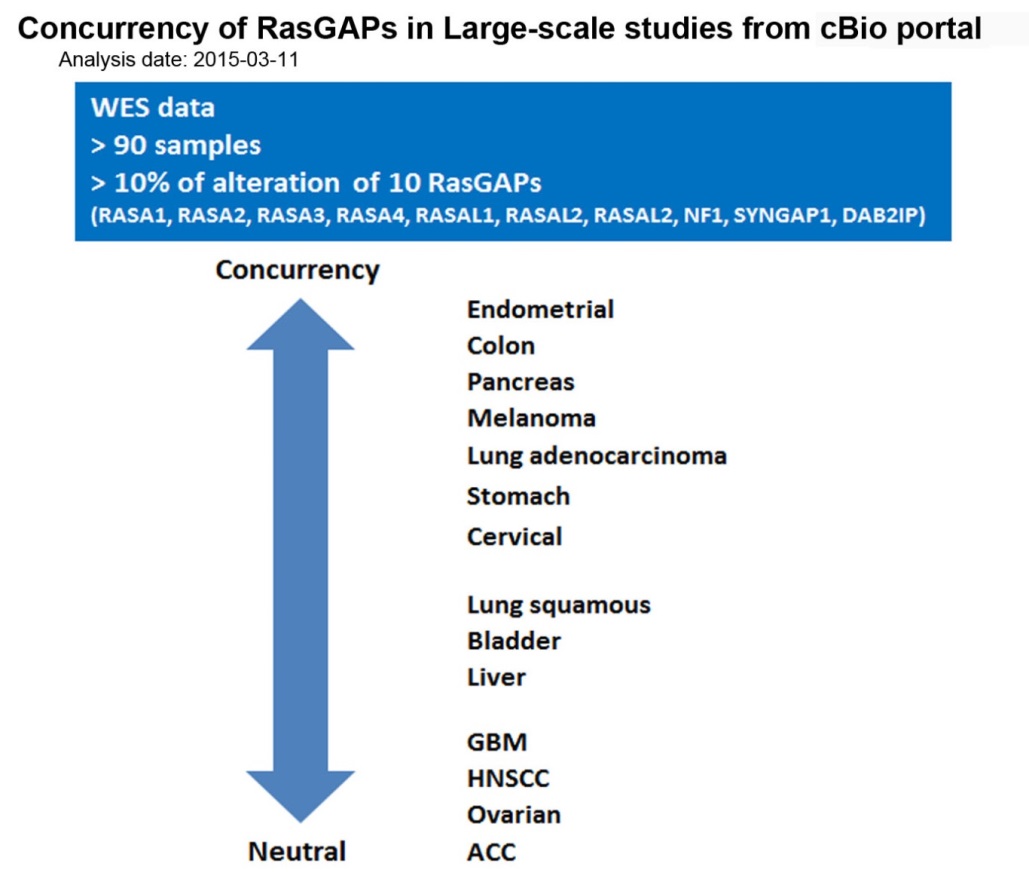


Figure S6. Concurrency of RasGAPs in large-scale studies. Ten of RasGAPs [5] were analyzed to their tendency of alteration whether they were altered together or mutually exclusively. WES data of 14 cancer types were selected in this analysis which included more than 90 samples and at least 10% alteration of 10 RasGAPs. Details were described in supplementary table 6.


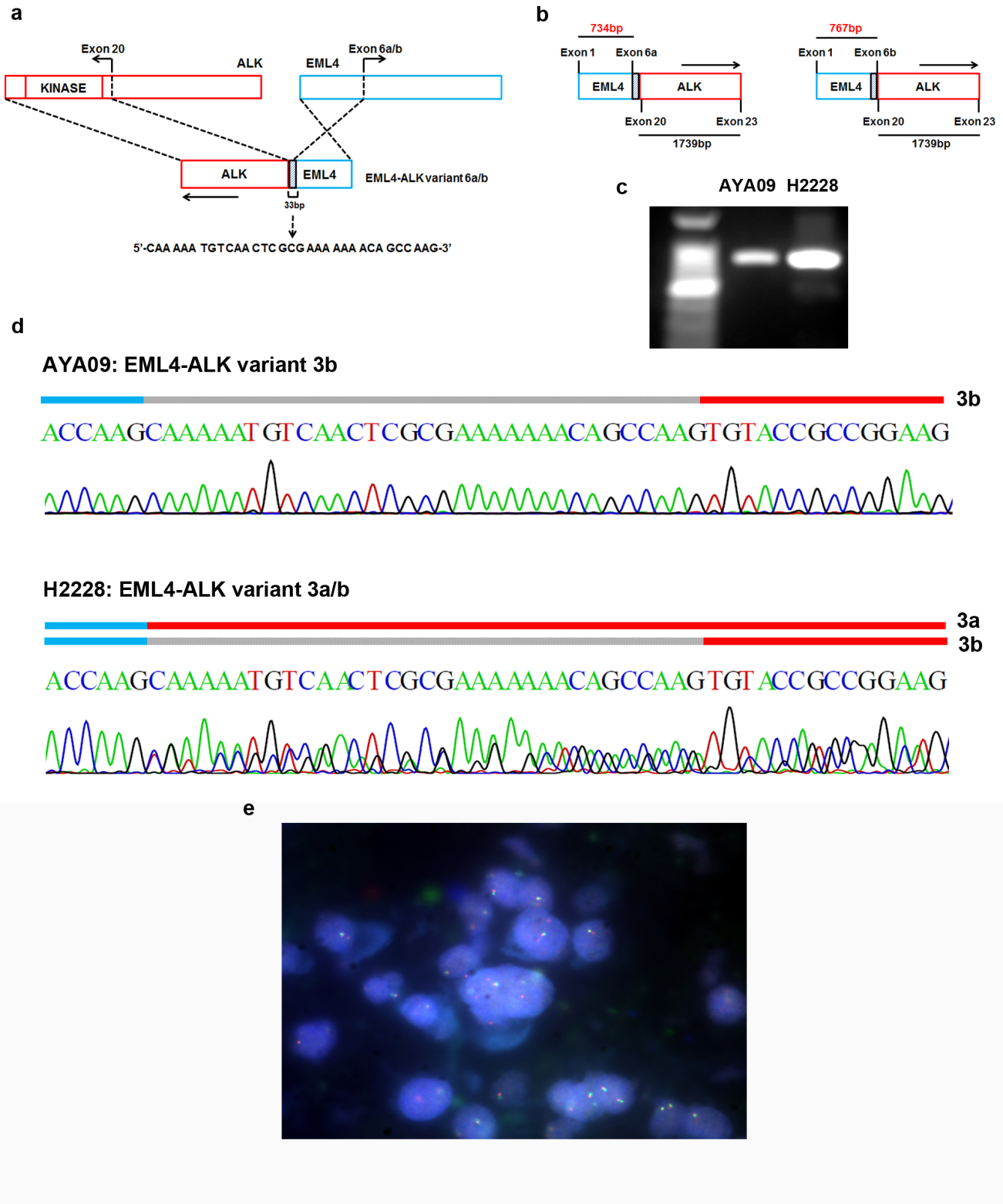


**Figure S7. EML4-ALK validation in AYA09 cells.** **(a)** Process of EML4-ALK fusion (variant 3) was shown. The fusion was composed of exon 1-6 of EML4 and exon 20-28 of ALK. Variant 3 of EML4-ALK was sub-divided into two formats (3a and 3b) that are different sequence of 33 bp as represented. **(b)** Variant 3a of EML4-ALK (left) and variant 3b of EML4-ALK (right) were shown that had common ALK sequence. **(c)** RT-PCR result of variant 3 of EML-ALK was shown. RNA was extracted from cells of AYA09 and H2228 (which was cell line known to having variant 3a/b of EML4-ALK). **(d)** Sequence of the result of RT-PCR was identified by Sanger sequencing. AYA09 showed only variant 3b of EML4-ALK, but H2228 showed mixture of variant 3a and 3b of EML4-ALK. Color bar represented origin of the sequence from EML4 (blue), different sequence between variant 3a and 3b (grey) or ALK (red). (**e**) EML4-ALK fusion was detected by using break-apart fluorescent in situ hydridyzation (FISH). Positive signals were defined as split signals ≥ 15%.

**References**

1. Futreal PA, Coin L, Marshall M, Down T, Hubbard T, Wooster R et al. A census of human cancer genes. Nature reviews Cancer. 2004;4(3):177-83. doi:10.1038/nrc1299.

2. Vogelstein B, Papadopoulos N, Velculescu VE, Zhou S, Diaz LA, Jr., Kinzler KW. Cancer genome landscapes. Science. 2013;339(6127):1546-58. doi:10.1126/science.1235122.

3. Davoli T, Xu AW, Mengwasser KE, Sack LM, Yoon JC, Park PJ et al. Cumulative haploinsufficiency and triplosensitivity drive aneuploidy patterns and shape the cancer genome. Cell. 2013;155(4):948-62. doi:10.1016/j.cell.2013.10.011.

4. Zack TI, Schumacher SE, Carter SL, Cherniack AD, Saksena G, Tabak B et al. Pan-cancer patterns of somatic copy number alteration. Nature genetics. 2013;45(10):1134-40. doi:10.1038/ng.2760.

5. King PD, Lubeck BA, Lapinski PE. Nonredundant functions for Ras GTPase-activating proteins in tissue homeostasis. Science signaling. 2013;6(264):re1. doi:10.1126/scisignal.2003669.
