# Supplementary material for: Clinical application of genomic profiling to find druggable targets for adolescent and young adult (AYA) cancer patients with metastasis
Source: BMC Cancer. 2016 Feb 29;16:170. doi: 10.1186/s12885-016-2209-1 (PMC4772349; doi:10.1186/s12885-016-2209-1)
Supplement: Supplementary file 2 — Sequencing information for WES data. (PDF 95 kb) [file 12885_2016_2209_MOESM2_ESM.pdf]

Table S1. Sequencing information for WES data

Sample	AYA01_Normal	AYA01_Tumor	AYA02_Normal	AYA02_Tumor	AYA04_Normal	AYA04_Tumor
Target Regions	199,259	199,259	199,259	199,259	199,259	199,259
Bases in Target Region	70,369,848	70,369,848	70,369,848	70,369,848	70,369,848	70,369,848
Bases Sequenced	14,340,725,784	13,394,620,000	17,853,383,170	15,593,444,136	26,970,195,236	19,997,019,694
Mean Target Coverage	145.01	129.82	118.97	103.26	205.08	190.36
% of targets at coverage 30X	92.61	91.91	90.29	87.03	96.04	91.00

Sample	AYA06_Normal	AYA06_Tumor	AYA07_Normal	AYA07_Tumor	AYA10_Normal	AYA10_Tumor
Target Regions	199,259	199,259	199,259	199,259	199,259	199,259
Bases in Target Region	70,369,848	70,369,848	70,369,848	70,369,848	70,369,848	70,369,848
Bases Sequenced	20,503,746,592	19,863,262,162	13,348,069,706	13,796,972,488	12,669,135,182	12,476,740,282
Mean Target Coverage	194.76	164.54	119.20	121.96	115.38	101.3
% of targets at coverage 30X	95.55	93.93	89.44	88.84	90.8	86.03

	AYAs_Normal	AYAs_Tumor
Bases Sequenced	105,685,255,670	95,122,058,762
Mean Target Coverage	149.73	135.21
% of targets at coverage 30X (mean)	92.46	89.79
