# Supplementary material for: Clinical application of genomic profiling to find druggable targets for adolescent and young adult (AYA) cancer patients with metastasis
Source: BMC Cancer. 2016 Feb 29;16:170. doi: 10.1186/s12885-016-2209-1 (PMC4772349; doi:10.1186/s12885-016-2209-1)
Supplement: Supplementary file 3 — Mutation frequency of WES data for AYA cancers. (PDF 50 kb) [file 12885_2016_2209_MOESM3_ESM.pdf]

Table S2. Mutation frequency of WES data for AYA cancers

	AYA01	AYA02	AYA04	AYA06	AYA07	AYA10
**Somatic mutation/Mb**	0.37	0.69	1.87	0.56	9.37	0.39
**Number of somatic mutations**	26	49	133	40	665	28
**Synonymous SNV**	1	8	28	3	157	5
**Nonsynonymous SNV**	10	13	83	15	441	12
**Nonsense**	3	1	6	1	5	0
**Stoploss**	0	0	0	0	2	0
**Splicing mutation**	2	2	1	2	14	1
**ncRNA mutation**	3	8	8	5	34	0
**Indels**	7	17	7	14	12	10
**DNA repair genes /Mutation context***	TDG /ns	TP53 /ns	TP53 /sp	MSH3 /ns	DDB1/ns<br>LIG3/ns<br>MNAT1/ns<br>POLE/ns<br>POLG/ns<br>POLQ/ns	-
**Allele freq. of DNA repair genes**	0.05	0.58	0.28	0.1	0.06 (DDB1)<br>0.06 (LIG3)<br>0.08 (MNAT1)<br>0.06 (POLE)<br>0.06 (POLG)<br>0.27 (POLQ)	-

*ns- nonsynonymous SNV, sp: splicing alteration
