# Supplementary material for: Clinical application of genomic profiling to find druggable targets for adolescent and young adult (AYA) cancer patients with metastasis
Source: BMC Cancer. 2016 Feb 29;16:170. doi: 10.1186/s12885-016-2209-1 (PMC4772349; doi:10.1186/s12885-016-2209-1)
Supplement: Supplementary file 4 — Processed WES data. (PDF 635 kb) [file 12885_2016_2209_MOESM4_ESM.pdf]

Table S3. Processed WES data

(A) AYA01

Chr	Start	End	Ref	Alt	Altered gene	T_Ref	T_Alt	dbSNP138		CVE list			Mutation context**
								All	NonFlagged*	C	V	E	
1	110050210	110050210	G	GC	AMIGO1	106	6	-	-	-	-	-	FS ins
8	131127909	131127909	C	A	ASAP1	85	7	-	-	-	-	-	nonsense
17	29162451	29162451	G	A	ATAD5	71	10	-	-	-	-	-	nsSNV
14	96730408	96730408	C	T	BDKRB1	120	24	-	-	-	-	-	nsSNV
9	135762805	135762805	T	G	C9orf9	12	4	-	-	-	-	-	nsSNV
16	69155012	69155012	G	A	CHTF8	122	8	rs375808540	rs375808540	-	-	-	nsSNV
8	37888131	37888131	G	GC	EIF4EBP1	45	6	-	-	-	-	-	FS ins
22	29630189	29630189	G	T	EMID1	31	3	-	-	-	-	-	splicing
11	68700794	68700794	C	A	IGHMBP2	23	5	-	-	-	-	-	nsSNV
19	46387815	46387815	C	T	IRF2BP1	119	17	-	-	-	-	-	sSNV
1	15386680	15386680	G	GCC	KAZN	248	15	-	-	-	-	-	FS ins
11	65266568	65266568	T	C	MALAT1	41	7	-	-	-	-	-	ncRNA
22	51048683	51048683	G	GC	MAPK8IP2	84	6	-	-	-	-	O	UN
20	29637976	29637976	G	C	MLLT10P1	14	4	-	-	-	-	-	ncRNA
7	100645731	100645731	G	A	MUC12	44	3	-	-	-	-	-	nsSNV
13	77642780	77642780	G	A	MYCBP2	56	5	-	-	-	-	-	nonsense
2	203991403	203991403	C	A	NBEAL1	158	12	-	-	-	-	-	nsSNV
17	29588833	29588834	GC	G	NF1	216	45	-	-	O	O	O	FS del
1	248154493	248154493	T	C	OR2L1P	8	3	-	-	-	-	-	ncRNA
17	7411682	7411682	C	T	POLR2A	142	11	-	-	-	-	-	nsSNV
X	150869103	150869103	C	A	PRRG3	32	11	-	-	-	-	-	nsSNV
3	141327541	141327541	T	A	RASA2	31	3	-	-	-	-	-	splicing
3	141278819	141278819	A	AT	RASA2	108	11	-	-	-	-	-	FS ins
21	33043756	33043756	C	A	SCAF4	97	5	-	-	-	-	-	nonsense
11	64323714	64323714	G	GC	SLC22A11	167	9	-	-	-	-	-	FS ins
22	26879946	26879967	GGAGGCGGCGCCCCGGGGGAGA	G	SRRD	77	25	-	-	-	-	-	IF del
12	104376700	104376700	A	C	TDG	78	4	rs61937630	rs61937630	-	-	-	nsSNV

*NonFlagged: dbSNP files subtracting Flagged dbSNP entries. Flagged SNPs include SNPs < 1% minor allele frequency (MAF) (or unknown), mapping only once to reference assembly, flagged in dbSnp as "clinically associated".

**nsSNV: non-synonymous SNV, sSNV: synonymous SNV, FS ins: frameshift insertion, FS del: frameshift deletion, IF ins: in-frame insertion, IF del: in-frame deletion, ncRNA: non-coding RNA, UN: Unknown. transcript maps to multiple locations, all as "coding transcripts", but none has a complete ORF.

Table S3. Processed WES data
(B) AYA02

Chr	Start	End	Ref	Alt	Altered gene	T_Ref	T_Alt	dbSNP138		CVE list			Mutation context**
								All	NonFlagged*	C	V	E	
7	152513619	152513619	G	A	ACTR3B	71	10	-	-	-	-	-	sSNV
7	134132780	134132783	ATCC	A	AKR1B1	35	37	-	-	-	-	-	IF del
17	79954560	79954560	G	GC	ASPSCR1	114	6	-	-	O	-	-	FS ins
3	10401667	10401667	G	A	ATP2B2	71	4	-	-	-	-	-	sSNV
16	84495698	84495698	G	A	ATP2C2	38	3	-	-	-	-	-	sSNV
1	28564378	28564378	G	GA	ATPIF1	30	72	-	-	-	-	-	FS ins
12	112036746	112036749	GGGC	G	ATXN2	4	7	-	-	-	-	-	IF del
13	70713509	70713509	A	ACTGC	ATXN8OS	4	9	-	-	-	-	-	ncRNA
9	121929604	121929604	G	GTGT	BRINP1	44	61	-	-	-	-	-	IF ins
7	2963980	2963980	G	GT	CARD11	23	18	-	-	O	O	-	FS ins
17	78165189	78165189	G	A	CARD14	41	6	-	-	-	-	-	nsSNV
16	1484849	1484849	G	A	CCDC154	22	20	-	-	-	-	-	nsSNV
22	37964408	37964429	CCAGCGCCTGCT	C	CDC42EP1	16	14	-	-	-	-	-	IF del
1	51436116	51436116	C	T	CDKN2C	10	20	-	-	O	O	-	nonsense
14	105352853	105352853	C	CG	CEP170B	51	6	-	-	-	-	-	FS ins
11	46342080	46342081	GT	G	CREB3L1	111	6	-	-	O	-	-	splicing
11	46321710	46321710	C	T	CREB3L1	47	6	-	-	O	-	-	sSNV
1	225528183	225528183	C	A	DNAH14	132	6	rs3856145	rs3856145	-	-	-	nsSNV
16	3706179	3706179	G	A	DNASE1	155	6	-	-	-	-	-	nsSNV
4	88537072	88537081	TAGCAGTGAC	T	DSPP	4	10	-	-	-	-	-	IF del
3	58084486	58084486	A	G	FLNB	9	17	rs145673747	rs145673747	-	-	-	nsSNV
5	162909996	162909996	T	C	HMMR-AS1	103	31	-	-	-	-	-	ncRNA
12	56428760	56428760	C	CG	IKZF4	93	6	-	-	-	-	-	FS ins
5	128440934	128440934	A	G	ISOC1	51	35	-	-	-	-	-	sSNV
16	919000	919000	G	A	LMF1	36	25	-	-	-	-	-	sSNV
10	29776325	29776327	TAA	T	LOC102724316	10	6	-	-	-	-	-	ncRNA
2	66667039	66667039	C	CG	MEIS1	84	7	-	-	-	-	-	FS ins
17	4796837	4796837	G	A	MINK1	26	47	-	-	-	-	-	nsSNV
22	46507545	46507547	AAG	A	MIRLET7BHG	86	8	-	-	-	-	-	ncRNA
12	40918902	40918902	T	C	MUC19	42	3	-	-	-	-	-	UN
5	16694605	16694605	G	GC	MYO10	158	10	-	-	-	-	-	FS ins
3	196669594	196669594	C	T	NCBP2-AS2	9	19	-	-	-	-	-	ncRNA
15	24921468	24921468	T	TG	NPAP1	83	8	-	-	-	-	-	FS ins
9	131222915	131222915	A	AC	ODF2	80	9	-	-	-	-	-	FS ins
16	67695546	67695546	G	GC	PARD6A	184	14	-	-	-	-	-	FS ins
11	3845151	3845151	C	CG	PGAP2	120	12	-	-	-	-	-	FS ins
17	6374655	6374655	T	A	PITPNM3	3	12	-	-	-	-	-	nsSNV
7	75157156	75157156	T	TC	PMS2P3	61	7	-	-	-	-	-	ncRNA
22	46631080	46631080	A	C	PPARA	13	4	-	-	-	-	-	nsSNV
9	127920662	127920662	C	T	PPP6C	57	10	-	-	-	-	O	splicing
14	24605476	24605476	C	T	PSME1	34	3	-	-	-	-	-	nsSNV
7	127729577	127729577	C	T	SND1	41	19	-	-	-	-	-	nsSNV
15	100889519	100889519	C	T	SPATA41	188	123	-	-	-	-	-	ncRNA
4	124323691	124323691	G	T	SPRY1	21	42	-	-	-	-	-	nsSNV
19	56029967	56029967	C	CT	SSC5D	174	15	-	-	-	-	-	FS ins
12	65269550	65269550	T	C	TBC1D30	24	54	-	-	-	-	-	sSNV
3	30664743	30664743	G	T	TGFBR2	109	10	-	-	-	-	O	nsSNV
17	7577572	7577572	T	C	TP53	6	8	-	-	O	O	O	nsSNV
19	21926904	21926904	G	T	ZNF100	68	4	-	-	-	-	-	sSNV
10	43014402	43014404	AAG	A	ZNF37BP	43	6	-	-	-	-	-	ncRNA

*NonFlagged: dbSNP files subtracting Flagged dbSNP entries. Flagged SNPs include SNPs < 1% minor allele frequency (MAF) (or unknown), mapping only once to reference assembly, flagged in dbSnp as "clinically associated".
**nsSNV: non-synonymous SNV, sSNV: synonymous SNV, FS ins: frameshift insertion, FS del: frameshift deletion, IF ins: in-frame insertion, IF del: in-frame deletion, ncRNA: non-coding RNA, UN: Unknown. transcript maps to multiple locations, all as "coding transcripts", but none has a complete ORF.

Table S3. Processed WES data
(C) AYA04

Chr	Start	End	Ref	Alt	Altered gene	T_Ref	T_Alt	dbSNP138		CVE list			Mutation context**
								All	NonFlagged*	C	V	E	
10	114181766	114181766	C	T	ACSL5	135	29	-	-	-	-	-	sSNV
10	127755315	127755315	C	A	ADAM12	177	13	-	-	-	-	-	nsSNV
5	7826867	7826867	C	T	ADCY2	183	48	-	-	-	-	-	sSNV
1	980882	980882	G	A	AGRN	157	19	rs148315419	rs148315419	-	-	-	nsSNV
2	131520783	131520783	G	A	AMER3	198	49	-	-	-	-	-	nsSNV
4	114275811	114275811	C	T	ANK2	63	12	-	-	-	-	-	nsSNV
4	125631581	125631581	A	T	ANKRD50	249	62	-	-	-	-	-	nsSNV
2	219114594	219114594	G	C	ARPC2	232	51	-	-	-	-	-	sSNV
7	150721080	150721080	G	A	ATG9B	100	6	-	-	-	-	-	UN
15	50190393	50190393	C	T	ATP8B4	100	6	-	-	-	-	-	nsSNV
1	193149870	193149870	G	A	B3GALT2	216	22	-	-	-	-	-	nsSNV
9	135458462	135458462	C	A	BARHL1	119	7	-	-	-	-	-	nsSNV
17	65924573	65924573	G	T	BPTF	234	11	-	-	-	-	-	nsSNV
17	65924590	65924590	A	G	BPTF	233	12	-	-	-	-	-	sSNV
1	11008227	11008227	C	T	C1orf127	254	65	-	-	-	-	-	sSNV
20	1187715	1187715	G	T	C20orf202	129	7	-	-	-	-	-	nsSNV
9	35674208	35674208	T	C	CA9	63	4	-	-	-	-	-	sSNV
12	2705120	2705120	T	C	CACNA1C	771	32	-	-	-	-	-	nsSNV
12	1996184	1996184	T	C	CACNA2D4	15	10	-	-	-	-	-	nsSNV
12	7653878	7653878	T	G	CD163	808	40	-	-	-	-	O	nsSNV
10	85974315	85974315	C	A	CDHR1	191	13	-	-	-	-	-	nsSNV
17	30815356	30815356	G	C	CDK5R1	236	18	-	-	-	-	-	nsSNV
17	46053334	46053334	A	G	CDK5RAP3	102	5	-	-	-	-	-	sSNV
22	17669307	17669309	GCA	G	CECR1	227	22	-	-	-	-	-	FS del
1	6166747	6166747	G	A	CHD5	53	11	rs371741094	-	-	-	-	nsSNV
2	101009849	101009849	G	A	CHST10	120	50	-	-	-	-	-	nsSNV
12	122794338	122794338	T	A	CLIP1	24	7	-	-	-	-	-	nsSNV
3	130187801	130187801	T	G	COL6A5	282	22	-	-	-	-	-	nsSNV
22	37322114	37322114	T	A	CSF2RB	130	10	-	-	-	-	-	nsSNV
2	80085214	80085214	G	A	CTNNA2	130	24	-	-	-	-	-	nsSNV
10	104596845	104596845	C	T	CYP17A1	237	18	-	-	-	-	-	nsSNV
10	94834660	94834660	A	T	CYP26A1	103	6	-	-	-	-	-	nsSNV
11	6662114	6662114	G	C	DCHS1	175	12	-	-	-	-	-	nsSNV
4	155163852	155163852	A	C	DCHS2	143	20	-	-	-	-	-	sSNV
12	9574060	9574060	A	T	DDX12P	186	6	-	-	-	-	-	ncRNA
3	38080791	38080791	G	A	DLEC1	151	45	-	-	-	-	-	sSNV
6	170592616	170592616	C	CG	DLL1	107	6	-	-	-	-	-	FS ins
17	76457652	76457652	C	A	DNAH17	105	13	-	-	-	-	-	nonsense
17	11513721	11513721	C	T	DNAH9	142	11	-	-	-	-	-	nsSNV
1	65867548	65867548	G	A	DNAJC6	147	10	rs140156759	rs140156759	-	-	-	nsSNV
4	88534407	88534409	AAT	A	DSPP	102	15	-	-	-	-	-	FS del
6	74228438	74228438	T	A	EEF1A1	128	5	-	-	-	-	-	nsSNV
2	27587675	27587675	G	A	EIF2B4	135	8	-	-	-	-	-	nonsense
6	65301613	65301613	A	C	EYS	392	20	-	-	-	-	-	nsSNV
4	187524594	187524594	G	GT	FAT1	326	82	-	-	-	-	O	FS ins
11	92534689	92534689	G	C	FAT3	87	7	-	-	-	-	-	nsSNV
3	13612717	13612717	G	C	FBLN2	27	6	-	-	-	-	-	nsSNV
1	171121179	171121179	G	A	FMO6P	414	26	-	-	-	-	-	ncRNA
12	8195263	8195263	C	T	FOXJ2	738	98	-	-	-	-	-	nsSNV
3	138665096	138665096	G	C	FOXL2	47	20	-	-	O	O	-	nsSNV
1	247694105	247694106	GC	G	GCSAML-AS1	34	12	-	-	-	-	-	ncRNA
4	44709876	44709876	T	C	GNPDA2	171	19	-	-	-	-	-	nsSNV
15	22743407	22743407	G	A	GOLGA6L1,GOLGA6L22	35	3	rs200513306	rs200513306	-	-	-	nsSNV
16	57608811	57608811	G	T	GPR114	54	7	-	-	-	-	-	sSNV
5	89923515	89923515	A	C	GPR98	112	6	-	-	-	-	-	nsSNV
3	141497247	141497247	G	A	GRK7	98	35	-	-	-	-	-	nsSNV
X	51487056	51487056	C	T	GSPT2	43	19	-	-	-	-	-	nsSNV
6	29856463	29856463	T	G	HLA-H	38	4	-	-	-	-	-	ncRNA
6	55443760	55443760	G	C	HMGCLL1	216	17	-	-	-	-	-	nsSNV
2	177016663	177016663	C	T	HOXD4	22	3	-	-	-	-	-	nsSNV
2	234079144	234079144	G	A	INPP5D	46	6	-	-	-	-	-	UN
3	12977592	12977592	C	T	IQSEC1	81	6	-	-	-	-	-	sSNV
2	227662160	227662162	CCA	C	IRS1	135	8	-	-	-	-	-	FS del
12	2926407	2926407	G	A	ITFG2	254	18	-	-	-	-	-	nsSNV
17	73736508	73736508	A	T	ITGB4	126	14	-	-	-	-	-	nsSNV
4	6037730	6037730	A	T	JAKMIP1	93	7	-	-	-	-	-	sSNV
1	39880094	39880094	C	T	KIAA0754	202	20	-	-	-	-	-	nsSNV
22	33700389	33700389	C	T	LARGE	122	6	-	-	-	-	-	nsSNV
13	53277762	53277762	A	C	LECT1	297	32	-	-	-	-	-	nsSNV

1	202266700	202266700	G	T	LGR6	190	21	-	-	-	-	-	nonsense
6	33554802	33554802	A	AAC	LINC00336	5	6	-	-	-	-	-	ncRNA
19	1556803	1556803	C	A	MEX3D	132	15	-	-	-	-	-	nonsense
15	89453045	89453045	G	A	MFGE8	32	4	-	-	-	-	O	sSNV
7	7612489	7612489	G	C	MIOS	179	15	-	-	-	-	-	nsSNV
7	7612490	7612490	G	T	MIOS	176	16	-	-	-	-	-	nsSNV
4	4864581	4864581	C	T	MSX1	63	6	-	-	-	-	-	nsSNV
11	1080576	1080576	G	A	MUC2	90	4	-	-	-	-	-	sSNV
16	48595053	48595053	G	A	N4BP1	227	65	-	-	-	-	-	nsSNV
17	7227216	7227216	G	A	NEURL4	277	27	-	-	-	-	-	sSNV
14	36988746	36988746	T	TC	NKX2-1-AS1	227	12	-	-	-	-	-	ncRNA
1	162040068	162040068	C	T	NOS1AP	222	23	-	-	-	-	-	nsSNV
11	123887008	123887008	C	A	OR10G4	210	13	-	-	-	-	-	nsSNV
11	5443470	5443470	T	G	OR51Q1	841	60	-	-	-	-	-	nsSNV
X	99662510	99662510	G	A	PCDH19	87	59	-	-	-	-	-	sSNV
4	30724475	30724475	G	T	PCDH7	234	13	-	-	-	-	O	nsSNV
8	52744090	52744090	T	C	PCMTD1	45	4	-	-	-	-	O	nsSNV
21	44180483	44180483	C	T	PDE9A	224	48	-	-	-	-	-	nsSNV
16	20387485	20387485	G	A	PDILT	79	24	-	-	-	-	-	nonsense
20	8639311	8639311	G	T	PLCB1	129	8	-	-	-	-	-	nsSNV
20	8782781	8782781	C	T	PLCB1	87	10	-	-	-	-	-	nsSNV
2	68613666	68613666	C	T	PLEK	170	14	-	-	-	-	-	nonsense
1	204226768	204226768	C	T	PLEKHA6	54	6	-	-	-	-	O	nsSNV
11	65046261	65046261	C	T	POLA2	76	30	-	-	-	-	-	sSNV
2	132021766	132021766	A	C	POTEE	79	7	-	-	-	-	-	nsSNV
7	150069952	150069952	G	C	REPIN1	140	16	-	-	-	-	-	nsSNV
10	62645914	62645914	C	T	RHOBTB1	494	101	-	-	-	-	-	nsSNV
2	114369818	114369818	G	A	RPL23AP7	4	3	-	-	-	-	-	ncRNA
8	73984036	73984036	G	C	SBSPON	100	10	-	-	-	-	-	nsSNV
11	62189812	62189812	G	A	SCGB1A1	126	8	-	-	-	-	-	nsSNV
2	165950946	165950946	A	T	SCN3A	141	7	-	-	-	-	O	nsSNV
6	76412647	76412647	T	C	SENP6	138	17	-	-	-	-	-	nsSNV
2	180008480	180008480	C	T	SESTD1	74	8	-	-	-	-	O	nsSNV
19	51170416	51170416	G	A	SHANK1	51	6	-	-	-	-	-	nsSNV
19	51918521	51918521	A	C	SIGLEC10	73	6	-	-	-	-	-	nsSNV
20	37356934	37356934	C	T	SLC32A1	218	20	-	-	-	-	-	sSNV
6	118635218	118635218	T	G	SLC35F1	414	18	-	-	-	-	-	nsSNV
15	67482790	67482790	G	A	SMAD3	56	5	-	-	-	-	-	sSNV
17	61910454	61910454	G	T	SMARCD2	76	19	-	-	-	-	-	nsSNV
2	1168851	1168851	C	T	SNTG2	62	5	-	-	-	-	-	sSNV
8	48641564	48641564	C	A	SPIDR	51	5	-	-	-	-	-	sSNV
X	138529572	138529572	A	T	SRD5A1P1	28	3	-	-	-	-	-	ncRNA
4	119979077	119979078	AC	A	SYNPO2	193	12	-	-	-	-	-	FS del
X	100531469	100531472	CATT	C	TAF7L	265	36	-	-	-	-	-	IF del
12	6566795	6566795	C	T	TAPBPL	211	10	-	-	-	-	-	sSNV
19	3600211	3600211	G	A	TBXA2R	14	6	-	-	-	-	-	nsSNV
14	24725214	24725214	G	A	TGM1	116	6	-	-	-	-	-	nsSNV
15	39885852	39885852	G	A	THBS1	40	3	-	-	-	-	-	nsSNV
15	63054504	63054504	G	A	TLN2	481	42	-	-	-	-	-	nsSNV
22	22314781	22314781	G	C	TOP3B	35	3	-	-	-	-	-	nsSNV
17	7579592	7579592	T	C	TP53	60	23	-	-	O	O	O	splicing
22	20100239	20100239	C	T	TRMT2A	72	7	-	-	-	-	-	sSNV
9	131073180	131073180	G	A	TRUB2	317	38	-	-	-	-	-	nsSNV
2	179453333	179453333	G	T	TTN	127	18	-	-	-	-	-	nsSNV
2	179589251	179589251	C	A	TTN	49	5	-	-	-	-	-	nsSNV
19	4944397	4944397	C	T	UHRF1	193	71	-	-	-	-	-	UN
19	17751431	17751431	C	T	UNC13A	53	10	-	-	-	-	-	sSNV
17	5037195	5037195	G	A	USP6	114	6	rs74900103	rs74900103	O	-	-	nsSNV
X	65252418	65252418	A	T	VSIG4	62	5	-	-	-	-	-	nsSNV
12	6161801	6161801	C	T	VWF	190	11	-	-	-	-	-	sSNV
12	863316	863316	G	A	WNK1	101	13	-	-	-	-	-	sSNV
3	55508509	55508509	G	A	WNT5A	53	4	-	-	-	-	-	sSNV
14	77605591	77605591	G	A	ZDHHC22	60	21	-	-	-	-	-	nsSNV
5	180277628	180277628	G	A	ZFP62	340	47	-	-	-	-	-	sSNV
6	87964581	87964581	C	A	ZNF292	388	62	-	-	-	-	O	nsSNV
19	21992579	21992579	C	G	ZNF43	89	11	-	-	-	-	-	nsSNV
1	182026125	182026125	C	T	ZNF648	224	34	-	-	-	-	-	nsSNV

*NonFlagged: dbSNP files subtracting Flagged dbSNP entries. Flagged SNPs include SNPs < 1% minor allele frequency (MAF) (or unknown), mapping only once to reference assembly, flagged in dbSnp as "clinically associated".

**nsSNV: non-synonymous SNV, sSNV: synonymous SNV, FS ins: frameshift insertion, FS del: frameshift deletion, IF ins: in-frame insertion, IF del: in-frame deletion, ncRNA: non-coding RNA, UN: Unknown. transcript maps to multiple locations, all as "coding transcripts", but none has a complete ORF.

Table S3. Processed WES data
(D) AYA06

Chr	Start	End	Ref	Alt	Altered gene	T_Ref	T_Alt	dbSNP138		CVE list			Mutation context**
								All	NonFlagged*	C	V	E	
13	25745349	25745349	G	GC	AMER2	95	8	-	-	-	-	-	FS ins
9	95599345	95599345	G	GA	ANKRD19P	6	7	-	-	-	-	-	ncRNA
13	70713509	70713509	A	ACTG	ATXN8OS	27	7	-	-	-	-	-	ncRNA
19	51892382	51892382	G	GC	C19orf84	186	16	-	-	-	-	-	FS ins
18	20716014	20716023	GGGCGCCG	G	CABLES1	15	9	-	-	-	-	-	IF del
6	7389434	7389434	C	T	CAGE1	126	6	-	-	-	-	-	splicing
7	93055822	93055822	C	T	CALCR	297	11	-	-	-	-	-	nsSNV
8	82644888	82644888	A	AG	CHMP4C	188	10	-	-	-	-	-	FS ins
22	37769495	37769495	T	TC	ELFN2	80	6	-	-	-	-	-	FS ins
17	71205858	71205861	ATGC	A	FAM104A	102	6	-	-	-	-	-	IF del
17	18682505	18682505	T	C	FBXW10	32	4	rs1024657	rs1024657	-	-	-	nsSNV
4	153896047	153896047	A	AC	FHDC1	97	7	-	-	-	-	-	FS ins
1	152285137	152285137	G	T	FLG	104	5	rs3120654	rs3120654	-	-	-	nsSNV
15	30905994	30905994	G	A	GOLGA8H	36	5	rs4042347	rs199794386	-	-	-	nsSNV
1	13183492	13183492	T	C	HNRNPCL2	11	4	-	-	-	-	-	sSNV
2	170374777	170374777	G	A	KLHL41	59	4	-	-	-	-	-	nsSNV
12	25398281	25398281	C	T	KRAS	128	10	rs112445441	-	O	O	O	nsSNV
17	39305775	39305775	T	TGGCAGCA	KRTAP4-5	29	14	-	-	-	-	O	IF ins
17	39346595	39346595	T	TA	KRTAP9-1	155	13	-	-	-	-	-	FS ins
15	90320134	90320146	AGGGCAGG	A	MESP2	3	21	-	-	-	-	-	IF del
3	196733535	196733535	C	CG	MFI2	77	7	-	-	-	-	-	FS ins
5	79950724	79950724	G	C	MSH3	45	5	rs2001675	rs2001675	-	-	-	nsSNV
11	55579145	55579145	C	T	OR5L1	31	3	-	-	-	-	-	nsSNV
1	158532510	158532510	C	A	OR6P1	304	10	-	-	-	-	-	nsSNV
19	7607678	7607678	C	T	PNPLA6	125	5	-	-	-	-	-	nsSNV
1	79002163	79002163	C	T	PTGFR	238	8	-	-	-	-	-	nonsense
1	64605927	64605927	G	A	ROR1	472	13	-	-	-	-	-	nsSNV
19	39923952	39923952	G	A	RPS16	29	3	-	-	-	-	-	sSNV
12	123005948	123005948	C	T	RSRC2	193	7	-	-	-	-	-	nsSNV
21	36410869	36410871	CCT	C	RUNX1-IT1	42	10	-	-	-	-	-	ncRNA
12	109017650	109017680	GGAGTGGT	G	SELPLG	272	85	-	-	-	-	-	IF del
21	34927468	34927468	C	G	SON	40	6	-	-	-	-	-	sSNV
18	12449779	12449779	G	A	SPIRE1	340	11	-	-	-	-	-	nsSNV
12	22354743	22354743	G	A	ST8SIA1	284	9	-	-	-	-	-	nsSNV
9	113137744	113137745	CT	C	SVEP1	46	6	-	-	-	-	-	splicing
15	90118864	90118864	G	GC	TICRR	117	9	-	-	-	-	-	FS ins
X	73045808	73045808	G	A	TSIX,XIST	182	10	-	-	-	-	-	ncRNA
17	5037195	5037195	G	A	USP6	67	6	rs74900103	rs74900103	O	-	-	nsSNV
8	146201230	146201233	TACA	T	ZNF252P	91	6	-	-	-	-	-	ncRNA
19	58600100	58600100	T	TGG	ZSCAN18	121	10	-	-	-	-	-	FS ins

*NonFlagged: dbSNP files subtracting Flagged dbSNP entries. Flagged SNPs include SNPs < 1% minor allele frequency (MAF) (or unknown), mapping only once to reference assembly, flagged in dbSnp as "clinically associated".
**nsSNV: non-synonymous SNV, sSNV: synonymous SNV, FS ins: frameshift insertion, FS del: frameshift deletion, IF ins: in-frame insertion, IF del: in-frame deletion, ncRNA: non-coding RNA, UN: Unknown. transcript maps to multiple locations, all as "coding transcripts", but none has a complete ORF.

Table S3. Processed WES data
(E) AYA07

Chr	Start	End	Ref	Alt	Altered gene	T_Ref	T_Alt	dbSNP138		CVE list			Mutation context**
								All	NonFlagged*	C	V	E	
19	58864015	58864015	G	A	A1BG-AS1	17	4	-	-	-	-	-	ncRNA
2	169791885	169791885	C	T	ABCB11	36	25	-	-	-	-	-	sSNV
16	16282712	16282712	G	T	ABCC6	25	4	-	-	-	-	-	sSNV
3	100566447	100566447	C	A	ABI3BP	133	5	-	-	-	-	-	sSNV
17	61559009	61559009	G	T	ACE	73	5	-	-	-	-	-	nsSNV
17	61559937	61559937	G	T	ACE	124	7	-	-	-	-	-	nsSNV
17	61568684	61568684	G	T	ACE	32	4	-	-	-	-	-	nsSNV
11	76709805	76709805	A	T	ACER3	101	40	-	-	-	-	-	splicing
11	76709806	76709806	G	T	ACER3	102	39	-	-	-	-	-	splicing
1	120436968	120436968	C	A	ADAM30	93	5	-	-	-	-	-	sSNV
5	33549374	33549374	G	T	ADAMTS12	121	8	rs77581578	rs77581578	-	-	-	nsSNV
5	33891883	33891883	C	A	ADAMTS12	69	5	-	-	-	-	-	nsSNV
9	18906820	18906820	C	A	ADAMTSL1	40	5	-	-	-	-	-	nsSNV
9	136402633	136402633	G	T	ADAMTSL2	39	4	-	-	-	-	-	nsSNV
6	147136309	147136309	C	A	ADGB	60	5	-	-	-	-	-	nsSNV
4	100232023	100232023	A	T	ADH1B	162	38	-	-	-	-	-	sSNV
8	26721911	26721911	G	A	ADRA1A	20	22	-	-	-	-	-	sSNV
2	236957852	236957852	G	T	AGAP1	59	5	-	-	-	-	-	nsSNV
12	58130904	58130904	C	A	AGAP2	37	3	-	-	-	-	-	nsSNV
6	151670599	151670599	C	A	AKAP12	28	4	-	-	-	-	-	nsSNV
15	86287887	86287887	G	T	AKAP13	46	4	-	-	-	-	-	nsSNV
19	15484021	15484021	C	A	AKAP8	61	5	-	-	-	-	-	nsSNV
9	117118302	117118302	G	T	AKNA	65	5	-	-	-	-	-	sSNV
11	77832169	77832169	C	G	ALG8	226	74	-	-	-	-	-	nsSNV
19	36501574	36501574	G	A	ALKBH6	135	53	-	-	-	-	-	sSNV
1	21887134	21887134	C	A	ALPL	55	5	-	-	-	-	-	nsSNV
3	46718456	46718456	G	T	ALS2CL	63	5	-	-	-	-	-	nsSNV
12	31850856	31850856	G	T	AMN1	201	8	-	-	-	-	-	nsSNV
12	31841951	31841951	G	T	AMN1	137	7	-	-	-	-	-	sSNV
11	113270241	113270241	G	T	ANKK1	64	6	-	-	-	-	-	nsSNV
5	72851348	72851348	C	A	ANKRA2	149	7	-	-	-	-	-	nsSNV
16	89354947	89354947	C	A	ANKRD11	79	5	-	-	-	-	-	nsSNV
19	33095298	33095298	C	A	ANKRD27	80	5	-	-	-	-	-	nsSNV
5	79855084	79855084	G	A	ANKRD34B	42	16	-	-	-	-	-	nsSNV
15	40574762	40574762	G	T	ANKRD63	37	4	-	-	-	-	-	nsSNV
8	124748071	124748071	C	A	ANXA13	57	5	-	-	-	-	-	nsSNV
16	71766978	71766978	G	T	AP1G1	39	4	-	-	-	-	-	nsSNV
1	114440492	114440492	G	T	AP4B1	66	5	-	-	-	-	-	sSNV
14	20924871	20924871	C	CA	APEX1	55	34	-	-	-	-	-	FS ins
19	36365496	36365496	C	A	APLP1	69	6	-	-	-	-	-	nsSNV
14	75140777	75140777	G	T	AREL1	69	5	rs17853116	rs17853116	-	-	-	nsSNV
20	47641993	47641993	G	A	ARFGEF2	96	27	-	-	-	-	-	sSNV
17	12887975	12887975	C	A	ARHGAP44	83	5	-	-	-	-	-	sSNV
1	17930026	17930026	C	A	ARHGEF10L	39	4	-	-	-	-	-	nsSNV
2	39185184	39185184	G	T	ARHGEF33	56	17	-	-	-	-	O	nsSNV
2	39185185	39185185	G	T	ARHGEF33	55	17	-	-	-	-	O	nsSNV
2	97217432	97217432	G	T	ARID5A	25	4	-	-	-	-	-	nsSNV
6	109220994	109220994	G	T	ARMC2	24	4	-	-	-	-	-	nsSNV
3	35778792	35778792	C	A	ARPP21	36	4	-	-	-	-	-	nsSNV
14	104577930	104577930	C	A	ASPG	24	4	-	-	-	-	-	nsSNV
8	124408451	124408451	G	T	ATAD2	43	3	-	-	-	-	O	sSNV
14	96800086	96800086	C	A	ATG2B	85	5	-	-	-	-	-	sSNV
6	106764022	106764022	G	A	ATG5	83	10	-	-	-	-	O	nsSNV
5	160042911	160042911	G	T	ATP10B	126	8	-	-	-	-	-	nsSNV
16	84459400	84459400	G	T	ATP2C2	45	4	-	-	-	-	-	nsSNV
2	71185508	71185508	G	A	ATP6V1B1	50	18	-	-	-	-	-	nsSNV
6	31514448	31514448	C	A	ATP6V1G2-DDX	303	14	rs370511629	rs370511629	-	-	-	ncRNA
13	52511698	52511698	G	T	ATP7B	65	6	-	-	-	-	-	nsSNV
13	52516578	52516578	G	T	ATP7B	29	4	-	-	-	-	-	nsSNV
10	116853763	116853763	G	T	ATRNL1	40	4	-	-	-	-	-	nsSNV
7	70255271	70255271	C	A	AUTS2	107	6	-	-	-	-	-	sSNV
19	41765733	41765733	C	A	AXL	230	8	-	-	-	-	-	nsSNV
11	134251848	134251848	G	T	B3GAT1	81	5	-	-	-	-	-	nsSNV

11	372919	372919	G	A	B4GALNT4	14	10	-	-	-	-	-	nsSNV
17	79007094	79007094	C	A	BAIAP2-AS1	57	5	-	-	-	-	-	ncRNA
1	147096518	147096518	C	A	BCL9	134	7	-	-	O	-	-	nsSNV
20	55840829	55840829	G	T	BMP7	147	7	-	-	-	-	-	nsSNV
19	15350596	15350596	G	T	BRD4	24	4	-	-	O	-	-	nsSNV
19	15378291	15378291	G	T	BRD4	41	4	-	-	O	-	-	sSNV
5	878551	878551	A	G	BRD9	91	10	rs145107515	rs145107515	-	-	-	nsSNV
16	2260174	2260174	G	T	BRICD5	31	4	-	-	-	-	-	nsSNV
9	121930383	121930383	C	A	BRINP1	31	7	-	-	-	-	-	nsSNV
10	93788546	93788546	G	A	BTAF1	45	21	-	-	-	-	-	splicing
11	57512463	57512463	C	T	BTBD18	168	46	-	-	-	-	-	nsSNV
6	26385475	26385475	G	T	BTN2A2	71	6	-	-	-	-	-	nsSNV
5	180420152	180420152	G	T	BTNL3	32	4	-	-	-	-	-	nsSNV
10	50533403	50533403	C	A	C10orf71	81	5	-	-	-	-	-	nsSNV
14	29261243	29261243	G	T	C14orf23	85	5	-	-	-	-	-	ncRNA
15	76496640	76496640	G	T	C15orf27	38	4	-	-	-	-	-	nsSNV
15	80215201	80215201	C	A	C15orf37	183	7	-	-	-	-	-	ncRNA
1	11008789	11008789	C	A	C1orf127	34	4	-	-	-	-	-	nsSNV
1	60520883	60520883	T	A	C1orf87	111	54	-	-	-	-	-	nsSNV
17	77044053	77044053	G	C	C1QTNF1	50	16	-	-	-	-	-	nsSNV
15	62360729	62360729	G	C	C2CD4A	23	17	-	-	-	-	-	nsSNV
6	30615198	30615198	C	A	C6orf136	34	4	-	-	-	-	-	nsSNV
8	146278141	146278141	C	A	C8orf33	23	4	-	-	-	-	-	nsSNV
2	27444100	27444100	G	T	CAD	51	4	-	-	-	-	-	sSNV
17	76993222	76993222	C	A	CANT1	186	8	-	-	O	-	-	nsSNV
6	17507933	17507933	G	T	CAP2	104	7	-	-	-	-	-	nsSNV
15	42682292	42682292	C	A	CAPN3	81	7	-	-	-	-	-	sSNV
12	75678861	75678861	C	T	CAPS2	206	29	-	-	-	-	-	splicing
19	5751734	5751734	C	A	CATSPERD	17	3	-	-	-	-	-	nsSNV
19	48800504	48800504	G	T	CCDC114	83	5	-	-	-	-	-	nsSNV
5	68616173	68616173	C	A	CCDC125	118	6	-	-	-	-	-	nsSNV
3	42798707	42798707	C	A	CCDC13	123	6	-	-	-	-	-	splicing
X	50052253	50052253	C	A	CCNB3	33	5	-	-	-	-	-	nsSNV
X	50089805	50089805	G	T	CCNB3	68	5	-	-	-	-	-	nsSNV
1	160811244	160811244	C	A	CD244	87	6	-	-	-	-	-	nsSNV
15	73995241	73995241	G	T	CD276	6	4	-	-	-	-	-	nsSNV
3	107770787	107770787	C	A	CD47	117	6	-	-	-	-	-	nsSNV
14	103447248	103447248	C	T	CDC42BPB	182	47	-	-	-	-	-	sSNV
20	58569523	58569523	T	A	CDH26	115	19	-	-	-	-	-	nsSNV
20	60348183	60348183	C	A	CDH4	29	4	-	-	-	-	-	nsSNV
19	42083630	42083630	G	T	CEACAM21	89	7	-	-	-	-	-	nsSNV
16	81061864	81061864	C	A	CENPN	17	3	-	-	-	-	-	nsSNV
3	101484336	101484336	G	T	CEP97	112	6	-	-	-	-	-	nsSNV
2	202014412	202014412	G	T	CFLAR-AS1	130	6	-	-	-	-	-	ncRNA
1	151506546	151506546	G	A	CGN	12	7	-	-	-	-	-	sSNV
12	6701875	6701875	G	T	CHD4	82	5	-	-	-	-	O	nsSNV
22	29121232	29121232	C	A	CHEK2	172	8	rs142966756	rs142966756	O	-	-	nsSNV
2	175614897	175614897	C	T	CHRNA1	19	5	-	-	-	-	-	nsSNV
15	78894408	78894408	G	T	CHRNA3	65	39	-	-	-	-	-	sSNV
18	24604117	24604117	C	A	CHST9	32	4	-	-	-	-	-	nsSNV
16	57473198	57473198	G	T	CIAPIN1	82	6	-	-	-	-	-	nsSNV
19	42798827	42798827	C	A	CIC	142	6	-	-	O	O	O	nsSNV
7	139228997	139228997	G	T	CLEC2L	45	4	-	-	-	-	-	nsSNV
5	157243839	157243839	C	A	CLINT1	178	7	-	-	-	-	-	splicing
19	45494142	45494142	C	A	CLPTM1	31	4	-	-	-	-	-	nsSNV
3	140178505	140178505	C	A	CLSTN2	86	5	-	-	-	-	-	sSNV
5	79026940	79026940	G	A	CMYA5	140	71	-	-	-	-	-	sSNV
16	57931733	57931733	G	A	CNGB1	58	43	-	-	-	-	-	nsSNV
6	116442151	116442151	C	A	COL10A1	66	6	-	-	-	-	-	nsSNV
6	33140415	33140415	C	A	COL11A2	108	6	-	-	-	-	-	splicing
10	71712646	71712646	G	A	COL13A1	21	10	-	-	-	-	-	sSNV
13	111109773	111109773	G	T	COL4A2	64	5	-	-	-	-	O	nsSNV
2	238249368	238249368	G	T	COL6A3	121	6	-	-	-	-	-	nsSNV
3	99513773	99513773	G	A	COL8A1	37	11	-	-	-	-	-	nsSNV
3	128979575	128979575	G	T	COPG1	35	4	-	-	-	-	-	sSNV
20	30226879	30226879	G	T	COX4I2	16	4	-	-	-	-	-	nsSNV
4	15067827	15067827	G	T	CPEB2	84	5	-	-	-	-	O	nsSNV
4	15005287	15005287	C	A	CPEB2	94	6	-	-	-	-	O	sSNV

20	2776413	2776413	G	T	CPXM1	93	5	-	-	-	-	-	nsSNV
20	20023059	20023059	G	A	CRNKL1	269	70	-	-	-	-	-	sSNV
12	53554023	53554023	C	A	CSAD	91	5	-	-	-	-	-	sSNV
1	34554624	34554624	G	A	CSMD2	33	22	-	-	-	-	-	sSNV
12	51457820	51457820	C	T	CSRNP2	78	19	-	-	-	-	-	sSNV
7	117431318	117431318	G	T	CTTNBP2	40	3	-	-	-	-	-	sSNV
7	101892210	101892210	G	T	CUX1	24	3	-	-	-	-	-	nsSNV
2	172398266	172398266	G	T	CYBRD1	12	3	-	-	-	-	-	nsSNV
8	143956518	143956518	G	T	CYP11B1	68	4	-	-	-	-	-	nsSNV
19	41383099	41383099	G	T	CYP2A7	117	7	-	-	-	-	-	nsSNV
8	59409400	59409400	G	T	CYP7A1	86	6	-	-	-	-	-	nsSNV
9	124522316	124522316	G	T	DAB2IP	119	7	-	-	-	-	-	nsSNV
17	42807281	42807281	G	T	DBF4B	38	4	-	-	-	-	-	nsSNV
1	168012337	168012337	C	A	DCAF6	83	5	-	-	-	-	-	nsSNV
11	6644232	6644232	C	A	DCHS1	37	3	-	-	-	-	-	nsSNV
11	6655252	6655252	C	A	DCHS1	131	7	-	-	-	-	-	splicing
1	85816130	85816130	C	A	DDAH1	56	5	-	-	-	-	-	nsSNV
11	61070099	61070099	G	T	DDB1	80	5	-	-	-	-	-	nsSNV
22	24313802	24313802	G	A	DDT	390	17	rs140621020	rs140621020	-	-	-	nsSNV
9	118163487	118163487	C	A	DEC1	22	5	-	-	-	-	-	nsSNV
7	24745809	24745809	G	T	DFNA5	40	4	-	-	-	-	-	nsSNV
17	9680569	9680569	C	A	DHRS7C	57	18	-	-	-	-	-	nsSNV
15	40660326	40660326	G	C	DISP2	13	4	-	-	-	-	-	sSNV
16	56228120	56228120	C	A	DKFZP434H168	74	6	-	-	-	-	-	ncRNA
8	42234516	42234516	C	A	DKK4	68	5	-	-	-	-	-	sSNV
1	64014766	64014766	G	T	DLEU2L	94	6	-	-	-	-	-	ncRNA
3	57430951	57430951	C	A	DNAH12	118	27	-	-	-	-	-	UN
10	129209158	129209158	G	T	DOCK1	30	4	-	-	-	-	-	nsSNV
10	129242541	129242541	C	A	DOCK1	40	3	-	-	-	-	-	nonsense
2	225661798	225661798	T	G	DOCK10	27	17	-	-	-	-	-	nsSNV
17	1939927	1939927	C	A	DPH1	83	5	-	-	-	-	-	nsSNV
21	42064876	42064876	C	A	DSCAM	74	4	-	-	-	-	-	nsSNV
15	45454039	45454039	C	A	DUOX1	107	6	-	-	-	-	-	sSNV
1	161719802	161719802	G	T	DUSP12	45	4	-	-	-	-	-	nsSNV
2	71791262	71791262	C	A	DYSF	113	8	-	-	-	-	-	sSNV
6	139210111	139210111	G	T	ECT2L	41	4	-	-	O	-	-	nsSNV
14	23829065	23829065	C	T	EFS	17	6	-	-	-	-	-	nsSNV
14	34419953	34419953	G	A	EGLN3	54	37	-	-	-	-	-	sSNV
6	31856031	31856031	G	T	EHMT2	92	5	-	-	-	-	-	nsSNV
11	107501263	107501263	G	T	ELMOD1	105	5	-	-	-	-	-	sSNV
3	10044615	10044615	A	T	EMC3-AS1	138	68	-	-	-	-	-	ncRNA
14	89087518	89087518	C	A	EML5	213	8	-	-	-	-	-	nsSNV
1	225692753	225692753	C	A	ENAH	36	3	-	-	-	-	O	nsSNV
3	40464384	40464384	G	T	ENTPD3	27	3	-	-	-	-	-	nsSNV
8	23290466	23290466	G	T	ENTPD4	35	3	-	-	-	-	-	sSNV
22	41573677	41573677	G	T	EP300	105	7	-	-	O	O	O	nsSNV
12	15776223	15776224	TA	T	EPS8	121	12	-	-	-	-	-	splicing
12	15774299	15774299	G	A	EPS8	101	18	-	-	-	-	-	sSNV
9	27284961	27284961	A	C	EQTN	20	16	-	-	-	-	-	nsSNV
6	170176597	170176597	C	A	ERMARD	65	5	-	-	-	-	-	nsSNV
12	53680501	53680501	C	A	ESPL1	83	5	-	-	-	-	-	sSNV
7	13935516	13935516	G	T	ETV1	129	7	-	-	O	-	-	nsSNV
4	5624568	5624568	G	T	EVC2	85	5	-	-	-	-	-	nsSNV
22	29695224	29695224	G	T	EWSR1	7	3	-	-	O	-	-	sSNV
19	17000816	17000816	C	A	F2RL3	25	3	-	-	-	-	-	nsSNV
3	197880130	197880139	GGCAGCAG	G	FAM157A	8	8	-	-	-	-	-	IF del
11	6245450	6245450	G	T	FAM160A2	31	4	-	-	-	-	-	nsSNV
4	17710901	17710901	C	A	FAM184B	41	14	-	-	-	-	-	nsSNV
4	2698202	2698202	G	T	FAM193A	114	7	-	-	-	-	-	nsSNV
9	34832628	34832628	G	T	FAM205B	38	4	-	-	-	-	-	ncRNA
10	124591789	124591789	G	C	FAM24B-CUZD1	133	5	-	-	-	-	-	ncRNA
X	34961658	34961658	C	A	FAM47B	41	4	-	-	-	-	-	nsSNV
17	80053270	80053270	G	T	FASN	56	5	-	-	-	-	-	nsSNV
4	187521205	187521205	C	A	FAT1	55	5	-	-	-	-	O	nsSNV
22	45921519	45921519	C	A	FBLN1	8	3	-	-	-	-	-	sSNV
3	33416815	33416815	G	T	FBXL2	38	4	-	-	-	-	-	nsSNV
2	73496555	73496555	G	T	FBXO41	42	4	-	-	-	-	-	sSNV
22	23605315	23605315	G	T	FBXW4P1	169	11	-	-	-	-	-	ncRNA

19	40362919	40362919	C	A	FCGBP	127	6	-	-	-	-	-	nsSNV
1	157769873	157769873	T	A	FCRL1	34	25	-	-	-	-	-	nonsense
14	24601561	24601561	G	A	FITM1	43	26	-	-	-	-	-	sSNV
15	50646423	50646423	C	A	FLJ10038,GABP	43	4	-	-	-	-	-	ncRNA
5	180047702	180047702	C	A	FLT4	126	7	-	-	-	-	-	nsSNV
12	2983409	2983409	G	T	FOXM1	180	8	-	-	-	-	-	nsSNV
14	89647120	89647120	C	A	FOXN3	46	5	-	-	-	-	-	nsSNV
9	14747005	14747005	C	A	FREM1	21	4	-	-	-	-	-	nsSNV
3	69246102	69246102	C	T	FRMD4B	111	38	-	-	-	-	-	nonsense
5	132939620	132939620	C	A	FSTL4	35	13	-	-	-	-	-	nsSNV
16	53907763	53907763	C	A	FTO	95	5	-	-	-	-	-	nsSNV
9	133501811	133501811	T	A	FUBP3	38	19	-	-	-	-	-	nsSNV
9	133501812	133501812	G	T	FUBP3	38	19	-	-	-	-	-	nsSNV
11	94278261	94278261	G	T	FUT4	186	7	-	-	-	-	-	nsSNV
7	151818052	151818052	G	T	GALNT11	41	4	-	-	-	-	-	nsSNV
9	101570334	101570334	C	A	GALNT12	16	3	-	-	-	-	-	sSNV
11	62398136	62398136	C	T	GANAB	151	35	-	-	-	-	-	nonsense
21	34889797	34889797	C	A	GART	225	8	-	-	-	-	-	nsSNV
3	128199862	128199862	C	A	GATA2	83	5	-	-	O	O	-	stoploss
16	19528385	19528385	T	C	GDE1	64	38	-	-	-	-	-	nsSNV
5	154287202	154287202	C	A	GEMIN5	90	5	-	-	-	-	-	nsSNV
6	13365080	13365080	C	A	GFOD1	78	5	-	-	-	-	-	sSNV
17	73238423	73238423	C	G	GGA3	130	81	-	-	-	-	-	sSNV
5	42689093	42689093	G	A	GHR	34	9	-	-	-	-	-	nsSNV
7	100281860	100281860	C	A	GIGYF1	28	4	-	-	-	-	-	nsSNV
2	233674453	233674453	G	T	GIGYF2	44	4	-	-	-	-	O	sSNV
2	220107545	220107545	G	A	GLB1L	111	68	-	-	-	-	-	nsSNV
7	42004664	42004664	C	A	GLI3	132	7	-	-	-	-	-	nsSNV
X	102979529	102979529	G	T	GLRA4	11	3	-	-	-	-	-	sSNV
19	48182705	48182705	G	T	GLTSCR1	12	9	-	-	-	-	O	nsSNV
2	70057081	70057081	G	T	GMCL1	52	21	-	-	-	-	-	nsSNV
5	180664645	180664645	G	T	GNB2L1	93	6	-	-	-	-	-	sSNV
11	62476192	62476192	C	A	GNG3	180	8	-	-	-	-	-	nsSNV
12	21659829	21659829	G	T	GOLT1B	270	10	-	-	-	-	-	nsSNV
3	100374024	100374024	G	T	GPR128	212	9	-	-	-	-	-	nsSNV
X	150349070	150349070	C	A	GPR50	93	5	-	-	-	-	-	nsSNV
1	110086520	110086520	G	T	GPR61	111	6	-	-	-	-	-	sSNV
12	66911756	66911756	C	A	GRIP1	132	7	-	-	-	-	-	nsSNV
3	141535841	141535841	G	T	GRK7	64	5	-	-	-	-	-	sSNV
19	42744186	42744186	C	A	GSK3A	172	9	-	-	-	-	-	nsSNV
9	124044097	124044097	C	A	GSN-AS1	91	5	-	-	-	-	-	ncRNA
8	30557600	30557600	G	T	GSR	65	5	-	-	-	-	-	sSNV
6	43593255	43593255	C	A	GTPBP2	90	5	-	-	-	-	-	nsSNV
17	7909924	7909924	C	A	GUCY2D	33	3	-	-	-	-	-	sSNV
6	105298872	105298872	C	A	HACE1	32	23	-	-	-	-	-	
6	105298873	105298873	T	C	HACE1	32	23	-	-	-	-	-	
19	17160821	17160821	G	T	HAUS8	61	5	-	-	-	-	O	sSNV
6	135359042	135359042	C	A	HBS1L	25	3	-	-	-	-	-	nonsense
X	153222389	153222389	C	A	HCFC1	23	3	-	-	-	-	-	nsSNV
X	153228808	153228808	C	A	HCFC1	26	3	-	-	-	-	-	nsSNV
14	73976017	73976017	C	A	HEATR4	114	6	-	-	-	-	-	nsSNV
12	112630465	112630465	G	T	HECTD4	59	5	-	-	-	-	-	nsSNV
7	43490477	43490477	C	A	HECW1	43	4	-	-	-	-	-	nsSNV
2	197106886	197106886	C	A	HECW2	36	3	-	-	-	-	-	nsSNV
17	80395108	80395108	G	T	HEXDC	40	4	-	-	-	-	-	nsSNV
12	123342759	123342759	G	T	HIP1R	42	4	-	-	-	-	-	sSNV
1	114483207	114483207	C	A	HIPK1	87	5	-	-	-	-	-	nsSNV
6	26017368	26017368	C	A	HIST1H1A	82	5	-	-	-	-	-	nsSNV
6	26107800	26107800	C	A	HIST1H1T	191	8	-	-	-	-	-	nsSNV
1	42049707	42049707	C	A	HIVEP3	78	5	-	-	-	-	-	nsSNV
1	185972857	185972857	C	A	HMCN1	121	6	-	-	-	-	O	sSNV
5	149431301	149431301	C	A	HMGXB3	142	6	-	-	-	-	-	nsSNV
10	43883315	43883315	C	A	HNRNPF	39	4	-	-	-	-	-	nsSNV
17	46688008	46688008	G	T	HOXB7	89	5	-	-	-	-	-	sSNV
12	54383022	54383022	C	A	HOXC10	72	5	-	-	-	-	-	nsSNV
15	58983868	58983872	TCTTC	T	HSP90AB4P	37	6	-	-	-	-	-	ncRNA
11	122929117	122929117	C	A	HSPA8	72	5	-	-	-	-	-	nsSNV
1	23519665	23519665	G	T	HTR1D	70	5	-	-	-	-	-	nsSNV

1	19992892	19992892	C	A	HTR6	50	4	-	-	-	-	-	nsSNV
11	118925253	118925253	G	T	HYOU1	53	5	-	-	-	-	-	nsSNV
9	95003198	95003198	G	T	IARS	33	4	-	-	-	-	-	nsSNV
3	50326697	50326697	G	T	IFRD2	31	4	-	-	-	-	-	sSNV
3	129221570	129221570	C	A	IFT122	80	5	-	-	-	-	-	nsSNV
16	1841727	1841727	C	A	IGFALS	23	3	-	-	-	-	-	nsSNV
7	50444295	50444295	G	C	IKZF1	49	5	-	-	O	O	-	sSNV
2	102782717	102782717	C	A	IL1R1	112	5	-	-	-	-	-	sSNV
2	86371667	86371667	G	C	IMMT	57	9	-	-	-	-	-	sSNV
10	121583332	121583332	C	A	INPP5F	39	5	-	-	-	-	-	nsSNV
20	20350147	20350148	CG	C	INSM1	57	13	-	-	-	-	-	FS del
1	153727215	153727215	G	A	INTS3	16	9	-	-	-	-	-	sSNV
5	61897648	61897648	C	A	IPO11	80	5	-	-	-	-	-	sSNV
12	30829838	30829838	G	T	IPO8	131	6	-	-	-	-	-	nsSNV
3	51812713	51812713	G	T	IQCF6	87	4	-	-	-	-	-	sSNV
X	53268413	53268413	G	T	IQSEC2	67	5	-	-	-	-	-	nsSNV
5	150227805	150227805	G	A	IRGM	177	64	-	-	-	-	-	sSNV
16	54319495	54319495	C	A	IRX3	72	4	-	-	-	-	-	nsSNV
16	71958692	71958692	T	TAAG	IST1	31	16	-	-	-	-	-	IF ins
16	31424454	31424454	C	A	ITGAD	60	5	-	-	-	-	-	nsSNV
10	33200428	33200428	C	A	ITGB1	74	6	-	-	-	-	-	nsSNV
15	41795205	41795205	C	G	ITPKA	39	17	-	-	-	-	-	sSNV
3	4687151	4687151	G	T	ITPR1	86	5	-	-	-	-	-	nsSNV
8	1951175	1951175	C	A	KBTBD11	21	3	-	-	-	-	-	nsSNV
20	47989847	47989847	C	A	KCNB1	123	6	-	-	-	-	-	sSNV
20	62045472	62045472	G	T	KCNQ2	47	5	-	-	-	-	-	nsSNV
20	62103545	62103545	C	A	KCNQ2	138	6	-	-	-	-	-	nsSNV
1	196438199	196438199	C	A	KCNT2	31	4	-	-	-	-	O	splicing
8	110984623	110984623	G	T	KCNV1	118	7	-	-	-	-	-	sSNV
12	109907350	109907350	C	A	KCTD10	38	4	-	-	-	-	-	nsSNV
14	24901016	24901016	C	A	KHNYN	57	5	-	-	-	-	-	sSNV
16	15698195	15698195	G	C	KIAA0430	95	33	-	-	-	-	-	nsSNV
19	18375496	18375496	G	T	KIAA1683	98	6	-	-	-	-	-	sSNV
2	8934036	8934036	C	A	KIDINS220	68	5	-	-	-	-	-	nsSNV
20	16360543	16360544	AG	A	KIF16B	478	86	-	-	-	-	-	FS del
14	104638926	104638926	G	T	KIF26A	43	4	-	-	-	-	-	sSNV
6	33373304	33373304	C	A	KIFC1	52	4	-	-	-	-	-	sSNV
8	145694727	145694727	C	A	KIFC2	67	5	-	-	-	-	-	nsSNV
2	10188314	10188314	C	A	KLF11	239	9	-	-	-	-	-	nsSNV
7	130418131	130418131	G	T	KLF14	60	5	-	-	-	-	-	nsSNV
9	110250541	110250541	C	A	KLF4	17	3	-	-	O	O	-	nsSNV
1	899746	899746	C	A	KLHL17	29	4	-	-	-	-	-	sSNV
3	47361167	47361167	C	A	KLHL18	72	5	-	-	-	-	-	nsSNV
7	151842298	151842298	G	T	KMT2C	115	6	-	-	-	-	O	nsSNV
12	49432280	49432280	C	A	KMT2D	58	5	-	-	-	-	O	nsSNV
7	98786122	98786122	G	T	KPNA7	128	6	-	-	-	-	-	nsSNV
17	20405802	20405802	T	C	KRT16P3	19	3	-	-	-	-	-	ncRNA
12	53298675	53298675	A	C	KRT8	9	4	-	-	-	-	-	nsSNV
12	53298699	53298699	G	A	KRT8	15	4	rs201807576	rs201807576	-	-	-	nsSNV
17	39296476	39296476	G	C	KRTAP4-6	11	4	-	-	-	-	-	nsSNV
17	39346595	39346595	T	TA	KRTAP9-1	94	8	-	-	-	-	-	FS ins
14	56122813	56122813	G	T	KTN1	80	5	-	-	O	-	-	nsSNV
14	59950741	59950741	C	A	L3HYPDH	86	7	-	-	-	-	-	nsSNV
20	42142257	42142257	C	A	L3MBTL1	130	6	-	-	-	-	-	sSNV
20	9498807	9498807	T	C	LAMP5	52	9	-	-	-	-	-	nsSNV
5	154173230	154173230	C	A	LARP1	94	7	-	-	-	-	-	nsSNV
19	11226816	11226816	G	A	LDLR	10	7	-	-	-	-	-	nsSNV
13	53277821	53277821	G	T	LECT1	92	6	-	-	-	-	-	nsSNV
4	109000717	109000717	G	T	LEF1	41	4	-	-	-	-	-	nsSNV
7	103969609	103969609	C	A	LHFPL3	111	6	-	-	-	-	-	nsSNV
17	33316503	33316503	G	T	LIG3	84	5	-	-	-	-	-	nsSNV
12	50589631	50589631	G	T	LIMA1	90	6	-	-	-	-	-	nsSNV
12	81239693	81239693	C	T	LIN7A	85	18	-	-	-	-	-	nsSNV
22	42354487	42354489	GGA	G	LINC00634	34	8	-	-	-	-	-	ncRNA
15	66977723	66977723	G	T	LINC01169	69	5	-	-	-	-	-	ncRNA
19	42928360	42928360	G	A	LIPE-AS1	46	10	-	-	-	-	-	ncRNA
7	97823042	97823042	C	A	LMTK2	76	5	-	-	-	-	-	nsSNV
3	122606292	122606292	G	T	LOC100129550	73	23	-	-	-	-	-	ncRNA

20	25826811	25826811	C	G	LOC101926935	39	6	-	-	-	-	-	ncRNA
12	53447253	53447253	G	T	LOC283335	41	4	-	-	-	-	-	ncRNA
22	48934923	48934923	G	T	LOC284933	152	7	-	-	-	-	-	ncRNA
12	54520018	54520018	G	C	LOC400043	100	27	-	-	-	-	-	ncRNA
1	99771557	99771557	C	A	LPPR4	135	6	-	-	-	-	-	nsSNV
12	57548081	57548081	G	T	LRP1	179	7	-	-	-	-	-	nsSNV
12	57599434	57599434	A	T	LRP1	62	29	-	-	-	-	-	nsSNV
2	141609230	141609231	TC	T	LRP1B	45	27	-	-	-	-	-	FS del
20	6022609	6022609	C	A	LRRN4	83	5	-	-	-	-	-	nsSNV
3	54952849	54952849	A	T	LRTM1	40	18	-	-	-	-	-	sSNV
11	65306808	65306808	G	T	LTBP3	21	3	-	-	-	-	-	nsSNV
1	23418666	23418666	G	T	LUZP1	90	5	-	-	-	-	-	sSNV
6	6347040	6347040	T	A	LY86-AS1	93	5	-	-	-	-	-	ncRNA
6	10775640	10775640	G	T	MAK	50	5	-	-	-	-	-	sSNV
11	95825374	95825383	TTGCTGCTG	T	MAML2	24	57	-	-	O	-	-	IF del
9	139992324	139992324	C	A	MAN1B1	55	6	-	-	-	-	-	nsSNV
12	53881007	53881007	C	A	MAP3K12	56	5	-	-	-	-	-	nsSNV
X	20031173	20031173	C	A	MAP7D2	76	5	-	-	-	-	-	nsSNV
17	19285244	19285244	G	T	MAPK7	23	3	-	-	-	-	-	nsSNV
3	50679749	50679749	C	A	MAPKAPK3	24	4	-	-	-	-	-	nsSNV
18	32720314	32720314	C	A	MAPRE2	38	4	-	-	-	-	-	nsSNV
17	60814520	60814520	G	T	MARCH10	134	6	-	-	-	-	-	nsSNV
16	71668649	71668649	G	T	MARVELD3	32	4	-	-	-	-	-	nsSNV
20	3842045	3842045	C	A	MAVS	60	5	-	-	-	-	-	nsSNV
11	119182607	119182607	C	A	MCAM	62	4	-	-	-	-	-	nsSNV
3	127340071	127340071	G	A	MCM2	36	13	-	-	-	-	-	sSNV
6	90358006	90358006	G	T	MDN1	70	5	-	-	-	-	-	nsSNV
19	42865018	42865018	G	A	MEGF8	120	25	-	-	-	-	-	sSNV
7	141756685	141756685	G	T	MGAM	92	5	-	-	-	-	-	sSNV
1	62121191	62121192	TA	T	MGC34796	13	6	-	-	-	-	-	ncRNA
19	51321990	51321990	C	A	MGC45922	7	4	-	-	-	-	-	ncRNA
22	18273574	18273574	C	T	MICAL3	22	4	-	-	-	-	-	nsSNV
11	5011037	5011037	G	T	MMP26	69	5	-	-	-	-	-	nsSNV
14	61201592	61201592	G	T	MNAT1	45	4	-	-	-	-	-	nsSNV
1	113235503	113235503	C	A	MOV10	25	4	-	-	-	-	-	sSNV
17	56356929	56356929	G	T	MPO	87	5	-	-	-	-	-	nsSNV
8	142490048	142490048	G	A	MROH5	32	17	-	-	-	-	-	UN
20	35757529	35757529	G	A	MROH8	30	7	-	-	-	-	-	UN
11	66204369	66204369	G	A	MRPL11	60	12	-	-	-	-	-	nsSNV
7	100648065	100648065	C	T	MUC12	58	146	-	-	-	-	-	nsSNV
19	9076707	9076707	G	T	MUC16	87	5	-	-	-	-	-	nsSNV
11	1084822	1084822	G	T	MUC2	74	4	-	-	-	-	-	nsSNV
11	1016871	1016871	G	A	MUC6	154	11	-	-	-	-	-	nsSNV
11	1029572	1029572	G	T	MUC6	32	4	-	-	-	-	-	sSNV
22	36702009	36702009	G	T	MYH9	45	4	-	-	O	-	-	nsSNV
2	211167494	211167494	G	T	MYL1	39	20	-	-	-	-	-	nsSNV
17	18025208	18025208	C	A	MYO15A	69	5	-	-	-	-	-	nsSNV
17	73610680	73610680	C	A	MYO15B	40	5	-	-	-	-	-	ncRNA
13	109793496	109793496	C	A	MYO16	65	5	-	-	-	-	-	nsSNV
2	128387231	128387231	C	A	MYO7B	84	5	-	-	-	-	-	nsSNV
9	138903489	138903489	T	TC	NACC2	69	6	-	-	-	-	-	FS ins
16	5078940	5078940	C	A	NAGPA	85	6	-	-	-	-	-	sSNV
8	18080245	18080245	G	T	NAT1	108	7	-	-	-	-	-	nsSNV
12	78362412	78362412	C	A	NAV3	86	12	-	-	-	-	-	nsSNV
3	47033160	47033160	C	A	NBEAL2	70	5	-	-	-	-	-	nsSNV
11	113144386	113144386	C	T	NCAM1-AS1	47	13	-	-	-	-	-	ncRNA
11	134028302	134028302	C	A	NCAPD3	90	5	-	-	-	-	-	nsSNV
12	54902293	54902293	C	A	NCKAP1L	121	6	-	-	-	-	-	nsSNV
2	24952591	24952591	G	T	NCOA1	156	7	-	-	O	-	-	nsSNV
12	124826411	124826411	G	T	NCOR2	78	5	-	-	-	-	O	nsSNV
1	160322739	160322739	C	A	NCSTN	32	4	-	-	-	-	-	sSNV
14	21491046	21491046	G	T	NDRG2	36	3	-	-	-	-	-	sSNV
10	21101819	21101819	G	T	NEBL	32	4	-	-	-	-	-	sSNV
8	91804192	91804192	G	T	NECAB1	75	5	-	-	-	-	-	nsSNV
1	72241872	72241872	C	A	NEGR1	31	4	-	-	-	-	-	nsSNV
9	127088628	127088628	G	A	NEK6	72	35	-	-	-	-	-	nsSNV
17	46135741	46135741	C	A	NFE2L1	161	8	-	-	-	-	-	nsSNV
1	115828958	115828958	C	A	NGF	92	6	-	-	-	-	-	nsSNV

14	52473306	52473306	G	T	NID2	78	5	-	-	-	-	-	nsSNV
15	23006250	23006250	G	T	NIPA2	43	4	-	-	-	-	-	sSNV
8	23540374	23540374	C	A	NKX3-1	43	5	-	-	-	-	-	nsSNV
17	7319276	7319276	G	T	NLGN2	128	7	-	-	-	-	-	nsSNV
X	6069396	6069396	G	T	NLGN4X	86	5	-	-	-	-	-	nsSNV
19	56220344	56220344	G	T	NLRP9	87	5	-	-	-	-	-	sSNV
11	119052907	119052907	G	T	NLRX1	83	6	-	-	-	-	-	nsSNV
7	150711126	150711126	G	T	NOS3	93	5	-	-	-	-	-	nsSNV
X	100118486	100118486	C	A	NOX1	37	4	-	-	-	-	-	nsSNV
11	66192488	66192488	C	A	NPAS4	109	6	-	-	-	-	-	sSNV
3	132419238	132419238	C	A	NPHP3	87	5	-	-	-	-	-	sSNV
7	98256544	98256544	G	T	NPTX2	43	3	-	-	-	-	-	nsSNV
22	39239513	39239513	C	A	NPTXR	18	3	-	-	-	-	-	sSNV
16	67920001	67920003	ACT	A	NRN1L	97	63	-	-	-	-	-	FS del
11	64428417	64428417	C	A	NRXN2	95	5	-	-	-	-	-	nsSNV
10	104859696	104859696	C	A	NT5C2	106	7	-	-	O	-	-	nsSNV
12	104179244	104179244	G	T	NT5DC3	60	5	-	-	-	-	-	nsSNV
11	120097672	120097672	C	A	OAF	163	8	-	-	-	-	-	nsSNV
15	76019739	76019739	C	A	ODF3L1	77	5	-	-	-	-	-	nsSNV
3	9791994	9791994	C	A	OGG1	105	6	-	-	-	-	-	sSNV
11	132812898	132812898	G	T	OPCML	30	3	-	-	-	-	-	sSNV
12	48596213	48596213	G	T	OR10AD1	76	5	-	-	-	-	-	nsSNV
11	123908858	123908858	G	T	OR10G7	95	5	-	-	-	-	-	nsSNV
11	48346563	48346563	C	A	OR4C3	122	53	-	-	-	-	-	nsSNV
3	97869022	97869022	C	A	OR5H14	30	5	-	-	-	-	-	nsSNV
7	142750049	142750049	T	G	OR6V1	622	18	-	-	-	-	-	nsSNV
11	124180053	124180053	C	T	OR8D1	41	17	-	-	-	-	-	nsSNV
11	55904809	55904809	G	T	OR8J3	104	6	-	-	-	-	-	nsSNV
12	48920257	48920257	C	A	OR8S1	215	8	-	-	-	-	-	sSNV
12	58114219	58114219	G	T	OS9	43	4	-	-	-	-	-	nsSNV
3	161221576	161221576	C	T	OTOL1	15	5	-	-	-	-	-	nsSNV
12	56726729	56726729	G	T	PAN2	72	4	-	-	-	-	-	sSNV
16	50188133	50188133	C	A	PAPD5	38	4	-	-	-	-	-	nsSNV
19	39589134	39589134	G	T	PAPL	86	5	-	-	-	-	-	nsSNV
14	73733234	73733234	C	A	PAPLN	44	4	-	-	-	-	-	nsSNV
14	97000899	97000899	G	T	PAPOLA	142	6	-	-	-	-	-	nsSNV
20	49366736	49366736	C	A	PARD6B	142	7	-	-	-	-	-	nsSNV
3	122259629	122259629	C	A	PARP9	159	7	-	-	-	-	-	nsSNV
2	223066881	223066881	C	A	PAX3	46	4	-	-	O	-	-	nsSNV
3	52643508	52643508	C	A	PBRM1	144	7	-	-	O	O	O	nsSNV
11	66638627	66638627	G	T	PC	102	6	-	-	-	-	-	nsSNV
3	51993966	51993966	C	A	PCBP4	40	4	-	-	-	-	-	nsSNV
4	30724727	30724727	C	G	PCDH7	51	12	-	-	-	-	O	nsSNV
5	140209865	140209865	G	T	PCDHA6	56	20	-	-	-	-	-	nsSNV
5	140558897	140558897	G	T	PCDHB8	200	8	-	-	-	-	-	nsSNV
5	140711946	140711946	C	A	PCDHGA1	68	5	-	-	-	-	-	sSNV
20	17446037	17446037	C	T	PCSK2	256	24	-	-	-	-	-	sSNV
19	1487169	1487169	G	T	PCSK4	45	4	-	-	-	-	-	nsSNV
5	149498364	149498364	G	T	PDGFRB	151	7	-	-	O	-	-	sSNV
12	48535726	48535726	G	T	PFKM	188	11	-	-	-	-	-	nsSNV
3	169896685	169896685	G	T	PHC3	106	9	-	-	-	-	-	nsSNV
1	6680129	6680129	G	T	PHF13	38	4	-	-	-	-	-	nsSNV
18	60630608	60630608	C	A	PHLPP1	4	3	-	-	-	-	-	nsSNV
13	73409508	73409508	T	TA	PIBF1	1	9	-	-	-	-	-	splicing
3	138403499	138403499	G	T	PIK3CB	98	6	-	-	-	-	O	sSNV
1	46543247	46543247	C	T	PIK3R3	42	24	-	-	-	-	-	nsSNV
19	40873657	40873657	G	T	PLD3	81	4	-	-	-	-	-	sSNV
8	144992582	144992582	G	T	PLEC	35	4	-	-	-	-	-	nsSNV
8	144997967	144997967	G	T	PLEC	89	5	-	-	-	-	-	nsSNV
3	126746882	126746882	C	A	PLXNA1	148	7	-	-	-	-	-	nsSNV
1	208255771	208255771	G	T	PLXNA2	98	6	-	-	-	-	-	nsSNV
3	48457835	48457835	C	A	PLXNB1	21	3	-	-	-	-	-	nsSNV
1	166819553	166819553	A	T	POGK	50	36	-	-	-	-	-	nsSNV
22	42981894	42981894	G	T	POLDIP3	70	5	-	-	-	-	-	nsSNV
12	133233739	133233739	G	T	POLE	85	5	-	-	-	-	O	nsSNV
15	89871948	89871948	C	A	POLG	80	5	-	-	-	-	-	nsSNV
3	121168195	121168195	A	C	POLQ	74	27	-	-	-	-	-	nsSNV
22	38363139	38363139	G	A	POLR2F	13	13	-	-	-	-	-	sSNV

3	119378978	119378978	C	A	POPDC2	41	3	-	-	-	-	-	nsSNV
3	87325472	87325472	T	A	POU1F1	17	8	-	-	-	-	-	sSNV
6	99283522	99283522	C	A	POU3F2	82	5	-	-	-	-	-	nsSNV
2	44436479	44436479	C	G	PPM1B	93	38	-	-	-	-	-	nsSNV
11	61250211	61250211	C	A	PPP1R32	12	4	-	-	-	-	-	sSNV
12	108134927	108134927	G	T	PRDM4	174	7	-	-	-	-	-	nsSNV
X	23685907	23685907	C	A	PRDX4	30	4	-	-	-	-	-	nsSNV
13	39586310	39586310	C	A	PROSER1	26	3	-	-	-	-	-	sSNV
20	62614440	62614440	C	A	PRPF6	66	5	-	-	-	-	-	nsSNV
19	50098982	50098982	G	T	PRR12	39	5	-	-	-	-	-	nsSNV
9	134305561	134305561	G	T	PRRC2B	50	5	-	-	-	-	-	nsSNV
9	134351326	134351326	C	A	PRRC2B	164	10	-	-	-	-	-	sSNV
8	143763339	143763339	G	A	PSCA	86	25	rs376581990	rs376581990	-	-	-	nsSNV
19	43237008	43237008	C	T	PSG3	22	6	-	-	-	-	-	nsSNV
17	38151512	38151512	C	A	PSMD3	125	6	-	-	-	-	-	nsSNV
9	130885257	130885257	C	A	PTGES2	66	6	-	-	-	-	-	nsSNV
8	141678378	141678378	G	T	PTK2	150	7	-	-	-	-	-	nsSNV
6	43109749	43109749	G	A	PTK7	82	36	-	-	-	-	-	nsSNV
12	71139637	71139637	G	T	PTPRR	94	6	-	-	-	-	-	nsSNV
3	191179074	191179074	C	T	PYDC2	84	22	-	-	-	-	-	sSNV
16	640372	640372	G	T	RAB40C	44	4	-	-	-	-	-	nsSNV
X	103080708	103080708	C	A	RAB9B	35	4	-	-	-	-	-	nsSNV
20	20553610	20553610	C	T	RALGAPA2	26	7	-	-	-	-	-	nsSNV
3	50150859	50150859	G	T	RBM5	66	5	-	-	-	-	-	nsSNV
6	111696294	111696294	G	T	REV3L	80	5	-	-	-	-	-	sSNV
10	62648340	62648340	C	A	RHOBTB1	186	7	-	-	-	-	-	nsSNV
14	93118367	93118367	C	A	RIN3	86	5	-	-	-	-	-	nsSNV
1	40705314	40705314	G	T	RLF	92	5	-	-	-	-	-	nsSNV
8	101270957	101270957	G	T	RNF19A	95	6	-	-	-	-	-	nsSNV
14	24616983	24616983	G	T	RNF31	30	3	-	-	-	-	-	nsSNV
2	89037502	89037502	G	T	RPIA	32	4	-	-	-	-	-	sSNV
18	47015879	47015879	C	A	RPL17,RPL17-C1	26	4	-	-	-	-	-	sSNV
19	12936690	12936690	C	T	RTBDN	39	13	-	-	-	-	-	nsSNV
22	20229905	20229905	C	A	RTN4R	85	28	-	-	-	-	-	nsSNV
9	91616420	91616420	A	T	S1PR3	109	48	-	-	-	-	-	nsSNV
2	224463385	224463385	C	A	SCG2	93	5	-	-	-	-	-	nsSNV
7	12675689	12675689	C	A	SCIN	60	16	-	-	-	-	-	sSNV
11	118014739	118014739	G	T	SCN4B	90	6	-	-	-	-	-	nsSNV
2	167301483	167301483	G	T	SCN7A	92	5	-	-	-	-	-	nsSNV
11	9090981	9090981	C	A	SCUBE2	77	5	-	-	-	-	-	nsSNV
1	156146641	156146641	G	T	SEMA4A	75	5	-	-	-	-	-	sSNV
22	21138358	21138358	G	T	SERPIND1	136	6	-	-	-	-	-	nsSNV
16	30991124	30991124	C	A	SETD1A	35	4	-	-	-	-	-	sSNV
3	47098785	47098785	G	T	SETD2	128	6	-	-	O	O	O	sSNV
1	150915493	150915493	T	A	SETDB1	32	26	-	-	-	-	-	nsSNV
16	28884061	28884061	C	A	SH2B1	45	4	-	-	-	-	-	sSNV
19	51192455	51192455	C	A	SHANK1	14	3	-	-	-	-	-	nsSNV
19	422156	422156	G	T	SHC2	25	4	-	-	-	-	-	nsSNV
19	51920196	51920196	G	T	SIGLEC10	44	5	rs73049612	rs73049612	-	-	-	nsSNV
19	51645659	51645659	G	T	SIGLEC7	90	5	-	-	-	-	-	nsSNV
5	133502930	133502930	C	A	SKP1	83	5	-	-	-	-	-	nsSNV
1	160718048	160718048	C	A	SLAMF7	34	4	-	-	-	-	-	sSNV
16	56906304	56906304	C	A	SLC12A3	51	13	-	-	-	-	-	sSNV
3	124826587	124826587	C	A	SLC12A8	140	7	-	-	-	-	-	nsSNV
19	49933799	49933799	G	A	SLC17A7	16	4	-	-	-	-	-	nsSNV
5	36680612	36680612	C	A	SLC1A3	169	8	-	-	-	-	-	nsSNV
11	64329527	64329527	G	T	SLC22A11	82	5	-	-	-	-	-	sSNV
11	62763553	62763553	C	A	SLC22A8	171	8	-	-	-	-	-	nsSNV
7	87488025	87488025	C	A	SLC25A40	107	6	-	-	-	-	-	nsSNV
8	92378848	92378848	C	A	SLC26A7	83	5	-	-	-	-	-	nsSNV
15	45560541	45560541	G	A	SLC28A2	31	10	-	-	-	-	-	nsSNV
10	73111419	73111419	C	A	SLC29A3	39	4	-	-	-	-	-	nsSNV
6	44222724	44222724	G	T	SLC35B2	70	5	-	-	-	-	-	nsSNV
17	42335094	42335094	C	A	SLC4A1	38	4	-	-	-	-	-	nsSNV
20	3211679	3211679	C	A	SLC4A11	81	6	-	-	-	-	-	sSNV
3	27453191	27453191	C	T	SLC4A7	46	15	rs150040978	rs150040978	-	-	-	nsSNV
8	145584265	145584265	G	T	SLC52A2	90	5	-	-	-	-	-	sSNV
5	149578908	149578908	G	T	SLC6A7	131	7	-	-	-	-	-	nsSNV

12	21427436	21427436	C	A	SLCO1A2	159	7	-	-	-	-	-	nsSNV
4	20490610	20490610	G	T	SLIT2	33	4	-	-	-	-	-	nsSNV
10	105762531	105762531	G	T	SLK	58	5	-	-	-	-	-	nsSNV
20	57610099	57610099	C	T	SLMO2	20	12	-	-	-	-	-	nsSNV
19	11129675	11129675	C	A	SMARCA4	80	5	-	-	O	O	O	sSNV
4	90756714	90756714	C	A	SNCA	40	4	-	-	-	-	-	nsSNV
18	67992266	67992266	C	A	SOCS6	24	3	-	-	-	-	-	nsSNV
2	171573149	171573149	C	A	SP5	50	4	-	-	-	-	-	sSNV
13	24825897	24825897	C	A	SPATA13	81	6	-	-	-	-	-	sSNV
6	44320494	44320494	G	T	SPATS1	139	6	-	-	-	-	-	nsSNV
1	16254813	16254813	G	T	SPEN	84	5	-	-	-	-	O	nsSNV
1	16254891	16254891	G	A	SPEN	61	39	-	-	-	-	O	nsSNV
5	136320830	136320830	C	A	SPOCK1	109	6	-	-	-	-	-	nsSNV
17	43923312	43923312	G	T	SPPL2C	115	6	-	-	-	-	-	nsSNV
14	65239344	65239344	C	A	SPTB	34	4	-	-	-	-	-	nsSNV
14	65239665	65239665	C	A	SPTB	76	4	-	-	-	-	-	nsSNV
16	30724921	30724921	C	A	SRCAP	82	5	-	-	-	-	-	sSNV
7	104766715	104766715	C	A	SRPK2	24	3	-	-	-	-	-	nsSNV
22	26879946	26879967	GGAGGCGG	G	SRRD	69	27	-	-	-	-	-	IF del
22	37603027	37603027	G	T	SSTR3	80	5	-	-	-	-	-	sSNV
1	44303930	44303930	G	A	ST3GAL3	156	62	-	-	-	-	-	sSNV
7	116862001	116862001	C	A	ST7	35	4	-	-	-	-	-	nsSNV
8	74351277	74351277	G	T	STAU2-AS1	194	8	-	-	-	-	-	ncRNA
X	7177590	7177590	C	A	STS	84	5	-	-	-	-	-	nsSNV
20	57290463	57290463	C	A	STX16-NPEPL1	129	6	-	-	-	-	-	ncRNA
16	28603371	28603371	A	G	SULT1A2	15	9	-	-	-	-	-	nsSNV
16	28603372	28603372	G	T	SULT1A2	14	9	-	-	-	-	-	nsSNV
10	70968510	70968510	G	T	SUPV3L1	134	7	-	-	-	-	-	nsSNV
9	93636492	93636492	C	A	SYK	116	6	-	-	O	-	-	nsSNV
9	93641094	93641094	G	T	SYK	91	5	-	-	O	-	-	nsSNV
14	74876409	74876409	G	T	SYNDIG1L	17	4	-	-	-	-	-	sSNV
14	64678695	64678695	G	T	SYNE2	88	5	-	-	-	-	-	nsSNV
4	119948372	119948372	G	T	SYNPO2	62	5	-	-	-	-	-	nsSNV
1	43896734	43896734	C	A	SZT2	71	6	-	-	-	-	-	nsSNV
4	1729975	1729975	C	A	TACC3	19	3	-	-	-	-	-	sSNV
16	30002820	30002820	C	A	TAOK2	15	4	-	-	-	-	-	sSNV
1	234601455	234601456	CT	C	TARBP1	73	12	-	-	-	-	-	splicing
1	19180971	19180971	G	T	TAS1R2	76	5	-	-	-	-	-	sSNV
19	50390826	50390826	G	T	TBC1D17	81	5	-	-	-	-	-	nsSNV
11	12901388	12901388	G	T	TEAD1	8	3	-	-	-	-	-	nsSNV
5	167674392	167674392	C	A	TENM2	101	6	-	-	-	-	-	nsSNV
11	78440644	78440644	C	A	TENM4	38	4	-	-	-	-	-	sSNV
6	10415107	10415107	G	A	TFAP2A-AS1	18	5	-	-	-	-	-	ncRNA
1	92149414	92149414	C	A	TGFBR3	32	4	-	-	-	-	-	nsSNV
15	71175212	71175212	G	T	THAP10	86	6	-	-	-	-	-	sSNV
22	21354468	21354468	G	T	THAP7	39	4	-	-	-	-	-	nsSNV
X	122758005	122758005	C	A	THOC2	23	3	-	-	-	-	-	nsSNV
16	3074360	3074360	G	T	THOC6	82	6	-	-	-	-	-	sSNV
2	138329991	138329991	G	T	THSD7B	91	4	-	-	-	-	-	UN
8	144681483	144681483	G	T	TIGD5	92	5	-	-	-	-	-	sSNV
9	35708418	35708418	G	A	TLN1	3	8	-	-	-	-	-	nsSNV
4	38798572	38798572	G	T	TLR1	89	5	-	-	-	-	-	sSNV
20	2616601	2616601	C	G	TMC2	38	16	-	-	-	-	-	nsSNV
17	76109255	76109255	G	A	TMC6	29	5	-	-	-	-	-	sSNV
1	20009821	20009821	G	T	TMCO4	43	4	-	-	-	-	-	sSNV
12	57457942	57457942	C	A	TMEM194A	68	5	-	-	-	-	-	sSNV
9	140099861	140099861	G	T	TMEM203	27	4	-	-	-	-	-	sSNV
1	212550933	212550933	C	A	TMEM206	118	6	-	-	-	-	-	nsSNV
1	15443766	15443766	G	T	TMEM51-AS1	76	6	-	-	-	-	-	ncRNA
1	16069533	16069533	C	A	TMEM82	36	3	-	-	-	-	-	sSNV
15	52192437	52192437	C	A	TMOD3	53	29	-	-	-	-	-	nsSNV
17	26671453	26671453	C	A	TNFAIP1	38	4	-	-	-	-	-	nsSNV
17	18217952	18217952	C	A	TOP3A	25	5	-	-	-	-	-	nsSNV
1	179815593	179815593	C	A	TOR1AIP2	79	5	-	-	-	-	-	sSNV
9	123673617	123673617	G	T	TRAF1	12	3	-	-	-	-	-	nsSNV
6	116821669	116821669	C	T	TRAPPC3L	25	5	-	-	-	-	-	nsSNV
17	57141768	57141769	TA	T	TRIM37	43	7	-	-	-	-	-	splicing
3	160156679	160156679	G	T	TRIM59	94	6	-	-	-	-	-	nsSNV

11	8643262	8643262	T	A	TRIM66	49	25	-	-	-	-	-	nsSNV
5	14507230	14507230	G	T	TRIO	75	5	-	-	-	-	-	splicing
20	5921750	5921750	C	A	TRMT6	58	5	-	-	-	-	-	nsSNV
7	98562363	98562363	C	A	TRRAP	88	5	-	-	-	-	-	nsSNV
7	98576461	98576461	C	A	TRRAP	46	5	-	-	-	-	-	sSNV
7	100075040	100075040	G	T	TSC22D4	45	4	-	-	-	-	-	sSNV
20	51872809	51872809	C	A	TSHZ2	199	9	-	-	-	-	-	nsSNV
15	43044918	43044918	C	G	TTBK2	56	28	-	-	-	-	-	nsSNV
3	180328173	180328173	G	T	TTC14	38	5	-	-	-	-	-	nsSNV
17	40094865	40094865	G	T	TTC25	40	3	-	-	-	-	-	UN
2	179615173	179615173	G	T	TTN	43	4	-	-	-	-	-	nsSNV
2	179444880	179444880	G	T	TTN	91	6	-	-	-	-	-	sSNV
10	93711	93711	C	T	TUBB8	31	4	-	-	-	-	-	sSNV
10	135107202	135107202	C	A	TUBGCP2	42	5	-	-	-	-	-	nsSNV
1	154242713	154242713	C	A	UBAP2L	69	5	-	-	-	-	-	nsSNV
11	5529288	5529288	T	A	UBQLN3	30	4	-	-	-	-	-	nsSNV
11	5529289	5529289	C	A	UBQLN3	31	4	-	-	-	-	-	nsSNV
2	170897395	170897395	G	T	UBR3	136	8	-	-	-	-	-	sSNV
17	73838963	73838963	C	A	UNC13D	80	5	-	-	-	-	-	nsSNV
10	73051211	73051211	C	A	UNC5B	152	7	-	-	-	-	-	sSNV
2	210704103	210704103	C	A	UNC80	124	6	-	-	-	-	-	nsSNV
12	6971705	6971705	G	T	USP5	100	6	-	-	-	-	-	nsSNV
8	67563754	67563754	C	A	VCPIP1	126	6	-	-	-	-	-	nsSNV
2	219293043	219293043	C	A	VIL1	42	4	-	-	-	-	-	nsSNV
8	100883761	100883761	G	T	VPS13B	133	9	-	-	-	-	-	nsSNV
15	41192668	41192668	G	T	VPS18	80	5	-	-	-	-	-	nsSNV
11	124620732	124620732	C	A	VSIG2	38	4	-	-	-	-	-	nsSNV
10	50311832	50311832	G	T	VSTM4	29	4	-	-	-	-	-	sSNV
12	6076656	6076656	G	T	VWF	106	7	-	-	-	-	-	nsSNV
2	224746696	224746696	C	A	WDFY1	18	5	-	-	-	-	-	nsSNV
19	990330	990330	G	T	WDR18	104	7	-	-	-	-	-	nsSNV
2	128471517	128471517	T	C	WDR33	31	7	-	-	-	-	O	nsSNV
9	131396134	131396134	C	A	WDR34	64	4	-	-	-	-	-	sSNV
X	48933211	48933211	G	T	WDR45	19	4	-	-	-	-	-	nsSNV
19	33623327	33623327	G	T	WDR88	83	6	-	-	-	-	-	nsSNV
1	3553560	3553560	C	A	WRAP73	67	4	-	-	-	-	-	nsSNV
14	75245330	75245330	C	A	YLPM1	89	5	-	-	-	-	O	nsSNV
10	27420789	27420789	C	A	YME1L1	99	7	-	-	-	-	-	nsSNV
3	114058131	114058131	G	C	ZBTB20	80	6	-	-	-	-	-	sSNV
8	135621100	135621100	C	T	ZFAT	58	48	-	-	-	-	-	sSNV
10	81070801	81070804	CCTT	C	ZMIZ1	60	9	-	-	-	-	-	IF del
19	19790848	19790848	C	A	ZNF101	78	6	-	-	-	-	-	sSNV
19	44831632	44831632	G	T	ZNF112	78	5	-	-	-	-	-	nsSNV
19	58944746	58944746	C	A	ZNF132	172	8	-	-	-	-	-	nsSNV
16	3190790	3190790	C	A	ZNF213	18	3	-	-	-	-	-	sSNV
19	44680640	44680640	C	A	ZNF226	153	7	-	-	-	-	-	nsSNV
8	146201231	146201232	AC	A	ZNF252P	117	13	-	-	-	-	-	ncRNA
19	58452381	58452381	C	A	ZNF256	85	5	-	-	-	-	-	nsSNV
14	74371680	74371680	C	T	ZNF410	23	15	-	-	-	-	-	sSNV
5	121488780	121488780	G	T	ZNF474	294	10	-	-	-	-	-	stoploss
19	53015183	53015183	G	T	ZNF578	43	4	-	-	-	-	-	nsSNV
4	87034	87034	G	T	ZNF595	12	4	-	-	-	-	-	UN
16	2049619	2049619	C	T	ZNF598	71	5	rs369892816	rs369892816	-	-	-	nsSNV
3	40558080	40558080	G	T	ZNF620	81	6	-	-	-	-	-	nsSNV
16	31090723	31090723	G	T	ZNF646	90	5	-	-	-	-	-	nsSNV
19	53668299	53668299	C	T	ZNF665	108	23	-	-	-	-	-	nsSNV
8	146067311	146067311	G	T	ZNF7	113	6	-	-	-	-	-	sSNV
19	57755340	57755340	G	T	ZNF805	125	6	-	-	-	-	-	nsSNV
19	53056925	53056925	C	A	ZNF808	57	5	-	-	-	-	-	sSNV
16	71894454	71894454	G	A	ZNF821	108	8	-	-	-	-	-	nsSNV
16	71894455	71894455	C	G	ZNF821	107	8	-	-	-	-	-	sSNV
14	102792656	102792656	C	A	ZNF839	61	5	-	-	-	-	-	nsSNV
19	53855540	53855540	C	A	ZNF845	87	6	-	-	-	-	-	nsSNV
19	52888464	52888464	G	T	ZNF880	71	5	-	-	-	-	-	nsSNV
1	238053762	238053762	C	A	ZP4	117	6	-	-	-	-	-	nsSNV
1	45553675	45553675	C	A	ZSWIM5	116	6	-	-	-	-	-	nsSNV

*NonFlagged: dbSNP files subtracting Flagged dbSNP entries. Flagged SNPs include SNPs < 1% minor allele frequency (MAF) (or unknown), mapping only once to reference assembly, flagged in dbSnp as "clinically associated".
**nsSNV: non-synonymous SNV, sSNV: synonymous SNV, FS ins: frameshift insertion, FS del: frameshift deletion, IF ins: in-frame insertion, IF del: in-frame deletion, ncRNA: non-coding RNA, UN: Unknown. transcript maps to multiple locations, all as "coding transcripts", but none has a complete ORF.

Table S3. Processed WES data
(F) AYA10

Chr	Start	End	Ref	Alt	Altered gene	T_Ref	T_Alt	dbSNP138		CVE list			Mutation context**
								All	NonFlagged*	C	V	E	
20	47601265	47601265	G	C	ARFGEF2	44	5	-	-	-	-	-	splicing
12	58005746	58005746	C	T	ARHGEF25	44	31	-	-	-	-	-	sSNV
9	135762805	135762805	T	G	C9orf9	14	6	-	-	-	-	-	nsSNV
19	45912489	45912492	CAAG	C	CD3EAP	98	6	-	-	-	-	-	IF del
17	45234301	45234301	C	A	CDC27	21	3	-	-	-	-	O	nsSNV
17	45234707	45234707	T	A	CDC27	25	4	rs75353677	rs75353677	-	-	O	nsSNV
1	22902839	22902839	G	A	EPHA8	64	4	-	-	-	-	-	nsSNV
14	76905825	76905825	C	CG	ESRRB	46	7	-	-	-	-	-	FS ins
4	126369825	126369825	A	G	FAT4	137	5	-	-	-	-	-	nsSNV
15	51675998	51675998	C	T	GLDN	15	3	-	-	-	-	-	sSNV
4	3076665	3076665	A	AGCCGCCA(	HTT	19	7	-	-	-	-	-	IF ins
17	39412119	39412119	C	A	KRTAP9-9	49	5	rs77134625	rs77134625	-	-	-	nsSNV
17	39412093	39412093	C	A	KRTAP9-9	42	5	-	-	-	-	-	sSNV
11	95825371	95825374	CTGT	C	MAML2	43	13	-	-	O	-	-	IF del
11	95825362	95825362	C	T	MAML2	37	3	-	-	O	-	-	sSNV
12	5603608	5603608	G	GC	NTF3	77	8	-	-	-	-	-	FS ins
12	80665560	80665560	C	A	OTOGL	89	26	-	-	-	-	-	nsSNV
16	70154480	70154480	A	G	PDPR	26	3	rs200469748	rs200469748	-	-	-	nsSNV
20	9389295	9389295	A	T	PLCB4	121	8	-	-	-	-	-	nsSNV
1	3322119	3322119	G	GC	PRDM16	95	13	-	-	O	-	-	FS ins
12	15677895	15677895	A	C	PTPRO	78	6	-	-	-	-	-	nsSNV
22	42910199	42910199	G	A	RRP7A	51	6	rs3752505	rs146958654	-	-	-	nsSNV
5	77717632	77717632	A	T	SCAMP1	192	11	-	-	-	-	-	unknown
14	92920290	92920290	C	T	SLC24A4	64	6	-	-	-	-	-	sSNV
16	2816115	2816115	A	AC	SRRM2	455	26	-	-	-	-	-	FS ins
1	152083369	152083369	T	TC	TCHH	227	13	-	-	-	-	-	FS ins
16	67876787	67876796	GCAGCAGC/	G	THAP11	28	25	-	-	-	-	-	IF del
4	166964538	166964538	C	A	TLL1	67	5	-	-	-	-	O	nsSNV
7	43917136	43917136	A	AC	URGCP	157	10	-	-	-	-	-	FS ins

*NonFlagged: dbSNP files subtracting Flagged dbSNP entries. Flagged SNPs include SNPs < 1% minor allele frequency (MAF) (or unknown), mapping only once to reference assembly, flagged in dbSnp as "clinically associated".
**nsSNV: non-synonymous SNV, sSNV: synonymous SNV, FS ins: frameshift insertion, FS del: frameshift deletion, IF ins: in-frame insertion, IF del: in-frame deletion, ncRNA: non-coding RNA, UN: Unknown. transcript maps to multiple locations, all as "coding transcripts", but none has a complete ORF.
