# Supplementary material for: Clinical application of genomic profiling to find druggable targets for adolescent and young adult (AYA) cancer patients with metastasis
Source: BMC Cancer. 2016 Feb 29;16:170. doi: 10.1186/s12885-016-2209-1 (PMC4772349; doi:10.1186/s12885-016-2209-1)
Supplement: Supplementary file 5 — Patient-specific genetic alterations of TCGA AML study selected by our pattern-based annotation. (PDF 156 kb) [file 12885_2016_2209_MOESM5_ESM.pdf]

## Table S4. Patient-specific genetic alterations of TCGA AML study selected by our pattern-based annotation

ITALIC: BOTH TSG/OG

| Sample# | NEJM & Ours | NEJM only | Ours only |
|---|---|---|---|
| 2802 | DNMT3A (1), IDH1 (1), NPM1 (1), PTPN11 (3) | -- | -- |
| 2803 | -- | -- | -- |
| 2804 | PHF6 (1) | -- | -- |
| 2805 | IDH2 (1), RUNX1 (1) | -- | -- |
| 2806 | -- | -- | KDM6A (1) |
| 2807 | IDH2 (1), RUNX1 (3) | -- | ASXL1 (1), USP6 (3) |
| 2808 | CEBPA (3), NRAS (1) | -- | CSF3R (3) |
| 2809 | DNMT3A (1), NPM1 (1) | -- | -- |
| 2810 | IDH2 (1), NPM1 (1) | -- | -- |
| 2811 | DNMT3A (1), FLT3 (3), NPM1 (1), SMC1A (2) | -- | PTCH1 (1) |
| 2812 | FLT3 (3), NPM1 (1) | -- | -- |
| 2813 | TP53 (1) | -- | -- |
| 2814 | FLT3 (3) | -- | -- |
| 2816 | DNMT3A (1), FLT3 (3), NPM1 (1), NRAS (1) | -- | -- |
| 2817 | EZH2 (3), FAM5C (2) | -- | CBFB (1) |
| 2818 | DNMT3A (3), FLT3 (3), NPM1 (1), RAD21 (1) | -- | -- |
| 2819 | KIT (3) | -- | -- |
| 2820 | TP53 (1) | -- | SUZ12 (1) |
| 2821 | IDH1 (1), IDH2 (1), RUNX1 (1), U2AF1 (3) | -- | ASXL1 (1) |
| 2822 | DNMT3A (1), IDH1 (1), SMC1A (3), TET2 (1) | -- | MED12 (3) |
| 2823 | -- | -- | -- |
| 2824 | DNMT3A (3), NPM1 (1), SMC1A (2) | -- | -- |
| 2825 | DNMT3A (1), FLT3 (1), NPM1 (1) | -- | -- |
| 2826 | IDH2 (1), KRAS (1) , NPM1 (1) | -- | -- |
| 2827 | -- | -- | MYC (1) |
| 2828 | -- | -- | -- |
| 2829 | TP53 (1) | -- | -- |
| 2830 | DNMT3A (1), FLT3 (3), TET2 (1) | -- | -- |
| 2831 | DNMT3A (3) | -- | -- |
| 2832 | -- | -- | -- |
| 2833 | DNMT3A (1) | -- | CBFB (1) |
| 2834 | FLT3 (3) | -- | -- |
| 2835 | NPM1 (1) | -- | -- |
| 2836 | FLT3 (3), NPM1 (1) | -- | -- |
| 2837 | NPM1 (1) | -- | -- |
| 2838 | FAM5C (2/3), NRAS (1) | -- | -- |
| 2839 | CEBPA (1), DNMT3A (3), NPM1 (1), NRAS (1), PTPN11 (3), SMC3 (3), WT1 (1) | -- | -- |
| 2840 | FLT3 (3) | -- | -- |
| 2841 | -- | -- | -- |
| 2842 | -- | -- | -- |
| 2843 | KIT (1/3), U2AF1 (1) | -- | -- |
| 2844 | SMC1A (3), WT1 (1) | -- | -- |
| 2845 | CEBPA (1/3), TET2 (1) | -- | CAPRIN2 (3) |
| 2846 | KIT (3), WT1 (1) | -- | -- |
| 2847 | U2AF1 (1) | -- | -- |
| 2848 | NPM1 (1) | -- | -- |
| 2849 | -- | -- | KDM3B (2), THRAP3 (3) |
| 2850 | IDH2 (1), RUNX1 (1), STAG2 (1) | -- | NOTCH1 (1), SRSF2 (3) |
| 2851 | DNMT3A (1), FLT3 (3), SMC3 (1) | -- | -- |
| 2853 | DNMT3A (1), FLT3 (3), NPM1 (1), SMC3 (2) | -- | -- |
| 2854 | -- | -- | -- |
| 2855 | PHF6 (1), PTPN11 (1) | -- | -- |
| 2857 | TP53 (1) | -- | -- |
| 2858 | SMC1A (3) | -- | -- |
| 2859 | DNMT3A (1), HNRNPK (1), NPM1 (1) | -- | -- |
| 2860 | TP53 (1) | -- | -- |
| 2861 | DNMT3A (1), KRAS (1), NPM1 (1), U2AF1 (1) | -- | HDAC2 (2), TUSC3 (2) |
| 2862 | -- | -- | -- |
| 2863 | CEBPA (3), DNMT3A (1), IDH1 (1) | -- | EGFR (3) |
| 2864 | DNMT3A (1), IDH2 (1), KRAS (1) | -- | ASXL1 (1) |
| 2865 | EZH2 (3), KRAS (1), RUNX1 (1/3), TET2 (1) | -- | JAK2 (1) |
| 2866 | IDH2 (1) | -- | -- |
| 2867 | DNMT3A (3), IDH1 (1) | -- | KDM6A (3) |
| 2868 | TP53 (1) | -- | -- |
| 2869 | DNMT3A (1), FLT3 (1), NPM1 (1) | -- | PAX3 (2) |
| 2870 | FLT3 (3), NRAS (1) | -- | -- |
| 2871 | FLT3 (3), NPM1 (1), PTPN11 (1), STAG2 (1) | -- | ELF4 (2), THRAP3 (1) |
| 2872 | -- | -- | TCL1A (3) |
| 2873 | DNMT3A (1), TET2 (1) | -- | -- |
| 2874 | CEBPA (3), IDH2 (1), WT1 (1) | -- | SMG1 (3) |
| 2875 | FLT3 (3) | -- | -- |
| 2876 | TET2 (1) | -- | -- |
| 2877 | FLT3 (3), IDH2 (1), NPM1 (1) | -- | -- |
| 2878 | TP53 (1) | -- | EGFR (1) |
| 2879 | FLT3 (1), NPM1 (1), TET2 (1) | -- | -- |
| 2880 | CEBPA (1), FLT3 (1) | -- | -- |
| 2881 | KIT (3) | -- | -- |
| 2882 | U2AF1 (1) | -- | SMG1 (3) |
| 2883 | -- | -- | KDM6A (1) |
| 2884 | DNMT3A (1), IDH1 (1), NPM1 (1), PTPN11 (1) | -- | -- |
| 2885 | PTPN11 (1), TP53 (1) | -- | -- |

| | | | |
|---|---|---|---|
| 2886 | RAD21 (1) | -- | JAK3 (1) |
| 2887 | DNMT3A (3), EZH2 (3), IDH1 (1), NRAS (1) | -- | -- |
| 2888 | KIT (3) | -- | -- |
| 2889 | -- | -- | -- |
| 2890 | RUNX1 (1) | -- | -- |
| 2891 | DNMT3A (1/3), IDH2 (1) | -- | -- |
| 2892 | NRAS (1) | -- | -- |
| 2893 | -- | -- | -- |
| 2894 | -- | -- | -- |
| 2895 | DNMT3A (1), FLT3 (1), NPM1 (1) | -- | PRDM16 (3) |
| 2896 | DNMT3A (1), NPM1 (1) | -- | -- |
| 2897 | -- | -- | MLLT4 (3) |
| 2898 | DNMT3A (1/3), IDH2 (1) | -- | -- |
| 2899 | RUNX1 (3) | -- | SETBP1 (1) |
| 2900 | CEBPA (1), FLT3 (3), NPM1 (1), SMC1A (2) | -- | NOTCH2 (3) |
| 2901 | IDH1 (1) | -- | -- |
| 2903 | NPM1 (1) | -- | -- |
| 2904 | TP53 (1) | -- | -- |
| 2905 | WT1 (1) | -- | CSMD1 (1) |
| 2906 | FLT3 (3) | -- | IKZF1 (3) |
| 2907 | IDH2 (1), RUNX1 (1) | -- | ASXL1 (1) |
| 2908 | DNMT3A (1), SMC3 (3), TET2 (1), TP53 (1) | -- | ARIDI1A (3), KDM3B (1) |
| 2909 | FLT3 (3) | -- | -- |
| 2910 | FLT3 (3) | -- | CIC (3) |
| 2911 | -- | -- | -- |
| 2912 | DNMT3A (1), PHF6 (1), RUNX1 (3), SMC3 (3), U2AF1 (1) | -- | -- |
| 2913 | FLT3 (3), NPM1 (1), STAG2 (1), WT1 (1) | -- | -- |
| 2914 | PTPN11 (1) | -- | CBL (1) |
| 2915 | FLT3 (3), NPM1 (3) | -- | -- |
| 2916 | DNMT3A (1) | -- | CSMD1 (3), MYB (3) |
| 2917 | KRAS (1) | -- | MED12 (1), SETBP1 (3) |
| 2918 | FLT3 (3) | -- | -- |
| 2919 | DNMT3A (3), IDH1 (1), NPM1 (1), WT1 (3) | -- | -- |
| 2920 | -- | -- | NF1 (1) |
| 2921 | FLT3 (3) | -- | -- |
| 2922 | FLT3 (3) | -- | -- |
| 2923 | NRAS (1), TET2 (1) | -- | -- |
| 2924 | FLT3 (3), NPM1 (1) | -- | -- |
| 2925 | DNMT3A (1), FLT3 (3), NPM1 (1) | -- | CSMD1 (3), GATA2 (3) |
| 2926 | FLT3 (3), IDH1 (1) | -- | -- |
| 2927 | RUNX1 (1/3) | -- | ASXL1 (1) |
| 2928 | DNMT3A (3), FLT3 (1), IDH1 (1), TET2 (1/3) | -- | -- |
| 2929 | KRAS (1) | -- | SF3B1 (1) |
| 2930 | FLT3 (3), WT1 (1) | -- | -- |
| 2931 | DNMT3A (1), FLT3 (3), NPM1 (1) | -- | -- |
| 2932 | NPM1 (1), NRAS (1) | -- | SETD2 (1) |
| 2933 | RUNX1 (3) | -- | -- |
| 2934 | DNMT3A (3), FLT3 (3), IDH2 (1) | -- | -- |
| 2935 | TP53 (1) | -- | -- |
| 2936 | IDH2 (1), RUNX1 (1) | -- | -- |
| 2937 | HNRNPK (1), KIT (3), TET2 (1) | -- | -- |
| 2938 | DNMT3A (3), TP53 (1) | -- | -- |
| 2939 | KIT (3) | -- | -- |
| 2940 | CEBPA (1/3) | -- | -- |
| 2941 | TP53 (1) | -- | -- |
| 2942 | FLT3 (3) | -- | -- |
| 2943 | TP53 (1) | -- | -- |
| 2945 | DNMT3A (1), FLT3 (3), IDH1 (1), KIT (3), NPM1 (1) | -- | -- |
| 2946 | -- | -- | -- |
| 2947 | DNMT3A (1), FLT3 (3), NPM1 (1) | -- | -- |
| 2948 | IDH2 (1) | -- | -- |
| 2949 | DNMT3A (1), IDH1 (1), RUNX1 (1) | -- | SUZ12 (1) |
| 2950 | SMC3 (1) | -- | ARHGAP35 (3) |
| 2952 | CEBPA (1), NRAS (1), TP53 (1) | -- | *FAT1 (3)* , MDM2 (3) |
| 2954 | -- | -- | -- |
| 2955 | CEBPA (1/3), DNMT3A (1) | -- | GATA2 (3) |
| 2956 | -- | -- | -- |
| 2957 | FLT3 (3) | -- | -- |
| 2959 | IDH2 (1), PHF6 (1), RUNX1 (1) | -- | -- |
| 2963 | FLT3 (3), NPM1 (1), PTPN11 (1), SMC1A (3) | -- | -- |
| 2964 | STAG2 (1), TET2 (1/3) | -- | CAMTA1 (3) |
| 2965 | DNMT3A (1), FAM5C (3), FLT3 (3), NPM1 (1), TET2 (1) | -- | -- |
| 2966 | DNMT3A (3), IDH2 (1), KRAS (1) | -- | SMG1 (2) |
| 2967 | DNMT3A (3), NPM1 (1), NRAS (1), RAD21 (1) | -- | -- |
| 2968 | DNMT3A (1/3), NRAS (1), U2AF1 (1) | -- | BCOR (1) |
| 2969 | DNMT3A (1), FLT3 (3), IDH1 (1), NPM1 (1) | -- | -- |
| 2970 | FLT3 (3), RUNX1 (1), WT1 (1) | -- | BCOR (1) |
| 2971 | TET2 (1/3) | -- | -- |
| 2972 | NPM1 (1), PTPN11 (1), STAG2 (1) | -- | -- |
| 2973 | IDH2 (1), NPM1 (1) | -- | -- |
| 2974 | DNMT3A (1), FLT3 (3), NPM1 (1) | -- | -- |
| 2975 | DNMT3A (1), RAD21 (1) | -- | -- |
| 2976 | FAM5C (3), FLT3 (3), NPM1 (1), PHF6 (3), WT1 (1) | -- | -- |

| | | | |
|---|---|---|---|
| 2977 | IDH1 (1) | -- | -- |
| 2978 | NRAS (1), RUNX1 (1), STAG2 (1), TET2 (1) | -- | -- |
| 2979 | CEBPA (1/3) | -- | -- |
| 2980 | FLT3 (3) | -- | -- |
| 2981 | DNMT3A (1), FLT3 (3), NPM1 (1) | -- | -- |
| 2982 | -- | -- | -- |
| 2983 | RUNX1 (3) | -- | -- |
| 2984 | IDH1 (1), NPM1 (1), NRAS (1) | -- | -- |
| 2985 | NRAS (1) | -- | -- |
| 2986 | FLT3 (1), NPM1 (1), RAD21 (1) | -- | -- |
| 2987 | DNMT3A (1), KRAS (1), NPM1 (1) | -- | PDGFRA (3) |
| 2988 | DNMT3A (3), NPM1 (1) | -- | -- |
| 2989 | NPM1 (1), WT1 (1) | -- | CBL (3) |
| 2990 | IDH1 (1), NPM1 (1) | -- | -- |
| 2991 | -- | -- | ARID2 (1), MAX (1) |
| 2992 | IDH1 (1), NPM1 (1) | -- | -- |
| 2993 | DNMT3A (3), FLT3 (3), NPM1 (1), SMC3 (3) | -- | -- |
| 2994 | FLT3 (3) | -- | -- |
| 2995 | -- | -- | -- |
| 2996 | TET2 (1/3), U2AF1 (1) | -- | MPL (3) |
| 2997 | -- | -- | -- |
| 2998 | FAM5C (2), FLT3 (3) | -- | -- |
| 2999 | -- | -- | ABL1 (3) |
| 3000 | CEBPA (1/3) | -- | -- |
| 3001 | -- | -- | -- |
| 3002 | IDH2 (1) | -- | -- |
| 3005 | -- | -- | -- |
| 3006 | FLT3 (1), TET2 (1) | -- | -- |
| 3007 | FLT3 (3) | -- | -- |
| 3008 | CEBPA (3) | -- | -- |
| 3009 | PHF6 (3), RUNX1 (1), WT1 (1) | -- | CTCF (1), DAXX (3), NF1 (1), SUZ12 (3) |
| 3011 | IDH1 (1), NPM1 (1) | -- | -- |
| 3012 | -- | -- | -- |
