# Supplementary material for: Clinical application of genomic profiling to find druggable targets for adolescent and young adult (AYA) cancer patients with metastasis
Source: BMC Cancer. 2016 Feb 29;16:170. doi: 10.1186/s12885-016-2209-1 (PMC4772349; doi:10.1186/s12885-016-2209-1)
Supplement: Supplementary file 6 — Candidate driving genetic alterations of AYA cancers. (PDF 150 kb) [file 12885_2016_2209_MOESM6_ESM.pdf]

Table S5. Candidate driving genetic alterations of AYA cancers

No. AYA	Cancer Type	Oncogenic type	Altered gene	Mutation type	Allele Freq.	Protein change	R†	Class	Level
**#01**	**Prostate cancer**	M	ATAD5	nsSNV	0.12	G451D	0	C2	Lv3 TSG
			NF1	FS del	0.17	S1561fs‡	0	C1	Lv1 TSG
			NF1	LOH	-	-	-	CNV	Lv1 TSG
			RASA2	splicing	0.09	A742_splice	0	C2	Lv1 TSG
			RASA2	FS ins	0.09	D282fs	0	C2	Lv1 TSG
			SUZ12	LOH	-	-	-	CNV	Lv2 TSG
**#02**	**Olfactory neuroblastoma**	C	BRINP1	IF ins	0.58	681_682insT	0	C2	Lv3 TSG
			CARD11	FS ins	0.44	Y609fs	0	C1	Lv3 OG
			CDKN2C	nonsense	0.67	Q26*‡	0	C1	Lv1 TSG
			MEIS1	FS ins	0.08	R102fs	0	C2	Lv3 OG
			MINK1	nsSNV	0.64	E837K‡	0	C2	Lv3 TSG
			PPP6C	splicing	0.15	G80_splice‡	0	C2	Lv1 TSG
			TGFBR2	nsSNV	0.08	R49S	0	C2	Lv3 TSG/Lv3 OG
			TP53	nsSNV	0.57	M237V‡	>30	C1	Lv1 TSG
**#04**	**Head and neck squamous cell carcinoma**	C	ANK2	nsSNV	0.16	H2013Y	0	C2	Lv3 TSG
			AXIN1	LOH	-	-	-	CNV	Lv2 TSG
			BAP1	LOH	-	-	-	CNV	Lv2 TSG
			CDH1	LOH	-	-	-	CNV	Lv2 TSG
			CHD5	nsSNV	0.17	R1891W	0	C2	Lv3 TSG
			FAT1	FS ins	0.20	L3696fs	-	C2	Lv1 TSG/Lv3 OG
			FOXL2	nsSNV	0.30	P157A	0	C1	Lv3 OG
			ITGB4	nsSNV	0.10	E839V‡	0	C2	Lv3 OG
			LECT1	nsSNV	0.10	W325G‡	0	C2	Lv3 TSG
			MSX1	nsSNV	0.09	S208L	1	C2	Lv2 TSG
			NOTCH1	Focal del	-	-	-	CNV	Lv1 TSG
			NOTCH1	LOH	-	-	-	CNV	Lv1 TSG
			PTCH1	LOH	-	-	-	CNV	Lv2 TSG
			SETD2	Focal del	-	-	-	CNV	Lv2 TSG
			SETD2	LOH	-	-	-	CNV	Lv2 TSG
			SMAD4	Focal del	-	-	-	CNV	Lv1 TSG
			TP53	splicing	0.28	S33_splice‡	>5	C1	Lv1 TSG
			USP6	nsSNV	0.05	R133K	2	C2	Lv2 OG
**#06**	**Urachal carcinoma**	M	CDH1	LOH	-	-	-	CNV	Lv2 TSG
			KRAS	nsSNV	0.07	G13D‡	>100	C1	Lv1 OG
			USP6	nsSNV	0.08	R133K	2	C2	Lv2 OG
**#09**	**Lung cancer**	NA§	EML4-ALK	Fusion	-	-	-	-	OG
**#10**	**Liposarcoma**	C	MDM2	Focal amp	-	-	-	CNV	Lv1 OG
			PRDM16	FS ins	0.12	A365fs‡	1	C2	Lv2 OG
			PTEN	Focal del	-	-	-	CNV	Lv1 TSG
			PTPRO	nsSNV	0.07	K680T‡	1	C2	Lv2 TSG
			URGCP	FS ins	0.06	F643fs‡	1	C2	Lv2 OG

*stop codon
†recurrence by cBio portal
‡ more than one NM number
§ not applicable
