# Supplementary material for: Clinical application of genomic profiling to find druggable targets for adolescent and young adult (AYA) cancer patients with metastasis
Source: BMC Cancer. 2016 Feb 29;16:170. doi: 10.1186/s12885-016-2209-1 (PMC4772349; doi:10.1186/s12885-016-2209-1)
Supplement: Supplementary file 7 — RasGAPs in large-scale studies. (PDF 234 kb) [file 12885_2016_2209_MOESM7_ESM.pdf]

Table S6. RasGAPs in large-scale studies

Analysis date: 2015-03-11

Uterine Corpus Endometrial Carcinoma (TCGA, Provisional)/n=248

Mutation rate: 18.5%

Gene	RASA1	NF1	RASAL2	RASAL1	RASA2	SYNGAP1	DAB2IP	RASAL3	RASA3	RASA4
**RASA1**	---	<0.0001	<0.0001	0.012156	<0.0001	0.001146	NS	NM	NS	NM
**NF1**		---	<0.0001	0.001128	<0.0001	<0.0001	0.007734	NM	NS	NM
**RASAL2**			---	0.003268	0.00015	0.000278	0.044567	NM	0.030791	NM
**RASAL1**				---	0.00088	0.002741	0.001611	NM	0.000828	NM
**RASA2**					---	0.002082	<0.0001	NM	NS	NM
**SYNGAP1**						---	0.009737	NM	NS	NM
**DAB2IP**							---	NM	NS	NM
**RASAL3**								---	NM	NM
**RASA3**									---	NM
**RASA4**										---

Uterine Corpus Endometrial Carcinoma (TCGA, Nature 2013)/n=248

Mutation rate: 18.5%

Gene	RASA1	NF1	RASAL2	RASAL1	RASA2	SYNGAP1	DAB2IP	RASAL3	RASA3	RASA4
**RASA1**	---	<0.0001	<0.0001	0.012156	<0.0001	0.001146	NS	NM	NS	NM
**NF1**		---	<0.0001	0.001128	<0.0001	<0.0001	0.007734	NM	NS	NM
**RASAL2**			---	0.003268	0.00015	0.000278	0.044567	NM	0.030791	NM
**RASAL1**				---	0.00088	0.002741	0.001611	NM	0.000828	NM
**RASA2**					---	0.002082	<0.0001	NM	NS	NM
**SYNGAP1**						---	0.009737	NM	NS	NM
**DAB2IP**							---	NM	NS	NM
**RASAL3**								---	NM	NM
**RASA3**									---	NM
**RASA4**										---

Colorectal Adenocarcinoma (TCGA, Nature 2012)/n=224

Mutation rate: 12.5%

Gene	NF1	RASA3	RASAL2	RASA1	DAB2IP	SYNGAP1	RASA2	RASAL1	RASAL3	RASA4
**NF1**	---	NS	0.021493	0.000576	NS	0.010615	0.003303	NS	NM	NM
**RASA3**		---	NS	0.01187	NS	0.008034	NS	0.000841	NM	NM
**RASAL2**			---	NS	NS	NS	NS	NS	NM	NM
**RASA1**				---	NS	0.005792	0.00178	0.000601	NM	NM
**DAB2IP**					---	NS	NS	NS	NM	NM
**SYNGAP1**						---	0.00119	0.044242	NM	NM
**RASA2**							---	0.026666	NM	NM
**RASAL1**								---	NM	NM
**RASAL3**									---	NM
**RASA4**										---

Pancreatic Adenocarcinoma (TCGA, Provisional)/n=91

Mutation rate: 24.2%

Gene	SYNGAP1	DAB2IP	RASAL1	RASAL2	NF1	RASA1	RASA2	RASA3	RASA4	RASAL3
**SYNGAP1**	---	NS	NS	NS	NS	NS	NS	NS	NS	NM
**DAB2IP**		---	NS	NS	NS	NS	NS	NS	NS	NM
**RASAL1**			---	0.011	0.022	0.022	0.022	NS	NS	NM
**RASAL2**				---	0.022	0.022	0.022	NS	NS	NM
**NF1**					---	0.011	0.011	NS	NS	NM
**RASA1**						---	0.011	NS	NS	NM
**RASA2**							---	NS	NS	NM
**RASA3**								---	NS	NM
**RASA4**									---	NM
**RASAL3**										---

Skin Cutaneous Melanoma (Broad, Cell 2012)/n=121

Mutation rate: 30.6%

Gene	NF1	RASAL1	RASAL2	RASA2	RASA3	RASAL3	SYNGAP1	DAB2IP	RASA1	RASA4
**NF1**	---	0.017543	NS	0.016364	NS	NM	NS	NS	NS	NM
**RASAL1**		---	NS	NS	NS	NM	NS	NS	NS	NM
**RASAL2**			---	NS	NS	NM	NS	NS	NS	NM
**RASA2**				---	NS	NM	NS	NS	NS	NM
**RASA3**					---	NM	NS	NS	NS	NM
**RASAL3**						---	NM	NM	NM	NM
**SYNGAP1**							---	NS	NS	NM
**DAB2IP**								---	NS	NM
**RASA1**									---	NM
**RASA4**										---

Skin Cutaneous Melanoma (TCGA, Provisional)/n=345

Mutation rate: 24.9%

Gene	NF1	RASAL2	RASA2	RASAL1	DAB2IP	RASAL3	SYNGAP1	RASA3	RASA1	RASA4
**NF1**	---	0.000532	0.000313	NS	NS	NM	0.045162	0.000026	NS	NS
**RASAL2**		---	0.000491	0.002217	NS	NM	NS	0.041531	NS	NS
**RASA2**			---	NS	NS	NM	NS	0.002573	NS	NS
**RASAL1**				---	NS	NM	NS	NS	NS	NS
**DAB2IP**					---	NM	NS	NS	NS	NS
**RASAL3**						---	NM	NM	NM	NM
**SYNGAP1**							---	0.01782	NS	NS
**RASA3**								---	NS	0.040226
**RASA1**									---	NS
**RASA4**										---

Skin Cutaneous Melanoma (Yale, Nature Genetics 2012)/n=91

Mutation rate: 24.2%

Gene	NF1	RASAL2	RASAL1	DAB2IP	RASA2	RASA1	RASA3	RASA4	RASAL3	SYNGAP1
**NF1**	---	NS	NS	NS	NS	NS	NS	NM	NM	NM
**RASAL2**		---	NS	NS	NS	NS	NS	NM	NM	NM
**RASAL1**			---	NS	NS	NS	NS	NM	NM	NM
**DAB2IP**				---	NS	NS	NS	NM	NM	NM
**RASA2**					---	NS	NS	NM	NM	NM
**RASA1**						-	0.010989	NM	NM	NM
**RASA3**							---	NM	NM	NM
**RASA4**								---	NM	NM
**RASAL3**									---	NM
**SYNGAP1**										---

Lung Adenocarcinoma (Broad, Cell 2012)/n=183 (WES=159, WES&WGS=23, WGS=1)

Mutation rate: 18.0%

Gene	NF1	RASAL3	RASA1	RASA3	RASAL2	RASAL1	SYNGAP1	DAB2IP	RASA2	RASA4
**NF1**	---	NM	NS	NS	NS	0.032	NS	0.011	NS	NM
**RASAL3**		---	NM	NM	NM	NM	NM	NM	NM	NM
**RASA1**			---	NS	NS	NS	NS	0.043	NS	NM
**RASA3**				---	NS	NS	NS	NS	0.043	NM
**RASAL2**					---	NS	NS	NS	NS	NM
**RASAL1**						---	NS	NS	NS	NM
**SYNGAP1**							---	NS	NS	NM
**DAB2IP**								---	NS	NM
**RASA2**									---	NM
**RASA4**										---

Lung Adenocarcinoma (TCGA, Nature 2014)/n=230

Mutation rate: 22.6%

Gene	NF1	RASAL2	RASAL1	RASA3	RASA1	SYNGAP1	RASA2	DAB2IP	RASAL3	RASA4
**NF1**	---	NS	NS	NS	NS	NS	NS	NS	NM	NM
**RASAL2**		---	NS	NS	NS	NS	NS	NS	NM	NM
**RASAL1**			---	NS	NS	NS	NS	NS	NM	NM
**RASA3**				---	NS	NS	NS	NS	NM	NM
**RASA1**					---	NS	NS	NS	NM	NM
**SYNGAP1**						---	NS	NS	NM	NM
**RASA2**							---	NS	NM	NM
**DAB2IP**								---	NM	NM
**RASAL3**									---	NM
**RASA4**										---

Lung Adenocarcinoma (TCGA, Provisional)/n=229

Mutation rate: 22.7%

Gene	NF1	RASAL2	RASAL1	RASA3	RASA1	SYNGAP1	RASA2	DAB2IP	RASAL3	RASA4
**NF1**	---	NS	NS	NS	NS	NS	NS	NS	NM	NS
**RASAL2**		---	0.026	<0.001	NS	0.013	NS	NS	NM	NS
**RASAL1**			---	0.011	NS	0.008	NS	NS	NM	NS
**RASA3**				---	NS	0.006	NS	NS	NM	NS
**RASA1**					---	NS	NS	NS	NM	NS
**SYNGAP1**						---	NS	NS	NM	NS
**RASA2**							---	NS	NM	NS
**DAB2IP**								---	NM	NS
**RASAL3**									---	NM
**RASA4**										---

Stomach Adenocarcinoma (TCGA, Provisional)/n=221

Mutation rate: 23.1%

Gene	NF1	RASAL2	RASA1	SYNGAP1	RASA3	DAB2IP	RASA2	RASAL1	RASAL3	RASA4
**NF1**	---	NS	NS	NS	NS	NS	NS	NS	NM	NM
**RASAL2**		---	NS	NS	NS	NS	NS	NS	NM	NM
**RASA1**			---	NS	NS	NS	NS	NS	NM	NM
**SYNGAP1**				---	NS	NS	NS	NS	NM	NM
**RASA3**					---	NS	NS	NS	NM	NM
**DAB2IP**						---	NS	NS	NM	NM
**RASA2**							---	NS	NM	NM
**RASAL1**								---	NM	NM
**RASAL3**									---	NM
**RASA4**										---

Stomach Adenocarcinoma (Pfizer and UHK, Nature Genetics 2014)/n=100

Mutation rate: 11.0%

Gene	NF1	RASAL2	RASA1	SYNGAP1	RASA3	DAB2IP	RASA2	RASAL1	RASAL3	RASA4
**NF1**	---	0.017204	NS	NS	NM	NS	NS	NS	NM	NM
**RASAL2**		---	NS	0.04	NM	NS	0.04	NS	NM	NM
**RASA1**			---	NS	NM	NS	NS	NS	NM	NM
**SYNGAP1**				---	NM	NS	NS	NS	NM	NM
**RASA3**					---	NM	NM	NM	NM	NM
**DAB2IP**						---	0.02	NS	NM	NM
**RASA2**							---	NS	NM	NM
**RASAL1**								---	NM	NM
**RASAL3**									---	NM
**RASA4**										---

Stomach Adenocarcinoma (TCGA, Nature 2014)/n=289

Mutation rate: 24.2%

Gene	NF1	RASAL2	RASA1	SYNGAP1	RASA3	DAB2IP	RASA2	RASAL1	RASAL3	RASA4
**NF1**	---	NS	0.047997	0.00648	NS	NS	0.024057	NS	NM	NM
**RASAL2**		---	NS	0.01096	NS	NS	NS	NS	NM	NM
**RASA1**			---	NS	NS	NS	NS	NS	NM	NM
**SYNGAP1**				---	NS	NS	0.027052	NS	NM	NM
**RASA3**					---	NS	NS	NS	NM	NM
**DAB2IP**						---	NS	0.001332	NM	NM
**RASA2**							---	0.01317	NM	NM
**RASAL1**								---	NM	NM
**RASAL3**									---	NM
**RASA4**										---

Cervical Squamous Cell Carcinoma and Endocervical Adenocarcinoma (TCGA, Provisional)/n=194
Mutation rate: 10.3%

Gene	NF1	RASAL3	RASA1	RASA3	RASAL2	RASAL1	SYNGAP1	DAB2IP	RASA2	RASA4
NF1	---	NM	NS	NS	NS	NS	0.004	NM	NS	NS
RASAL3		---	NM	NM	NM	NM	NM	NM	NM	NM
RASA1			---	NS	NS	NS	NS	NM	NS	NS
RASA3				---	NS	NS	0.046	NM	NS	NS
RASAL2					---	NS	NS	NM	NS	0.01
RASAL1						---	NS	NM	NS	NS
SYNGAP1							---	NM	NS	NS
DAB2IP								---	NM	NM
RASA2									---	NS
RASA4										---

Lung Squamous Cell Carcinoma (TCGA, Nature 2012)/n=178

Mutation rate: 18.0%

Gene	NF1	RASA1	RASAL2	RASAL1	RASAL3	SYNGAP1	RASA2	RASA3	DAB2IP	RASA4
**NF1**	---	NS	NS	0.031583	NM	NS	NS	NS	NS	NM
**RASA1**		---	NS	NS	NM	NS	NS	NS	NS	NM
**RASAL2**			---	NS	NM	NS	NS	NS	NS	NM
**RASAL1**				---	NM	NS	0.044563	NS	NS	NM
**RASAL3**					---	NM	NM	MN	NM	NM
**SYNGAP1**						---	NS	NS	NS	NM
**RASA2**							---	NS	NS	NM
**RASA3**								---	NS	NM
**DAB2IP**									---	NM
**RASA4**										---

Lung Squamous Cell Carcinoma (TCGA, Provisional)/n=178

Mutation rate: 24.7%

Gene	NF1	RASA1	RASAL2	RASAL1	RASAL3	SYNGAP1	RASA2	RASA3	DAB2IP	RASA4
**NF1**	---	NS	NS	0.036557	NM	NS	NS	NS	NS	NM
**RASA1**		---	NS	NS	NM	NS	NS	NS	NS	NM
**RASAL2**			---	NS	NM	NS	NS	NS	NS	NM
**RASAL1**				---	NM	NS	0.003923	NS	NS	NM
**RASAL3**					---	NM	NM	MN	NM	NM
**SYNGAP1**						---	NS	NS	NS	NM
**RASA2**							---	NS	NS	NM
**RASA3**								---	NS	NM
**DAB2IP**									---	NM
**RASA4**										---

Bladder Urothelial Carcinoma (BGI, Nature Genetics 2013)/n=99 (WES=99)

Mutation rate: 10.1%

Gene	NF1	RASA2	RASAL2	SYNGAP1	DAB2IP	RASA1	RASA3	RASA4	RASAL1	RASAL3
**NF1**	---	NS	NS	NS	NM	NM	NM	NM	NM	NM
**RASA2**		---	NS	NS	NM	NM	NM	NM	NM	NM
**RASAL2**			---	NS	NM	NM	NM	NM	NM	NM
**SYNGAP1**				---	NM	NM	NM	NM	NM	NM
**DAB2IP**					---	NM	NM	NM	NM	NM
**RASA1**						---	NM	NM	NM	NM
**RASA3**							---	NM	NM	NM
**RASA4**								---	NM	NM
**RASAL1**									---	NM
**RASAL3**										---

Bladder Urothelial Carcinoma (TCGA, Nature 2014)/n=130 (WES=130)

Mutation rate: 19.2%

Gene	NF1	RASA1	RASA3	RASAL2	DAB2IP	SYNGAP1	RASA2	RASAL1	RASAL3	RASA4
**NF1**	---	NS	NS	NS	NS	NS	NS	NS	NM	NM
**RASA1**		---	NS	NS	NS	NS	NS	NS	NM	NM
**RASA3**			---	NS	0.046	NS	NS	NS	NM	NM
**RASAL2**				---	NS	NS	NS	NS	NM	NM
**DAB2IP**					---	NS	NS	NS	NM	NM
**SYNGAP1**						---	NS	NS	NM	NM
**RASA2**							---	NS	NM	NM
**RASAL1**								---	NM	NM
**RASAL3**									---	NM
**RASA4**										---

Liver Hepatocellular Caicionma (TCGA, provisional)/n=198

Mutation rate=10.1%

Gene	NF1	SYNGAP1	RASAL2	DAB2IP	RASA1	RASA2	RASA3	RASA4	RASAL1	RASAL3
**NF1**	---	NS	NS	NS	NS	NM	NS	NM	NS	NM
**SYNGAP1**		---	NS	NS	NS	NM	NS	NM	NS	NM
**RASAL2**			---	NS	NS	NM	NS	NM	NS	NM
**DAB2IP**				---	NS	NM	NS	NM	0.045	NM
**RASA1**					---	NM	NS	NM	NS	NM
**RASA2**						---	NM	NM	NM	NM
**RASA3**							---	NM	NS	NM
**RASA4**								---	NM	NM
**RASAL1**									---	NM
**RASAL3**										---

Glioblastoma (TCGA, Cell 2013)/n=291

Mutation rate: 10.3%

Gene	NF1	DAB2IP	RASAL3	RASAL1	SYNGAP1	RASA1	RASA2	RASA3	RASA4	RASAL2
NF1	---	NS	NM	NS	NS	NM	NM	NM	NM	NM
DAB2IP		---	NM	NS	NS	NM	NM	NM	NM	NM
RASAL3			---	NM	NM	NM	NM	NM	NM	NM
RASAL1				---	NS	NM	NM	NM	NM	NM
SYNGAP1					---	NM	NM	NM	NM	NM
RASA1						---	NM	NM	NM	NM
RASA2							---	NM	NM	NM
RASA3								---	NM	NM
RASA4									---	NM
RASAL2										---

Glioblastoma (TCGA, Nature 2008)/n=91

Mutation rate: 14.3%

Gene	NF1	DAB2IP	RASA1	RASA2	RASA3	RASA4	RASAL1	RASAL2	RASAL3	SYNGAP1
NF1	---	NM	NM	NM	NM	NM	NM	NM	NM	NM
DAB2IP		---	NM	NM	NM	NM	NM	NM	NM	NM
RASA1			---	NM	NM	NM	NM	NM	NM	NM
RASA2				---	NM	NM	NM	NM	NM	NM
RASA3					---	NM	NM	NM	NM	NM
RASA4						---	NM	NM	NM	NM
RASAL1							---	NM	NM	NM
RASAL2								---	NM	NM
RASAL3									---	NM
SYNGAP1										---

Glioblastoma Multiforme (TCGA, provisional)/n=290

Mutation rate: 12.4%

Gene	NF1	DAB2IP	RASA1	RASA2	RASA3	RASA4	RASAL1	RASAL2	RASAL3	SYNGAP1
NF1	---	NS	NM	NM	NM	NM	NS	NS	NM	NS
DAB2IP		---	NM	NM	NM	NM	NS	NS	NM	NS
RASA1			---	NM	NM	NM	NM	NS	NM	NS
RASA2				---	NM	NM	NM	NM	NM	NM
RASA3					---	NM	NM	NM	NM	NM
RASA4						---	NM	NM	NM	NM
RASAL1							---	NS	NM	NS
RASAL2								---	NM	NS
RASAL3									---	NM
SYNGAP1										---

Head and Neck Squamous Cell Carcinoma (TCGA, provisional)/n=306

Mutation rate: 15.7%

Gene	RASA1	NF1	RASA2	RASAL1	RASA3	SYNGAP1	RASAL2	DAB2IP	RASA4	RASAL3
**RASA1**	---	NS	NS	NS	NS	NS	NS	NS	NM	NM
**NF1**		---	NS	NS	NS	NS	NS	NS	NM	NM
**RASA2**			---	NS	NS	NS	NS	NS	NM	NM
**RASAL1**				---	NS	NS	NS	NS	NM	NM
**RASA3**					---	NS	NS	NS	NM	NM
**SYNGAP1**						---	NS	NS	NM	NM
**RASAL2**							---	NS	NM	NM
**DAB2IP**								---	NM	NM
**RASA4**									---	NM
**RASAL3**										---

Head and Neck Squamous Cell Carcinoma (TCGA, in revision)/n=279

Mutation rate: 14%

Gene	RASA1	NF1	RASA2	RASAL1	RASA3	SYNGAP1	RASAL2	DAB2IP	RASA4	RASAL3
**RASA1**	---	NS	NS	NS	NS	NS	NS	NM	NM	NM
**NF1**		---	NS	NS	NS	NS	NS	NM	NM	NM
**RASA2**			---	NS	NS	NS	NS	NM	NM	NM
**RASAL1**				---	NS	NS	NS	NM	NM	NM
**RASA3**					---	NS	NS	NM	NM	NM
**SYNGAP1**						---	NS	NM	NM	NM
**RASAL2**							---	NM	NM	NM
**DAB2IP**								---	NM	NM
**RASA4**									---	NM
**RASAL3**										---

Ovarian Serous Cystadenocarcinoma (TCGA, Provisional)/n=316

Mutation rate: 10.8%

Gene	NF1	RASAL1	SYNGAP1	RASA3	RASA2	RASAL2	DAB2IP	RASA1	RASA4	RASAL3
**NF1**	---	NS	NS	NS	NS	NS	NS	NS	NM	NM
**RASAL1**		---	NS	NS	NS	NS	NS	NS	NM	NM
**SYNGAP1**			---	NS	NS	NS	NS	NS	NM	NM
**RASA3**				---	NS	NS	NS	NS	NM	NM
**RASA2**					---	NS	NS	NS	NM	NM
**RASAL2**						---	NS	NS	NM	NM
**DAB2IP**							---	NS	NM	NM
**RASA1**								---	NM	NM
**RASA4**									---	NM
**RASAL3**										---

Adrenocortical Carcinoma (TCGA, Provisional)/n=90

Mutation rate: 12.2%

Gene	NF1	SYNGAP1	RASAL2	DAB2IP	RASA1	RASA2	RASA3	RASA4	RASAL1	RASAL3
**NF1**	---	NS	NS	NM	NM	NM	NM	NM	NM	NM
**SYNGAP1**		---	NS	NM	NM	NM	NM	NM	NM	NM
**RASAL2**			---	NM	NM	NM	NM	NM	NM	NM
**DAB2IP**				---	NM	NM	NM	NM	NM	NM
**RASA1**					---	NM	NM	NM	NM	NM
**RASA2**						---	NM	NM	NM	NM
**RASA3**							---	NM	NM	NM
**RASA4**								---	NM	NM
**RASAL1**									---	NM
**RASAL3**										---
