# Supplementary material for: Clinical application of genomic profiling to find druggable targets for adolescent and young adult (AYA) cancer patients with metastasis
Source: BMC Cancer. 2016 Feb 29;16:170. doi: 10.1186/s12885-016-2209-1 (PMC4772349; doi:10.1186/s12885-016-2209-1)
Supplement: Supplementary file 8 — EML4-ALK fusion in AYA09. (PDF 128 kb) [file 12885_2016_2209_MOESM8_ESM.pdf]

Table S7. EML4-ALK fusion in AYA09

Gene 1					Gene 2					Score*
Chr	Start	End	Gene	Strand	Chr	Start	End	Gene	Strand	
2	42396489	42491870	EML4	+	2	29415639	29446393	ALK	-	4

*from chimerascan: Total fragments supporting chimera
