# Supplementary material for: Clinical application of genomic profiling to find druggable targets for adolescent and young adult (AYA) cancer patients with metastasis
Source: BMC Cancer. 2016 Feb 29;16:170. doi: 10.1186/s12885-016-2209-1 (PMC4772349; doi:10.1186/s12885-016-2209-1)
Supplement: Supplementary file 10 — CVE list. (PDF 285 kb) [file 12885_2016_2209_MOESM10_ESM.pdf]

Table S8. CVE list

#	Gene Symbol	Cancer gene census	Vogelstein list	Elledge list
1	ABCA10	-	-	O
2	ABCB1	-	-	O
3	ABI1	O	-	O
4	ABL1	O	O	-
5	ABL2	O	-	-
6	ACADS	-	-	O
7	ACBD3	-	-	O
8	ACSL3	O	-	-
9	ACVR1B	-	O	O
10	ACVR2A	-	-	O
11	ADAMTS19	-	-	O
12	ADRBK2	-	-	O
13	AF15Q14	O	-	-
14	AF1Q	O	-	-
15	AF3p21	O	-	-
16	AF5q31	O	-	-
17	AFF4	-	-	O
18	AJUBA	-	-	O
19	AK8	-	-	O
20	AKAP9	O	-	-
21	AKT1	O	O	O
22	AKT2	O	-	-
23	ALDH1L2	-	-	O
24	ALDH2	O	-	-
25	ALK	O	O	O
26	ALO17	O	-	-
27	AMBRA1	-	-	O
28	AMER1	-	-	O
29	AMOT	-	-	O
30	ANKRD46	-	-	O
31	ANO3	-	-	O
32	AOAH	-	-	O
33	APC	O	O	O
34	APPBP2	-	-	O
35	AQP2	-	-	O
36	AR	-	O	-
37	ARF4	-	-	O
38	ARHGAP35	-	-	O
39	ARHGAP5	-	-	O
40	ARHGEF12	O	-	-
41	ARHGEF33	-	-	O
42	ARHH	O	-	-
43	ARID1A	O	O	O
44	ARID1B	-	O	O
45	ARID2	O	O	O
46	ARNT	O	-	-

47	ASCC3	-	-	O
48	ASPSCR1	O	-	-
49	ASXL1	O	O	O
50	ASXL2	-	-	O
51	ATAD2	-	-	O
52	ATF1	O	-	-
53	ATG5	-	-	O
54	ATIC	O	-	-
55	ATM	O	O	O
56	ATR	-	-	O
57	ATRX	O	O	O
58	AXIN1	O	O	O
59	B2M	-	O	O
60	B3GNT5	-	-	O
61	BAP1	O	O	O
62	BAX	-	-	O
63	BCL10	O	-	O
64	BCL11A	O	-	-
65	BCL11B	O	-	-
66	BCL2	O	O	-
67	BCL3	O	-	-
68	BCL5	O	-	-
69	BCL6	O	-	-
70	BCL7A	O	-	-
71	BCL9	O	-	-
72	BCLAF1	-	-	O
73	BCOR	O	O	O
74	BCR	O	-	-
75	BHD	O	-	-
76	BIRC3	O	-	-
77	BIRC6	-	-	O
78	BLM	O	-	-
79	BMPR1A	O	-	-
80	BMPR2	-	-	O
81	BRAF	O	O	O
82	BRCA1	O	O	O
83	BRCA2	O	O	O
84	BRD3	O	-	-
85	BRD4	O	-	-
86	BRD7	-	-	O
87	BRE	-	-	O
88	BRIP1	O	-	-
89	BRSK1	-	-	O
90	BRWD3	-	-	O
91	BTBD11	-	-	O
92	BTBD7	-	-	O
93	BTG1	O	-	-
94	BTG3	-	-	O

95	BUB1B	O	-	-
96	BZW2	-	-	O
97	C12orf9	O	-	-
98	C15orf21	O	-	-
99	C15orf55	O	-	-
100	C16orf45	-	-	O
101	C16orf75	O	-	-
102	C16orf80	-	-	O
103	C16orf87	-	-	O
104	C2CD5	-	-	O
105	C2orf44	O	-	-
106	C3orf27	-	-	O
107	CACNG3	-	-	O
108	CACUL1	-	-	O
109	CADPS	-	-	O
110	CAMTA1	O	-	-
111	CANT1	O	-	-
112	CAPRIN2	-	-	O
113	CARD11	O	O	-
114	CARM1	-	-	O
115	CARS	O	-	-
116	CASP8	-	O	O
117	CBFA2T1	O	-	-
118	CBFA2T3	O	-	-
119	CBFB	O	-	O
120	CBL	O	O	-
121	CBLB	O	-	-
122	CBLC	O	-	-
123	CCAR1	-	-	O
124	CCDC120	-	-	O
125	CCDC132	-	-	O
126	CCDC144NL	-	-	O
127	CCDC36	-	-	O
128	CCDC38	-	-	O
129	CCDC6	O	-	-
130	CCDC88A	-	-	O
131	CCNB1IP1	O	-	-
132	CCND1	O	O	-
133	CCND2	O	-	-
134	CCND3	O	-	O
135	CCNE1	O	-	-
136	CD163	-	-	O
137	CD273	O	-	-
138	CD274	O	-	-
139	CD58	-	-	O
140	CD74	O	-	-
141	CD79A	O	-	-
142	CD79B	O	-	-

143	CDC27	-	-	O
144	CDC73	-	O	O
145	CDH1	O	O	O
146	CDH11	O	-	-
147	CDH7	-	-	O
148	CDK12	O	-	O
149	CDK4	O	-	-
150	CDK6	O	-	-
151	CDKN1A	-	-	O
152	CDKN1B	-	-	O
153	CDKN2A	O	O	O
154	CDKN2a(p14)	O	-	-
155	CDKN2C	O	O	-
156	CDX2	O	-	-
157	CEBPA	O	O	O
158	CEP1	O	-	-
159	CEP128	-	-	O
160	CERK	-	-	O
161	CHCHD7	O	-	-
162	CHD2	-	-	O
163	CHD3	-	-	O
164	CHD4	-	-	O
165	CHD8	-	-	O
166	CHEK2	O	-	-
167	CHIC2	O	-	-
168	CHN1	O	-	-
169	CIC	O	O	O
170	CIITA	O	-	-
171	CIZ1	-	-	O
172	CLGN	-	-	O
173	CLTC	O	-	-
174	CLTCL1	O	-	-
175	CMAS	-	-	O
176	CMKOR1	O	-	-
177	CNOT3	O	-	O
178	COL1A1	O	-	-
179	COL4A2	-	-	O
180	COL5A2	-	-	O
181	COL8A2	-	-	O
182	COL9A1	-	-	O
183	COPEB	O	-	-
184	COQ9	-	-	O
185	COX6C	O	-	-
186	CPB1	-	-	O
187	CPEB2	-	-	O
188	CREB1	O	-	-
189	CREB3L1	O	-	-
190	CREB3L2	O	-	-

191	CREBBP	O	O	O
192	CRIPAK	-	-	O
193	CRISPLD1	-	-	O
194	CRLF2	O	O	-
195	CRTC3	O	-	-
196	CSDE1	-	-	O
197	CSF1R	-	O	-
198	CSF3R	O	-	-
199	CSMD3	-	-	O
200	CSNK1E	-	-	O
201	CSNK2A1	-	-	O
202	CTCF	-	-	O
203	CTDNEP1	-	-	O
204	CTNNB1	O	O	O
205	CTNND1	-	-	O
206	CUL1	-	-	O
207	CUL3	-	-	O
208	CYLD	O	O	O
209	D10S170	O	-	-
210	DAAM1	-	-	O
211	DACH1	-	-	O
212	DAXX	O	O	-
213	DCLK1	-	-	O
214	DDB2	O	-	-
215	DDIT3	O	-	-
216	DDX10	O	-	-
217	DDX3X	-	-	O
218	DDX5	O	-	-
219	DDX6	O	-	-
220	DEK	O	-	-
221	DENND6A	-	-	O
222	DEPDC5	-	-	O
223	DICER1	O	-	O
224	DIP2C	-	-	O
225	DKK2	-	-	O
226	DNER	-	-	O
227	DNM2	O	-	-
228	DNMT1	-	O	-
229	DNMT3A	O	O	O
230	DOPEY1	-	-	O
231	DPY19L4	-	-	O
232	DTX1	-	-	O
233	DUSP16	-	-	O
234	DUX4	O	-	-
235	DYRK1A	-	-	O
236	EBF1	O	-	-
237	EBPL	-	-	O
238	ECT2L	O	-	-

239	EEF1B2	-	-	O
240	EGFR	O	O	O
241	EIF1AX	-	-	O
242	EIF2AK3	-	-	O
243	EIF4A2	O	-	-
244	ELF3	-	-	O
245	ELF4	O	-	-
246	ELK4	O	-	-
247	ELKS	O	-	-
248	ELL	O	-	-
249	ELN	O	-	-
250	EMG1	-	-	O
251	EML4	O	-	-
252	ENAH	-	-	O
253	EP300	O	O	O
254	EPB41L4A	-	-	O
255	EPHA2	-	-	O
256	EPHA6	-	-	O
257	EPS15	O	-	-
258	ERBB2	O	O	O
259	ERBB3	-	-	O
260	ERCC2	O	-	-
261	ERCC3	O	-	-
262	ERCC4	O	-	-
263	ERCC5	O	-	-
264	ERG	O	-	-
265	ERRFI1	-	-	O
266	ETV1	O	-	-
267	ETV4	O	-	-
268	ETV5	O	-	-
269	ETV6	O	-	-
270	EVI1	O	-	-
271	EWSR1	O	-	-
272	EXO5	-	-	O
273	EXT1	O	-	-
274	EXT2	O	-	-
275	EZH2	O	O	O
276	EZR	O	-	-
277	FACL6	O	-	-
278	FAM123B	-	O	-
279	FAM22A	O	-	-
280	FAM22B	O	-	-
281	FAM46C	O	-	-
282	FAM58A	-	-	O
283	FAM8A1	-	-	O
284	FANCA	O	-	-
285	FANCC	O	-	-
286	FANCD2	O	-	-

287	FANCE	O	-	-
288	FANCF	O	-	-
289	FANCG	O	-	-
290	FANCM	-	-	O
291	FAS	-	-	O
292	FAT1	-	-	O
293	FBXO11	O	-	-
294	FBXW7	O	O	O
295	FCGR2B	O	-	-
296	FEV	O	-	-
297	FGFR1	O	-	-
298	FGFR1OP	O	-	-
299	FGFR2	O	O	O
300	FGFR3	O	O	O
301	FH	O	-	-
302	FHIT	O	-	-
303	FIP1L1	O	-	-
304	FLI1	O	-	-
305	FLJ27352	O	-	-
306	FLT3	O	O	O
307	FMNL3	-	-	O
308	FNBP1	O	-	-
309	FOSL2	-	-	O
310	FOXA1	-	-	O
311	FOXL2	O	O	-
312	FOXO1A	O	-	-
313	FOXO3A	O	-	-
314	FOXP1	O	-	-
315	FSTL3	O	-	-
316	FUBP1	O	O	O
317	FUS	O	-	-
318	FVT1	O	-	-
319	GABRA1	-	-	O
320	GABRA2	-	-	O
321	GABRA5	-	-	O
322	GABRG1	-	-	O
323	GAPVD1	-	-	O
324	GAS7	O	-	-
325	GATA1	O	O	-
326	GATA2	O	O	-
327	GATA3	O	O	O
328	GGCT	-	-	O
329	GGNBP2	-	-	O
330	GIGYF2	-	-	O
331	GJB3	-	-	O
332	GK2	-	-	O
333	GLTSCR1	-	-	O
334	GMPS	O	-	-

335	GNA11	O	O	-
336	GNA15	-	-	O
337	GNAQ	O	O	-
338	GNAS	O	O	O
339	GNG4	-	-	O
340	GNPTAB	-	-	O
341	GOLGA5	O	-	-
342	GOPC	O	-	-
343	GOT2	-	-	O
344	GPC3	O	-	-
345	GPHN	O	-	-
346	GPR141	-	-	O
347	GPR174	-	-	O
348	GPS2	-	-	O
349	GRAF	O	-	-
350	GRIA2	-	-	O
351	GRIA3	-	-	O
352	GRID1	-	-	O
353	GRID2	-	-	O
354	GRM5	-	-	O
355	GRXCR1	-	-	O
356	GSE1	-	-	O
357	GTF2I	-	-	O
358	H3F3A	O	O	-
359	HARS2	-	-	O
360	HAUS8	-	-	O
361	HCMOGT-1	O	-	-
362	HDAC2	-	-	O
363	HEAB	O	-	-
364	HERC1	-	-	O
365	HERPUD1	O	-	-
366	HEY1	O	-	-
367	HGF	-	-	O
368	HIP1	O	-	-
369	HIST1H2BF	-	-	O
370	HIST1H3B	O	O	O
371	HIST1H4I	O	-	-
372	HLA-A	-	-	O
373	HLA-B	-	-	O
374	HLA-DRB1	-	-	O
375	HLF	O	-	O
376	HLXB9	O	-	-
377	HMCN1	-	-	O
378	HMGA1	O	-	-
379	HMGA2	O	-	-
380	HNF1A	-	O	O
381	HNRNPA2B1	O	-	-
382	HNRNPD	-	-	O

383	HOOK3	O	-	-
384	HOXA11	O	-	-
385	HOXA13	O	-	-
386	HOXA9	O	-	-
387	HOXC11	O	-	-
388	HOXC13	O	-	-
389	HOXD11	O	-	-
390	HOXD13	O	-	-
391	HRAS	O	O	O
392	HRPT2	O	-	-
393	HS6ST1	-	-	O
394	HSPCA	O	-	-
395	HSPCB	O	-	-
396	ID3	-	-	O
397	IDH1	O	O	O
398	IDH2	O	O	O
399	IFITM1	-	-	O
400	IFNGR2	-	-	O
401	IGH@	O	-	-
402	IGK@	O	-	-
403	IGL@	O	-	-
404	IKZF1	O	O	-
405	IL2	O	-	-
406	IL21	-	-	O
407	IL21R	O	-	-
408	IL32	-	-	O
409	IL5RA	-	-	O
410	IL6ST	O	-	-
411	IL7R	O	-	-
412	INO80	-	-	O
413	INPPL1	-	-	O
414	INTS7	-	-	O
415	IREB2	-	-	O
416	IRF2	-	-	O
417	IRF4	O	-	-
418	IRTA1	O	-	-
419	ITK	O	-	-
420	ITM2C	-	-	O
421	IWS1	-	-	O
422	JAK1	O	O	-
423	JAK2	O	O	-
424	JAK3	O	O	-
425	JAKMIP2	-	-	O
426	JAZF1	O	-	-
427	JMJD1C	-	-	O
428	JUN	O	-	O
429	KANSL1	-	-	O
430	KAT8	-	-	O

431	KBTBD7	-	-	O
432	KCND3	-	-	O
433	KCNJ5	O	-	-
434	KCNMB2	-	-	O
435	KCNQ5	-	-	O
436	KCNT2	-	-	O
437	KDM3B	-	-	O
438	KDM5A	O	-	-
439	KDM5C	O	O	O
440	KDM6A	O	O	O
441	KDR	O	-	-
442	KEAP1	-	-	O
443	KIAA0907	-	-	O
444	KIAA1549	O	-	-
445	KIF5B	O	-	-
446	KIT	O	O	-
447	KLF4	O	O	-
448	KLHL5	-	-	O
449	KLK2	O	-	-
450	KLK8	-	-	O
451	KMT2A	-	-	O
452	KMT2B	-	-	O
453	KMT2C	-	-	O
454	KMT2D	-	-	O
455	KRAS	O	O	O
456	KRT15	-	-	O
457	KRTAP4-11	-	-	O
458	KRTAP4-5	-	-	O
459	KTN1	O	-	-
460	LAF4	O	-	-
461	LARP4B	-	-	O
462	LASP1	O	-	-
463	LCK	O	-	-
464	LCP1	O	-	-
465	LCX	O	-	-
466	LDB1	-	-	O
467	LEMD2	-	-	O
468	LHFP	O	-	-
469	LHFPL1	-	-	O
470	LIFR	O	-	-
471	LIMCH1	-	-	O
472	LIN9	-	-	O
473	LMO1	O	O	-
474	LMO2	O	-	-
475	LPA	-	-	O
476	LPHN3	-	-	O
477	LPP	O	-	-
478	LRIG3	O	-	-

479	LSM11	-	-	O
480	LUC7L3	-	-	O
481	LUM	-	-	O
482	LYL1	O	-	-
483	LZTR1	-	-	O
484	MADH4	O	-	-
485	MAF	O	-	-
486	MAFB	O	-	-
487	MAGED1	-	-	O
488	MAGI2	-	-	O
489	MALT1	O	-	-
490	MAML2	O	-	-
491	MAP2K1	O	O	O
492	MAP2K2	O	-	-
493	MAP2K4	O	O	O
494	MAP3K1	-	O	O
495	MAP3K4	-	-	O
496	MAPK1	-	-	O
497	MAPK8	-	-	O
498	MAPK8IP2	-	-	O
499	MARCO	-	-	O
500	MAX	O	-	O
501	MB21D2	-	-	O
502	MBD1	-	-	O
503	MBD6	-	-	O
504	MBOAT2	-	-	O
505	MDGA2	-	-	O
506	MDM2	O	O	-
507	MDM4	O	O	-
508	MDS1	O	-	-
509	MDS2	O	-	-
510	MECOM	-	-	O
511	MECT1	O	-	-
512	MED12	O	O	O
513	MED23	-	-	O
514	MEF2B	-	-	O
515	MEF2BNB-MEF2B	-	-	O
516	MEN1	O	O	O
517	MET	O	O	-
518	METTL14	-	-	O
519	MFAP5	-	-	O
520	MFF	-	-	O
521	MFGE8	-	-	O
522	MGA	-	-	O
523	MICU3	-	-	O
524	MITF	O	-	-
525	MKL1	O	-	-
526	MKRN1	-	-	O

527	MLF1	O	-	-
528	MLH1	O	O	-
529	MLL	O	-	-
530	MLL2	O	O	-
531	MLL3	O	O	-
532	MLLT1	O	-	-
533	MLLT10	O	-	-
534	MLLT11	-	-	O
535	MLLT2	O	-	-
536	MLLT3	O	-	-
537	MLLT4	O	-	O
538	MLLT6	O	-	-
539	MLLT7	O	-	-
540	MN1	O	-	-
541	MOAP1	-	-	O
542	MORC4	-	-	O
543	MPL	O	O	-
544	MRPL32	-	-	O
545	MRPL49	-	-	O
546	MS4A8	-	-	O
547	MSF	O	-	-
548	MSH2	O	O	-
549	MSH6	O	O	-
550	MSI2	O	-	-
551	MSN	O	-	-
552	MTCP1	O	-	-
553	MTFR1L	-	-	O
554	MTOR	-	-	O
555	MUC1	O	-	-
556	MUTYH	O	-	-
557	MYB	O	-	-
558	MYC	O	O	O
559	MYCL1	O	O	-
560	MYCN	O	O	O
561	MYD88	O	O	O
562	MYF5	-	-	O
563	MYH11	O	-	-
564	MYH2	-	-	O
565	MYH9	O	-	-
566	MYO1B	-	-	O
567	MYO3A	-	-	O
568	MYO6	-	-	O
569	MYOT	-	-	O
570	MYST4	O	-	-
571	NAA25	-	-	O
572	NACA	O	-	-
573	NBPF1	-	-	O
574	NBPF10	-	-	O

575	NBS1	O	-	-
576	NCOA1	O	-	-
577	NCOA2	O	-	-
578	NCOA3	-	O	-
579	NCOA4	O	-	-
580	NCOR1	-	O	O
581	NCOR2	-	-	O
582	NDRG1	O	-	-
583	NDUFS5	-	-	O
584	NEK9	-	-	O
585	NENF	-	-	O
586	NF1	O	O	O
587	NF2	O	O	O
588	NFATC4	-	-	O
589	NFE2L2	O	O	O
590	NFIB	O	-	-
591	NFKB2	O	-	-
592	NIN	O	-	-
593	NIPBL	-	-	O
594	NKX2-1	O	O	-
595	NNMT	-	-	O
596	NONO	O	-	-
597	NOTCH1	O	O	O
598	NOTCH2	O	O	O
599	NPM1	O	O	O
600	NR4A3	O	-	-
601	NRAP	-	-	O
602	NRAS	O	O	O
603	NRG3	-	-	O
604	NSD1	O	-	O
605	NSFL1C	-	-	O
606	NSMCE1	-	-	O
607	NT5C2	O	-	-
608	NTRK1	O	-	-
609	NTRK3	O	-	-
610	NUMA1	O	-	-
611	NUP214	O	-	-
612	NUP98	O	-	O
613	NUTM2F	-	-	O
614	OLIG2	O	-	-
615	OMD	O	-	-
616	OPRM1	-	-	O
617	OR4M2	-	-	O
618	OR5I1	-	-	O
619	P2RY8	O	-	-
620	PABPC3	-	-	O
621	PAFAH1B2	O	-	-
622	PALB2	O	-	-

623	PAX3	O	-	-
624	PAX5	O	O	-
625	PAX7	O	-	-
626	PAX8	O	-	-
627	PBRM1	O	O	O
628	PBX1	O	-	-
629	PCDH7	-	-	O
630	PCM1	O	-	-
631	PCMTD1	-	-	O
632	PCSK7	O	-	-
633	PDE4DIP	O	-	-
634	PDE7B	-	-	O
635	PDGFB	O	-	-
636	PDGFRA	O	O	-
637	PDGFRB	O	-	-
638	PDYN	-	-	O
639	PER1	O	-	-
640	PEX2	-	-	O
641	PGM5	-	-	O
642	PHACTR4	-	-	O
643	PHF6	O	O	O
644	PHOX2B	O	-	-
645	PICALM	O	-	-
646	PIK3CA	O	O	O
647	PIK3CB	-	-	O
648	PIK3R1	O	O	O
649	PIK3R5	-	-	O
650	PIM1	O	-	-
651	PLAC4	-	-	O
652	PLAG1	O	-	-
653	PLCL1	-	-	O
654	PLEKHA6	-	-	O
655	PLEKHM3	-	-	O
656	PLK2	-	-	O
657	PML	O	-	-
658	PMS1	O	-	-
659	PMS2	O	-	-
660	PMX1	O	-	-
661	PNISR	-	-	O
662	PNUTL1	O	-	-
663	POLE	-	-	O
664	POT1	O	-	-
665	POTEG	-	-	O
666	POU2AF1	O	-	-
667	POU5F1	O	-	-
668	PPARG	O	-	-
669	PPM1D	-	-	O
670	PPP1R9A	-	-	O

671	PPP2R1A	O	O	O
672	PPP3CC	-	-	O
673	PPP6C	-	-	O
674	PRCC	O	-	-
675	PRDM1	O	O	-
676	PRDM16	O	-	-
677	PRF1	O	-	-
678	PRKAR1A	O	-	-
679	PRKCI	-	-	O
680	PRKRA	-	-	O
681	PRO1073	O	-	-
682	PRPF40A	-	-	O
683	PRPF8	-	-	O
684	PRRC2C	-	-	O
685	PRRX1	-	-	O
686	PSIP1	-	-	O
687	PSIP2	O	-	-
688	PSMC3IP	-	-	O
689	PSPH	-	-	O
690	PTCH	O	-	-
691	PTCH1	-	O	O
692	PTEN	O	O	O
693	PTN	-	-	O
694	PTPN11	O	O	O
695	PTPRC	O	-	-
696	PTPRF	-	-	O
697	PTPRK	-	-	O
698	PTPRU	-	-	O
699	PUS7	-	-	O
700	RAB5EP	O	-	-
701	RAC1	O	-	O
702	RAD21	O	-	O
703	RAD51L1	O	-	-
704	RAF1	O	-	-
705	RALGDS	O	-	-
706	RANBP17	O	-	-
707	RAP1GDS1	O	-	-
708	RAPGEF6	-	-	O
709	RARA	O	-	-
710	RASA1	-	-	O
711	RB1	O	O	O
712	RBBP6	-	-	O
713	RBM10	-	-	O
714	RBM15	O	-	O
715	RBM26	-	-	O
716	RBM39	-	-	O
717	RBMX	-	-	O
718	RECQL4	O	-	-

719	REL	O	-	-
720	RERE	-	-	O
721	RET	O	O	-
722	REXO2	-	-	O
723	RGPD3	-	-	O
724	RGS12	-	-	O
725	RHBDL3	-	-	O
726	RHOA	-	-	O
727	RIMS2	-	-	O
728	RIT1	-	-	O
729	RNF111	-	-	O
730	RNF43	O	O	O
731	ROS1	O	-	-
732	RPL10	O	-	-
733	RPL18	-	-	O
734	RPL22	O	-	O
735	RPL5	O	-	O
736	RPN1	O	-	-
737	RRAS2	-	-	O
738	RUFY2	-	-	O
739	RUNDC2A	O	-	-
740	RUNX1	O	O	O
741	RUNXBP2	O	-	-
742	RXRB	-	-	O
743	SAP30	-	-	O
744	SBDS	O	-	-
745	SBNO1	-	-	O
746	SCN3A	-	-	O
747	SCRN3	-	-	O
748	SDC4	O	-	-
749	SDH5	O	-	-
750	SDHB	O	-	-
751	SDHC	O	-	-
752	SDHD	O	-	-
753	SEC22A	-	-	O
754	SEC63	-	-	O
755	SEMG2	-	-	O
756	SEPT14	-	-	O
757	SEPT6	O	-	-
758	SESTD1	-	-	O
759	SET	O	-	-
760	SETBP1	O	O	-
761	SETD2	O	O	O
762	SF3B1	O	O	O
763	SFPQ	O	-	O
764	SFRS3	O	-	-
765	SH2B3	O	-	-
766	SH3GL1	O	-	-

767	SHB	-	-	O
768	SIAH2	-	-	O
769	SIK3	-	-	O
770	SIL	O	-	-
771	SIN3A	-	-	O
772	SKAP2	-	-	O
773	SKP2	-	O	-
774	SLC23A2	-	-	O
775	SLC34A2	O	-	-
776	SLC35G3	-	-	O
777	SLC45A3	O	-	-
778	SMAD2	-	O	O
779	SMAD4	-	O	O
780	SMARCA1	-	-	O
781	SMARCA2	-	-	O
782	SMARCA4	O	O	O
783	SMARCB1	O	O	O
784	SMARCE1	O	-	-
785	SMC4	-	-	O
786	SMO	O	O	O
787	SMOC2	-	-	O
788	SNRPN	-	-	O
789	SOCS1	O	O	-
790	SOCS5	-	-	O
791	SOS1	-	-	O
792	SOX17	-	-	O
793	SOX2	O	-	-
794	SOX21	-	-	O
795	SOX9	-	O	O
796	SPATA6	-	-	O
797	SPCS1	-	-	O
798	SPEN	-	-	O
799	SPOP	-	O	O
800	SPPL3	-	-	O
801	SPRED1	-	-	O
802	SPRED2	-	-	O
803	SPSB2	-	-	O
804	SRC	-	-	O
805	SRGAP3	O	-	-
806	SRSF11	-	-	O
807	SRSF2	O	O	-
808	SS18	O	-	-
809	SS18L1	O	-	-
810	SSX1	O	-	-
811	SSX2	O	-	-
812	SSX4	O	-	-
813	STAG2	-	O	O
814	STAT3	O	-	O

815	STAT5B	O	-	-
816	STK11	O	O	O
817	STL	O	-	-
818	STRIP2	-	-	O
819	STX7	-	-	O
820	STXBP1	-	-	O
821	STXBP6	-	-	O
822	SUFU	O	-	-
823	SULT1C4	-	-	O
824	SUZ12	O	-	O
825	SYK	O	-	-
826	TAF1	-	-	O
827	TAF15	O	-	-
828	TAL1	O	-	-
829	TAL2	O	-	-
830	TAOK1	-	-	O
831	TBC1D10C	-	-	O
832	TBL1XR1	-	-	O
833	TBL1XR1	O	-	-
834	TBX15	-	-	O
835	TBX18	-	-	O
836	TBX3	-	-	O
837	TCEA1	O	-	-
838	TCEB3	-	-	O
839	TCF1	O	-	-
840	TCF12	O	-	O
841	TCF3	O	-	-
842	TCF7L2	O	-	O
843	TCL1A	O	-	-
844	TCL6	O	-	-
845	TERT	O	-	-
846	TES	-	-	O
847	TET2	O	O	O
848	TEX11	-	-	O
849	TFE3	O	-	-
850	TFEB	O	-	-
851	TFG	O	-	-
852	TFPT	O	-	-
853	TFRC	O	-	-
854	TGFBR2	-	-	O
855	THRAP3	O	-	O
856	THUMPD3	-	-	O
857	TIF1	O	-	-
858	TKTL2	-	-	O
859	TLL1	-	-	O
860	TLL2	-	-	O
861	TLX1	O	-	-
862	TLX3	O	-	-

863	TM2D3	-	-	O
864	TMCO2	-	-	O
865	TMEM11	-	-	O
866	TMEM30A	-	-	O
867	TMPO	-	-	O
868	TMPRSS2	O	-	-
869	TNFAIP3	O	O	-
870	TNFRSF14	O	-	-
871	TNFRSF17	O	-	-
872	TNFRSF6	O	-	-
873	TNIK	-	-	O
874	TNRC6B	-	-	O
875	TOP1	O	-	-
876	TOR1A	-	-	O
877	TOX	-	-	O
878	TP53	O	O	O
879	TP53BP1	-	-	O
880	TPM3	O	-	-
881	TPM4	O	-	-
882	TPR	O	-	-
883	TRA@	O	-	-
884	TRAF3	-	-	O
885	TRAF7	O	O	-
886	TRB@	O	-	-
887	TRD@	O	-	-
888	TRIM23	-	-	O
889	TRIM27	O	-	-
890	TRIM33	O	-	-
891	TRIM48	-	-	O
892	TRIP11	O	-	-
893	TRIP12	-	-	O
894	TRPC6	-	-	O
895	TSC1	O	O	-
896	TSC2	O	-	-
897	TSHR	O	O	-
898	TTC18	-	-	O
899	TTL	O	-	-
900	TUSC3	-	-	O
901	TXNRD1	-	-	O
902	TYRP1	-	-	O
903	U2AF1	O	O	O
904	UBE2QL1	-	-	O
905	UBQLN2	-	-	O
906	UBR5	-	-	O
907	UBR5	O	-	-
908	UGT2A2	-	-	O
909	UGT8	-	-	O
910	UPF3B	-	-	O

911	USP28	-	-	O
912	USP6	O	-	-
913	USP9X	-	-	O
914	VHL	O	O	O
915	VPS13A	-	-	O
916	VTI1A	O	-	-
917	WAC	-	-	O
918	WAS	O	-	-
919	WBP1	-	-	O
920	WBSCR17	-	-	O
921	WDR17	-	-	O
922	WDR33	-	-	O
923	WDR47	-	-	O
924	WHSC1	O	-	-
925	WHSC1L1	O	-	-
926	WIF1	O	-	-
927	WNT11	-	-	O
928	WRN	O	-	-
929	WT1	O	O	O
930	WTX	O	-	-
931	WWC2	-	-	O
932	WWTR1	O	-	-
933	XPA	O	-	-
934	XPC	O	-	-
935	XPO1	O	-	-
936	XPOT	-	-	O
937	XYLT1	-	-	O
938	YLPM1	-	-	O
939	YWHAE	O	-	-
940	ZBTB18	-	-	O
941	ZBTB7A	-	-	O
942	ZC3H13	-	-	O
943	ZC3H18	-	-	O
944	ZFP2	-	-	O
945	ZFP36L1	-	-	O
946	ZFP36L2	-	-	O
947	ZFR	-	-	O
948	ZMYM3	-	-	O
949	ZMYM4	-	-	O
950	ZNF117	-	-	O
951	ZNF145	O	-	-
952	ZNF181	-	-	O
953	ZNF198	O	-	-
954	ZNF234	-	-	O
955	ZNF263	-	-	O
956	ZNF278	O	-	-
957	ZNF292	-	-	O
958	ZNF318	-	-	O

959	ZNF319	-	-	O
960	ZNF331	O	-	-
961	ZNF384	O	-	-
962	ZNF479	-	-	O
963	ZNF521	O	-	-
964	ZNF559	-	-	O
965	ZNF750	-	-	O
966	ZNF799	-	-	O
967	ZNF844	-	-	O
968	ZNF878	-	-	O
969	ZNF9	O	-	-
970	ZNRF3	-	-	O
971	ZRSR2	O	-	-
